# A fluorescent reporter for rapid assessment of autophagic flux reveals unique autophagy signatures during *C. elegans* post-embryonic development and identifies compounds that modulate autophagy

**DOI:** 10.1080/27694127.2024.2371736

**Published:** 2024-07-11

**Authors:** Zachary D. Dawson, Hemalatha Sundaramoorthi, Suk Regmi, Bo Zhang, Stephanie Morrison, Sara M. Fielder, Jessie R. Zhang, Hieu Hoang, David H. Perlmutter, Cliff J. Luke, Gary A. Silverman, Stephen C. Pak

**Affiliations:** Department of Pediatrics, Washington University in St Louis School of Medicine, St Louis, Washington 63110, USA

**Keywords:** Biomarker, high-content screening, LC3, LGG-1, probe, small molecule

## Abstract

Autophagy is important for many physiological processes; and disordered autophagy can contribute to the pathogenesis of a broad range of systemic disorders. *C. elegans* is a useful model organism for studying the genetics of autophagy, however, current methods for studying autophagy are labor-intensive and not readily amenable to high-throughput procedures. Here we describe a fluorescent reporter, GFP::LGG-1::mKate2, which is useful for monitoring autophagic flux in live animals. In the intestine, the fusion protein is processed by endogenous ATG-4 to generate GFP::LGG-1 and mKate2 proteins. We provide data indicating that the GFP:mKate ratio is a suitable readout for measuring cellular autophagic flux. Using this reporter, we measured autophagic flux in L1 larvae to day 7 adult animals. We show that basal autophagic flux is relatively low during larval development but increases markedly in reproductive adults before decreasing with age. Furthermore, we show that wild-type, *eat-2*, and *daf-2* mutant animals have distinct autophagic flux profiles through post-embryonic development. Finally, we demonstrate the utility of this reporter by performing a high-content small molecule screen to identify compounds that alter autophagic flux in *C. elegans*.

## Introduction

Macroautophagy (hereafter referred to as autophagy) is important for many physiological processes and dysfunctional autophagy contributes to the pathogenesis of a broad range of systemic disorders. Consequently, it is critically important to develop research tools for accurately monitoring autophagy. Numerous tools have been developed to monitor autophagy in cells [1–5]. Most of these approaches quantify Atg8/MAP1LC3 (microtubule-associated protein 1 light chain 3, MAP1LC3) commonly referred to as LC3. LC3 is proteolytically processed by the ATG4 enzyme into a form referred to as LC3-I [6,7]. When autophagy is activated, LC3-I is further processed into LC3-II by the addition of a phosphatidylethanolamine (PE) moiety, which is required for anchoring to the phagophore membrane. Since only LC3-II is found in autophagosomes, the relative level of LC3-II (or the ratio of LC3-II to LC3-I) as measured by western blot analysis, can provide information about the number of autophagosomes in the cell.

Fluorescent proteins tagged to the N-terminus of LC3 (e.g., GFP-LC3) have also been used to visualize and quantify autophagosomes in live cells and organisms [5,8–10]. However, static measurements of LC3 or autophagosome number are difficult to interpret, as an increase in number of autophagosomes could arise from either an increase in autophagic activity or a decrease in autophagosome-lysosome processing [1,2,11–13]. Consequently, it is preferable to measure autophagic flux, which is defined as the rate at which autophagic cargo is completely processed from sequestration to its degradation [14,15]. Autophagy reporters for measuring autophagic flux have been described [16–22]. Unlike the GFP-LC3 reporter, the GFP-LC3-mRFP-LC3∆G reporter allows efficient quantification of autophagic flux by measuring the GFP/mRFP ratio [18]. Cleavage of this fusion protein by cellular ATG4 enzymes at the C-terminus of LC3 results in the generation of equimolar amounts of GFP-LC3 and mRFP-LC3∆G. The GFP-LC3 is lipidated and packaged into autophagosomes and consumed by autophagic degradation. In contrast, mRFP-LC3∆G, which lacks a C-terminal glycine residue, fails to get packaged into autophagosomes and thus acts as an internal control. By simply measuring the GFP/mRFP ratio, one can assess the autophagic flux state of the cell at any given time. This GFP-LC3-mRFP-LC3∆G reporter has been demonstrated to work in MEF cells, zebrafish embryos and mice. Recently, a similar reporter was used in *C. elegans* [23], however, extensive characterization of the reporter was not provided.

*C. elegans* is a useful genetic model organism that has played an important role in the identification of novel autophagy genes, such as *epg-2, epg-3, epg-4*, and *epg-5*, which are absent in yeast but conserved in metazoans [24]. Nonetheless, autophagy is challenging to study in *C. elegans* biochemically as antibodies against autophagy-related proteins including LGG-1 (GABARAP-GABARAPL2/GATE-16), LGG-2 (Atg8/MAP1LC3/LC3) and SQST-1 (SQSTM1/p62) are not readily available from commercial sources. As such, the study of autophagy in *C. elegans* for the past two decades has almost exclusively used the GFP::LGG-1 reporter [25,26]. While this reporter has done much to facilitate the study of autophagy in *C. elegans*, autophagy measurements require high resolution imaging and manual quantification of GFP::LGG-1 puncta number which are both labor-intensive and time-consuming. Moreover, since puncta count alone is insufficient for determining whether autophagy is increased or decreased, additional studies (e.g., treatment with chloroquine or injection with bafilomycin A) are required to make inferences about autophagic flux. Other alternative methods for studying autophagy in *C. elegans* have been previously described [17,27]. For example, Chapin et al. [17] utilized a dual fluorescent protein tagged LGG-1 transgene (dFP::LGG-1) connected via a protease sensitive linker. This strategy relies on cleavage of dFP::LGG-1 by lysosomal proteases which releases monomeric fluorescent protein (mFP). By measuring the abundance of mFP relative to dFP::LGG-1 on a western blot, the authors were able to make inferences about autophagic flux. However, this method requires western blotting and densitometry which are labor-intensive and time-consuming. Another approach for measuring autophagic flux was recently described by Ke et al [27] who measured LGG-1::GFP in embryos with or without treatment with the autophagic flux blocker chloroquine. This method involves capturing high resolution confocal images to calculate the GFP fluorescence of the embryos with and without chloroquine treatment. While this method was shown to be useful for measuring autophagic flux in different stages, time-consuming high-resolution microscopy and image analysis were required. More recently, a dual fluorescent mCherry::GFP::LGG-1 reporter was used to study autophagic flux in *C. elegans* [28,29]. Unlike the GFP::LGG-1 reporter which primarily labels autophagosomes, the dual mCherry::GFP::LGG-1 reporter can distinguish autophagosomes (GFP^+^, mCherry ^+^) from autolysosomes (GFP^−^, mCherry ^+^) thus providing additional information about the autophagic vesicular pool. Like the GFP::LGG-1 marker, autophagic flux studies that utilize the dual fluorescence reporter require high-resolution imaging and quantification of GFP^+^ puncta with or without bafilomycin A1 injection into the animal. While this approach has facilitated the study of autophagic flux in aging animals, this methodology is also labor-intensive and low-throughput. Therefore, a fluorescent reporter that provides a simple quantitative readout for autophagic flux would facilitate longitudinal studies of autophagic flux and enable high-throughput approaches for identification of genes and small molecules that modulate autophagy under different conditions.

We describe a GFP::LGG-1::mKate2 autophagic flux reporter (hereafter referred to as AFR) for high-throughput assessment of autophagy in live *C. elegans*. Using this reporter, we measured autophagic flux in all post-embryonic stages from L1 larvae to day 7 adult animals. We reveal that basal autophagic flux starts relatively low during larval development but is markedly enhanced during early adulthood before declining with age. Analysis of different genetic mutants, including *daf-2* and *eat-2*, revealed distinct cellular autophagic flux signatures during post-embryonic development. We further demonstrate the utility of the AFR by conducting a high-content, small molecule screen to identify compounds that modulate autophagic flux in *C. elegans*.

## Results

### Autophagic flux reporter (AFR) generation and characterization

**Figure 1.**
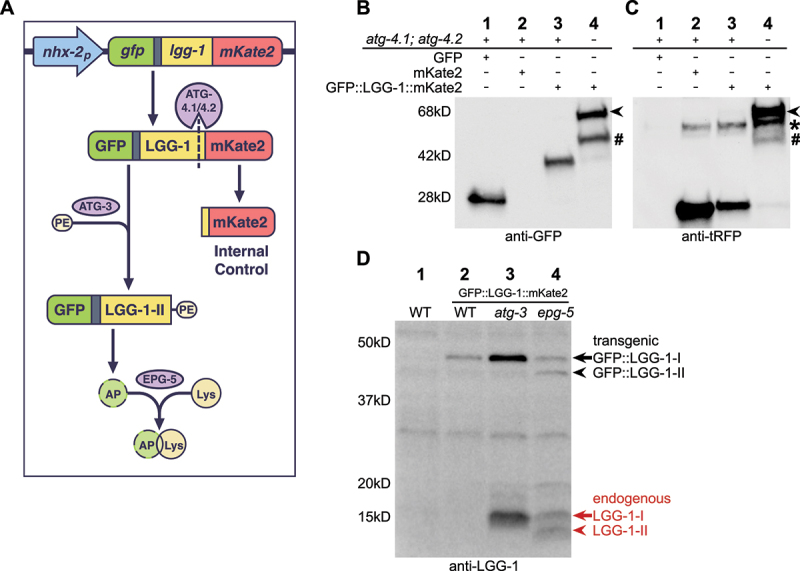
AFR is correctly processed and lipidated by the intestinal cells. (A) A schematic showing how the autophagic flux reporter (AFR) is processed. The reporter is expressed as a fusion protein, GFP::LGG-1::mKate2, which is cleaved by ATG-4.1/-4.2 proteases and lipidated by ATG-3 and the conjugation complex. GFP::LGG-1-PE is incorporated into autophagosomal membranes which fuse with lysosomes in an EPG-5-dependent manner. Cleaved mKate2 remains in the cytosol and acts as the internal control. The GFP:mKate2 ratio is a measure of LGG-1 turnover (autophagic flux). (B-C) Western blot analysis of lysates from animals expressing GFP (lane 1), mKate2 (lane 2), GFP::LGG-1::mKate2 (lanes 3), and GFP::LGG-1::mKate2 in the *atg-4.1;atg-4.2* double mutant background (lane 4) probed with either anti-GFP (left) or anti-tRFP (right) antibodies. Arrowheads point to the full-length GFP::LGG-1::mKate2 protein band. (*) non-specific band, (#) cleavage product that is only present in the *atg-4.1;atg-4.2* double mutants. To avoid signal saturation, a smaller volume of the lysate from animals expressing GFP were loaded (B-C, lane 1). (D) Western blot analysis of lysates of non-transgenic parental worms (lane 1), and transgenic wild-type (lane 2), *atg-3(bp412)* mutants (lane 3), and *epg-5(tm3425)* mutants (lane 4) probed with an anti-LGG-1 antibody. Black arrow and arrowhead point to transgenic GFP::LGG-1 protein bands while red arrow and arrowhead points to endogenous LGG-1 protein bands.

To generate a reporter capable of high-throughput, high-content measurements of autophagic flux in live *C. elegans*, we engineered a reporter based on a concept previously reported [18] with some modifications. First, we fused a green fluorescent protein (GFP) and a red fluorescent protein, mKate2, to the N- and C-termini of LGG-1, respectively to generate GFP::LGG-1::mKate2 (Figure 1A). We did not include the C-terminal LGG-1ΔG (LC3ΔG) present in the Kaizuka reporter to avoid complications from possible recombination between the first and second LGG-1 during transgenesis and array formation [30]. mKate2 was chosen because it has a low propensity to aggregate compared to other red fluorescent proteins [31], and its excitation and emission properties allow simultaneous imaging with orange fluorescent protein-tagged reporters for studying the endolysosomal pathway previously described by our laboratory [32]. Second, the GFP::LGG-1::mKate2 transgene was placed under the control of the *nhx-2* promoter to drive expression specifically in the intestinal cells from the embryonic stage through adulthood [33]. Image-based high-content assays of live animals are generally lower resolution and require the use of lower magnification (e.g., 2.5x) objectives, which make them more suitable for assessing larger cell-types and tissues. We chose the intestine because it is one of the largest organs in *C. elegans* and its cells have advantageous properties for image-based high-content assays [34,35]. Although our study focuses on assessing autophagy in the intestine, these studies can be easily performed in other tissues like the muscle, hypoderm and neurons by driving expression of the AFR under the control of different tissue-specific promoters. Such studies should enable a more comprehensive assessment of autophagy in the context of a whole organism.

In *C. elegans*, the LGG-1 protein is synthesized as an autophagy inactive precursor molecule which is subsequently processed by the cysteine protease, ATG-4.1 (and to a lesser extent, ATG-4.2) at position 116 to expose a glycine residue at the C-terminus [13,36]. Based on work performed in yeast and mammalian systems, the cleaved product, LGG-1(G116), also referred to as LGG-1-I, likely undergoes further processing by ATG-7/Atg7 and ATG-3/Atg3 which ultimately leads to LGG-1 conjugation to PE [9,37,38]. LGG-1-PE (also known as LGG-1-II) participates in the expansion and elongation of isolation membranes and identification of select autophagic cargo through interaction with cargo receptors containing an LC3-interacting region (LIR) domain.

The GFP::LGG-1::mKate2 AFR was engineered to be translated as a single fusion protein. The fusion protein should be cleaved by endogenous ATG-4.1/-4.2 proteases at the C-terminus of LGG-1 to produce two individual proteins corresponding to GFP::LGG-1 and mKate2. To confirm that this was the case, we prepared lysates from animals expressing the GFP::LGG-1::mKate2 transgene and performed Western blot analysis (Figure 1B,C). As expected, a protein band migrating at ~27 kDa (the expected molecular mass of GFP) was detected in the lysate of animals expressing only GFP (Figure 1B, lane 1) but not in the animals expressing only mKate2 (Figure 1B, lane 2). Conversely, when the same samples were probed with the anti-tRFP antibody, a protein band migrating at ~26k Da, the expected molecular mass of mKate2 was detected in the lysate of animals expressing mKate2 (Figure 1C, lane 2) but not in the lysate of animals expressing GFP (Figure 1C, lane 1) demonstrating the specificity of each antibody. In WT animals expressing the GFP::LGG-1::mKate2 reporter, we detected ~ 42 kDa band, the predicted molecular mass of cleaved GFP::LGG-1 (Figure 1B, lane 3) and ~26 kDa band, the predicted molecular mass of free mKate2 (Figure 1C, lane 3) using the anti-GFP and anti-tRFP antibodies, respectively. A band corresponding to the uncleaved, full-length fusion protein (GFP::LGG-1::mKate2, ~68 kDa) was not detected in the wild-type background. However, this band appeared in the lysate from *atg-4.1(bp501);atg-4.2(tm3948*) double mutant animals (Figure 1B,C lanes 4, black arrowheads). From these results we concluded that the AFR was processed correctly into GFP::LGG-1 and mKate2 proteins by the endogenous ATG-4.1/ATG-4.2 proteases and that endogenous enzyme activity was sufficient to fully process the fusion protein.

Following cleavage by ATG-4 proteases, LGG-1 is conjugated to PE and anchored to the isolation membrane. PE-conjugated LGG-1 (LGG-1-PE or LGG-1-II) migrates at a faster rate than the non-lipidated form (LGG-1-I) on an SDS-PAGE gel [39]. To determine whether these species are detectable, we crossed the AFR transgenic animals with either *atg-3(bp412)* or *epg-5(tm3425)* mutants. ATG-3 is a E2-like enzyme that plays a critical role in conjugating PE to LGG-1 to form LGG-1-PE. Thus, in the *atg-3(bp412)* mutant background, the conjugation machinery is impaired, and levels of LGG-1-PE are expected to be significantly reduced or absent. Conversely, in the absence of EPG-5, a tethering protein important for the fusion between autophagosomes and lysosomes, levels of LGG-1-PE are expected to be increased due to impaired degradation of autophagosome contents. A protein band at ~45 kDa corresponding to the molecular mass of the non-lipidated GFP::LGG-1 (Figure 1D, lane 2, black arrow) was detected in the lysate from wild-type animals expressing the AFR transgene. Interestingly, we did not detect the faster migrating, lipidated form of GFP::LGG-1 under the conditions tested suggesting that baseline autophagy was not detectable in fed animals using this antibody. In contrast, the lipidated form of GFP::LGG-1 was detected in the lysate from *epg-5(tm3425)* mutants, where autophagosome-lysosome fusion was impaired (Figure 1D, lane 4, black arrowhead). In the lysate from *atg-3(bp412)* mutants, the lipidated GFP::LGG-1 band was not detected; instead, we detected a slower migrating GFP::LGG-1 band which was present in higher amount compared to that from wild-type and *epg-5(tm3425)* mutants (Figure 1D, lane 3, black arrow). The absence of the faster-migrating protein band in *atg-3(bp412)* mutants and the accumulation of the band in *epg-5(tm3425)* mutants are consistent with the faster-migrating band being the lipidated form of LGG-1. The differences in the levels of GFP::LGG-1 and GFP::LGG-1-PE were not due to differences in loading as similar total protein was loaded in each well (Fig. S1). We detected comparable band patterns with the endogenous LGG-1 indicating that the transgenic GFP-/mKate2-tagged LGG-1 was processed in a manner similar to the endogenous LGG-1 protein (Figure 1D, red arrow and arrowhead). Taken together these results indicated that the AFR is correctly processed and lipidated by the intestinal cells.

### Imaging of the AFR transgenic animals

**Figure 2.**
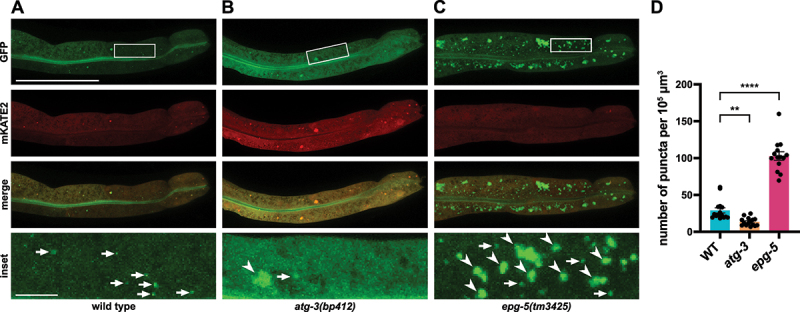
GFP::LGG-1 puncta number is decreased in *atg-3(bp412)* mutants but increased in size and number in *epg-5(tm3425)* mutants. Confocal images of the posterior intestine of (A) wild-type, (B) *atg-3(bp412)* mutants and (C) *epg-5(tm3425)* mutants expressing the AFR. Insets (lower panels) show higher magnification, single z-slice images of the boxed regions from (a-c). Arrows point to normal GFP::LGG-1 puncta and arrowheads point to large GFP::LGG-1 puncta that only accumulate in *epg-5(tm3425)* mutants. (D) Quantification of GFP::LGG-1 puncta number per intestinal cell. Error bars denote mean ± SEM. Animals were imaged as day 1 adults. Scale bar in (A) is 50 µm. Scale bar in inset is 10 µm.

We characterized the animals expressing the AFR transgene by confocal microscopy (Figure 2). If the GFP::LGG-1::mKate2 reporter was correctly processed, one would expect a diffuse cytosolic distribution of GFP and mKate2 in addition to punctate structures that are positive for GFP but not mKate2. Indeed, high-resolution imaging of animals showed diffuse GFP and mKate2 distribution primarily in the cytosol of the intestinal cells (Figure 2A). In addition, we detected small, GFP-positive puncta that were negative for mKate2 (Figure 2A, inset, white arrows). We also detected puncta that were positive for both GFP and mKate2. We speculate that these structures are likely uncleaved GFP::LGG-1::mKate2 fusion protein aggregates resulting from overexpression of the multicopy transgene. As such, we do not recommend using this reporter for autophagy assessment using puncta quantification.

ATG-3 participates in the conjugation of PE to cleaved LGG-1 and contributes to autophagosome formation. Thus, if the GFP-positive puncta are autophagosomes, their number should be reduced in *atg-3(bp412)* mutants. Indeed, GFP-positive puncta were significantly reduced in *atg-3(bp412)* mutants compared to those of wild-type animals expressing the AFR transgene (Figure 2B, D). The reduction in the number of GFP-puncta is consistent with the requirement of ATG-3/ATG3 for autophagosome formation [40-42]. Moreover, the presence of a small number of puncta in *atg-3(bp412)* mutants is consistent with *bp412* being a missense, hypomorphic allele as opposed to a null, which is lethal [43].

Following formation and expansion, autophagosomes fuse with lysosomes to produce autophagolysosomes. This fusion step is necessary for the digestion and recycling of autophagic cargo, and is facilitated by the interaction between EPG-5, RAB-7, SNAP-29, SYX-17, and VAMP-7/8 [44]. When transgenic animals expressing the AFR were crossed with *epg-5(tm3425)* mutants, we observed GFP-positive puncta that were larger and more numerous than those in the wild-type animals (Figure 2C, inset, arrowheads and arrows; and Figure 2D). The appearance of these GFP-positive puncta was similar to those previously reported for the GFP::LGG-1 reference strain crossed with *epg-5(tm3425)* mutants [39]. Puncta were absent in *epg-5(tm3425)* mutants expressing only GFP indicating that these structures were autophagosomes and rather than non-specific GFP aggregates (Fig. S2).

Together, the western blot and confocal imaging results demonstrated that the majority of the GFP::LGG-1::mKate2 reporter was properly cleaved into GFP::LGG-1 and mKate2 by endogenous ATG-4.1/-4.2 and that the cleaved GFP::LGG-1 was lipidated and packaged into autophagosomes while mKate2 localized to the cytosol. Moreover, the changes in GFP::LGG-1 puncta in the autophagy mutant animals were as predicted and consistent with previous studies [39].

### Effect of GFP::LGG-1::mKate2 expression on organismal fitness

The AFR transgene was integrated into the *C. elegans* genome by X-ray irradiation [34]. Although the integrants were backcrossed with the wild-type parental (N2) strain six times, it is important to assess whether the integration itself or the transgene expression had any unwanted effects on the animal. To test this, we compared brood size, growth rate through post-embryonic development, and lifespan of the wild-type parental strain, the reference GFP::LGG-1 reporter strain [25,26], and the transgenic AFR strain (Fig. S3). The brood size (Fig. S3A) and lifespan (Fig. S3B and Table S3) of transgenic animals expressing the AFR were not statistically different to those of the wild-type parental strain. The growth rate of the animals expressing the AFR and the reference GFP::LGG-1 reporters was marginally slower than that of the control strain (Fig. S3C). These differences, although statistically significant, are likely not biologically significant. Therefore, we concluded that the expression of the AFR transgene had no major impact on adult fitness and minimal impact on larval development.

### Automated, image-based quantification of autophagic flux in live animals

**Figure 3.**
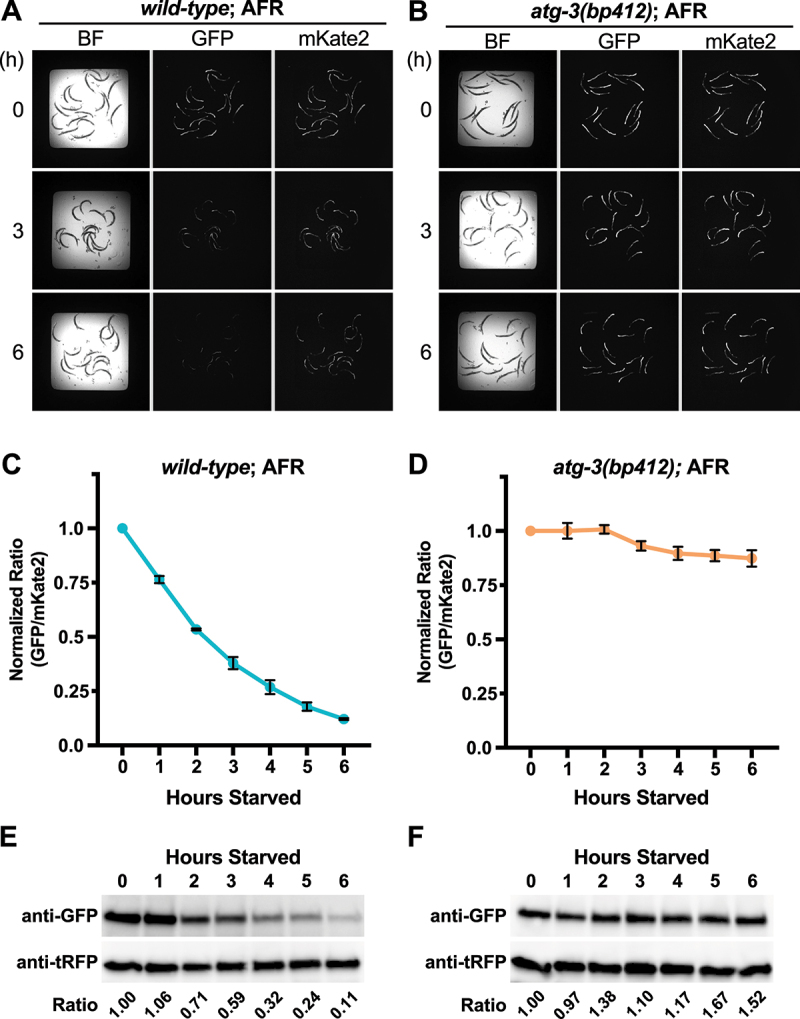
The AFR is sensitive to nutrient deprivation. Starvation induces, a time-dependent decrease in GFP:mKate2 ratio of (A) wild-type animals but not (C) *atg-3(bp412)* mutants. Each point represents an average of three experiments (~75 animals). Error bars denote mean ± SEM. Western blots of lysates from (B) wild-type or (D) *atg-3(bp412)* mutants. Each lane contains lysates from exactly 50 day 1 adult animals. Upper panels were probed with an anti-GFP antibody while the lower panels were probed with an anti-tRFP antibody.

Since the GFP::LGG-1 and mKate2 proteins are synthesized at an equimolar ratio, the ratio of GFP:mKate2 fluorescence is modulated relative to the autophagic flux state of the cell. For example, upon nutrient deprivation or starvation, the turnover of GFP::LGG-1 increases as more GFP::LGG-1 is recruited into autophagosomes and degraded upon fusion with lysosomes. In contrast, the mKate2 protein in the cytosol should be degraded at a slower rate. Based on these assumptions, cells of animals cultured in the absence of food should have a higher autophagic flux (i.e., lower GFP:mKate2 ratio) compared to those of animals cultured in the presence of food. Conversely, if autophagic flux is impaired (e.g., via genetic mutations that limit autophagosome-lysosome fusion), the GFP:mKate2 ratio would be higher as less GFP::LGG-1 will be sequestered and degraded by autolysosomes. To test whether these predicted changes in GFP:mKate2 ratio occurred, we cultured animals in the presence or absence of food and quantified their GFP and mKate2 fluorescence levels using a plate reader type, high-content imager (Thermofisher CellInsight CX-7) (Figure 3). For ease of comparison, the GFP:mKate2 ratio at time 0 was normalized to 1.0. The GFP:mKate2 ratio decreased significantly within the first hour of starvation and continued to decrease steadily over the next 5 hours, reaching a ratio of ~0.2 after 6 hours (Figure 3A,C) P:mKate2 ratio could be due to a decrease in GFP::LGG-1 fluorescence or an increase in mKate2 fluorescence. We confirmed that the decreased GFP:mKate2 ratio upon starvation was due to a steady decrease in GFP::LGG-1 fluorescence and protein levels (Figure 3E, upper panel, and Fig. S4A). mKate2 fluorescence and protein levels also decreased over the 6-hour starvation period, albeit at a much slower rate than GFP::LGG-1 (Figure 3E, lower panel, and Fig. S4B).

To confirm that enhanced autophagy was responsible for the decrease in the GFP:mKate2 ratio, we repeated the 6-hour starvation experiment in the *atg-3(bp412)* mutant background. In contrast to wild-type animals, the starvation-induced decrease in GFP:mKate2 ratio was significantly attenuated in *atg-3(bp412)* mutants (Figure 3B,D,F).

**Figure 4.**
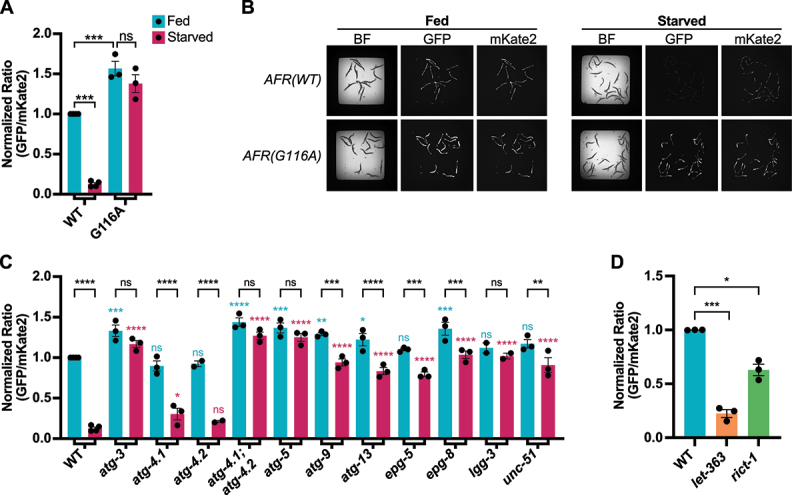
GFP:mKate2 ratio is modulated in autophagy mutants. GFP:mKate2 ratios (A) and representative well images (B) of animals expressing the wild-type AFR or AFR(G116A) mutant reporter. Fed animals are shown in blue. Animals starved for 6 hours are shown in red. GFP:mKate2 ratios of the AFR in (C) various autophagy mutants, (D) *let-363(ok3018)* mutant and *rict-1(ft7)* mutant backgrounds. All data are from day 1 adults and were normalized to the fed wild-typed control. All animals carried the AFR transgene. Data are an average of three independent experiments except for *lgg-3* and *atg-4.2* mutants which are an average of two independent experiments. For *let-363(ok3018)* which are homozygous lethal at the L3 stage, data were normalized to the L3 stage wild-type control. Error bars denote mean ± SEM.

We also determined whether mutations in other autophagy genes altered GFP:mKate2 ratio in the presence or absence of food. As a control, we generated a GFP::LGG-1(G116A)::mKate2 mutant reporter (heretofore referred to as AFR(G116A)) where the glycine at position 116 was substituted with an alanine (Fig. S5). The G116A substitution prevents the GFP::LGG-1 from being cleaved from mKate2 by the ATG-4.1/-4.2 proteases [11,13]. Consequently, the AFR(G116A) serves as a non-cleavable autophagy substrate control. Under fed conditions, the GFP:mKate2 ratio of day 1 (D1) adult animals expressing the AFR transgene was ~1.0, representing the low basal rate of autophagy in wild-type animals (Figure 4A,B). In contrast, the GFP:mKate2 ratio of animals expressing the AFR(G116A) was ~1.5 (Figure 4A,B). This ratio represents the maximum theoretical ratio obtainable when canonical autophagy is completely blocked. Therefore, the difference in the ratios of AFR and AFR(G116A) represents the relative magnitude of basal autophagic flux in wild-type animals under fed conditions.

When exposed to starvation conditions for 6 hours, the GFP:mKate2 ratio of animals expressing the AFR significantly decreased to ~0.2, indicating an enhanced rate of autophagic flux (Figure 4A,B). In contrast, the GFP:mKate2 ratio of animals expressing the AFR(G116A) mutant reporter remained near 1.5, confirming that cleavage by ATG-4.1/-4.2 was necessary for the autophagy reporter to function (Figure 4A,B).

We crossed the AFR transgene into known autophagy mutants and measured their GFP:mKate2 ratios (Figure 4C and Fig. S6). Under fed conditions, most of the autophagy mutants had higher GFP:mKate2 ratios (ranging from 1.2 to 1.4) compared to wild-type animals expressing the AFR, consistent with there being less GFP::LGG-1 turnover (i.e., lower autophagic flux) in the autophagy mutants (Figure 4C, blue bars). Upon starvation for 6 hours, the GFP:mKate2 ratios in most of the autophagy mutants remained above 1.0 (Figure 4C, red bars). These results indicated that the changes in the GFP:mKate2 ratio of the AFR were sensitive to the nutritional status of the animal and were dependent on the activity of these core autophagy genes. Of note, unlike other autophagy mutants tested, starvation-induced autophagy was only slightly attenuated in *atg-4.1* and *atg-4.2* single mutants but was significantly attenuated in *atg-4.1; atg-4.2* double mutants (Figure 4C). These results are consistent with ATG-4.1 and ATG-4.2 having redundant functions in autophagy and suppression of starvation-induced autophagy required the inactivation of both genes.

Genetic perturbations in known negative regulators of autophagy increase autophagic flux. *let-363* is the *C. elegans* ortholog of mTOR (mechanistic target of rapamycin) which forms the enzymatic core of the highly conserved mTOR complexes, mTORC1 and mTORC2 [45]. *rict-1* is an ortholog of Rictor which is primarily thought to associate with mTORC2 [45]. Both *let-363* and *rict-1* mutants are important in regulating autophagy in *C. elegans* [12,46]. To determine whether the AFR could detect a decrease in mTORC1/2 activity, we crossed the AFR transgene into *let-363(ok3018) and rict-1(ft7)* mutant animals and measured their GFP:mKate2 fluorescence ratios. To be consistent with previous studies, we measured autophagic flux of *rict-1(ft7)* animals as day 1 adults. However, because *let-363(ok3018)* mutants are homozygous lethal and arrest at the late L3 larval stage [47,48] we measured autophagic flux of L3 larvae to avoid pleotropic effects of older and sicker animals. Under fed conditions, the GFP:mKate2 ratios were significantly lower in *let-363(ok3018)* and *rict-1(ft7)* animals as compared to wild-type animals expressing the AFR of the same age (Figure 4D and Figure. S7). Together these results demonstrated that the AFR was capable of rapid, real-time detection of basal, impaired, and enhanced autophagic flux in live animals.

### Autophagic flux was enhanced in wild-type adults and in daf-2 animals during post-embryonic development

**Figure 5.**
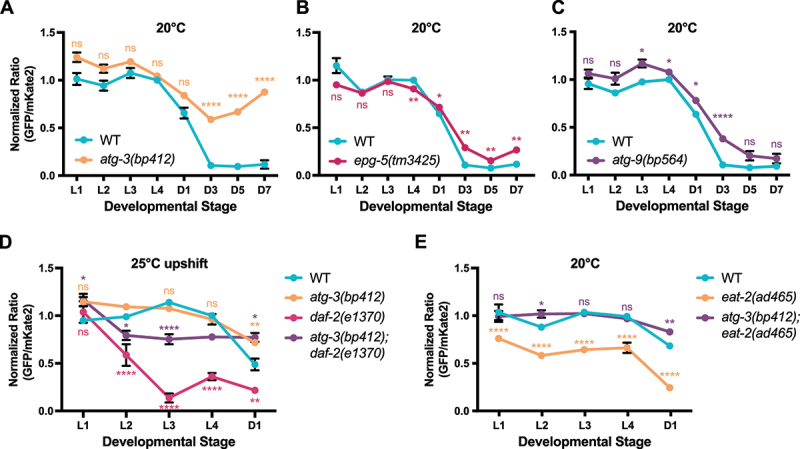
Autophagy is differentially regulated during post-embryonic development and aging. Autophagic flux profiles of (A) wild-type and *atg-3(bp412)* mutants measured from L1 larvae to D7 adults. Autophagic flux profiles of (B) *epg-5(tm3425)* and (C) *atg-9(bp564)* mutants. Autophagic flux profiles of (D) *daf-2(e1370)* mutants and controls upshifted to 25°C for 24 hours. Autophagic flux profiles of (E) *eat-2(ad465)* mutants. Data were normalized to wild-type L4 stage animals for comparison. Data are an average of three independent experiments. All animals carried the AFR transgene. Error bars denote mean ± SEM.

Basal levels of autophagy play important roles in multiple biological processes during embryonic development. In *C. elegans*, maternally-derived P-granules and sperm-derived paternal mitochondria are selectively degraded by autophagy [24,39,49,50]. Autophagy is also required for dauer development and lifespan extension in *C. elegans* [26,46,49,51-53]. However, changes in autophagic flux during post-embryonic development have not been well-investigated due to the difficulty in monitoring autophagy accurately and longitudinally in real time. Therefore, we utilized the AFR probe in a high-content assay to rapidly measure autophagic flux during different stages of post-embryonic development and aging. Age-synchronized L1-, L2-, L3-, and L4-staged larvae, day 1 (D1), day 3 (D3), day 5 (D5), and day 7 (D7) adult animals were placed into 384-well plates, and images were automatically acquired and quantified using the high-content imager (Figure 5A and Fig. S8). The GFP:mKate2 ratio of the wild-type L1, L2, L3 and L4 larvae were similar (between 0.9 and 1.1). Interestingly, these ratios were only slightly lower, although not statistically different, than those of the *atg-3*(*bp412*) hypomorphic mutants (Figure 5A, orange) suggesting that basal autophagic flux may be relatively low during wild-type larval development. In contrast, the GFP:mKate2 ratio decreased significantly in D1 and D3 adults. The decrease in GFP:mKate2 ratio (i.e., a marked increase in autophagic flux) in the intestine of animals from L4 larvae to D3 adults may be due to the increase in need to mobilize nutrients for the developing embryo during the reproductive stages. We speculate that the increase in autophagic flux in the intestine of animals from L4 to D3 adults may be due to the increase in need to mobilize nutrients for the developing embryo during the reproductive stages. The GFP fluorescence signals in D5 and D7 wild type adults were also low due to a decrease in the *nhx-2* promoter activity, however, the GFP signal was higher than the autofluorescence of the non-transgenic, parental (VC2010) line. In contrast, GFP fluorescence was significantly higher in the *atg-3* mutants where autophagy was impaired. Like the wild-type animals, the GFP:mKate2 ratio of *atg-3* mutants was the lowest in D3 adults, but gradually increased in D5 and D7 adults (Figure 5A, orange). These results are consistent with autophagic flux being the highest at D3 before gradually decreasing in older mutant animals.

To determine whether a similar pattern would be observed in other autophagy mutants, we measured GFP:mKate2 ratios in *epg-5(tm3425)* (Figure 5B) and *atg-9(bp564)* (Figure 5C) mutant animals. The GFP:mKate2 ratios in *epg-5(tm3425)* mutants followed a similar trend to that of the *atg-3(bp412)* mutants albeit to a lesser extent. Compared to wild-type animals, the GFP:mKate2 ratios of *epg-5(tm3425)* mutants were not statistically different during early larval (L1-L3) development but were statistically higher in adults (Figure 5B, red). In *atg-9(bp564)* mutant animals, the GFP:mKate2 ratios were higher than in respective wild-type animals at L3 – D3 stages but not at D5 and D7 (Figure 5C, purple). These results suggest that GFP:mKate2 ratio and hence autophagic flux is differentially impaired in different autophagy mutants underscoring the importance of evaluating multiple autophagy mutants. Furthermore, different autophagy genes may be more or less important for autophagic function during different stages of development and aging. Since *atg-3(bp412)* mutants showed the strongest block in autophagic flux, we chose this mutant as the preferred control for subsequent experiments assessing the role of autophagy in other genetic backgrounds.

Autophagy is elevated in the long-lived, *daf-2* (insulin/insulin-like signaling) mutants [26,29,52]. Higher numbers of GFP::LGG-1 puncta were reported in the hypodermal seam cells of *daf-2* dauer larvae compared to wild-type L3 larvae [26]. However, autophagy during other stages of post-embryonic development has not been extensively studied. Thus, we assessed the feasibility of measuring autophagic flux in *daf-2* mutant animals during different stages of post-embryonic development using the AFR and high-content imaging. Because *daf-2(e1370)* mutant animals are temperature-sensitive and form dauers constitutively at 25°C, we maintained animals at the permissive temperature (15°C) until just prior to the experiment. At 15°C, the GFP:mKate2 ratios of *daf-2(e1370)* mutant animals were similar to those of wild-type animals (Fig. S9A). To investigate the effects of the *daf-2* mutation, we transferred a mixed age population of animals to 25°C (restrictive temperature). We also picked L4 stage animals and transferred them to 25°C. After 24 hours at the restrictive temperature, L1, L2, L3, L4 larvae and D1 adults were picked for image capture and analysis. Consistent with the *daf-2(e1370)* mutation being temperature-sensitive, the GFP:mKate2 ratios of *daf-2(e1370)* mutant animals placed at 25°C for 24 hours were markedly different to those of wild-type animals expressing the AFR cultured under the same conditions (Figure 5D). While the GFP:mKate2 ratios of L1 animals were similar, we detected a significant decrease in the GFP:mKate2 ratio in L2, L3, L4 and D1 *daf-2(e1370)* mutant animals (Figure 5D, red) with the lowest ratio observed at the L3 larval stage. Autophagic flux was significantly attenuated in *daf-2(e1370); atg-3(bp412)* double mutants throughout larval development compared to that of *daf-2(e1370)* single mutants (Figure 5D, purple). Taken together these findings confirm that autophagic flux is increased in *daf-2(e1370)* mutant animals consistent with previous work [29].

We were not able to accurately measure the GFP:mKate2 ratio of *daf-2(e1370)* dauer animals on the high-content imager because the *nhx-2* promoter used to drive the transgene was significantly down-regulated in dauers (data not shown). Nonetheless, using confocal microscopy, we confirmed enhanced autophagic flux in dauer animals as determined by a GFP:mKate2 ratio of ~0.3 (Fig. S9B and C).

Mutations in *eat* genes (e.g., *eat-2*) are known to impair pharyngeal pumping, reduce caloric intake and extend lifespan [46,54–56]. In *eat-2* mutants, the FOXA transcription factor, PHA-4, is required for both lifespan extension and autophagy, however, in *daf-2* mutants, the FOXO transcription factor, DAF-16, is required for lifespan extension but not autophagy [46]. We, therefore, hypothesized that *daf-2* and *eat-2* mutants would have distinct autophagic flux profiles during post-embryonic development. Indeed, the GFP:mKate2 ratios of *daf-2* and *eat-2* mutants were different. At the L1 larval stage, the GFP:mKate2 ratio of *daf-2(e1370)* animals was close to 1.0 similar to that of the wild-type animals expressing the AFR. However, the GFP:mKate2 ratio decreased significantly at the L2 stage and decreased further at the L3 stage (Figure 5D, red), suggesting that autophagic flux was enhanced in *daf-2(e1370)* animals starting from the L2 larval stage. In *eat-2* mutants, the GFP:mKate2 ratio was modestly decreased at all stages of post-embryonic development (Figure 5E, orange). Although we were not able to measure pharyngeal pumping in L1 and L2 larvae, we were able to confirm that L3, L4 and D1 *eat-2* mutants expressing the AFR indeed have significantly reduced pharyngeal pumping rates compared to their respective wild-type controls (Fig. S10). Moreover, the lower GFP:mKate2 ratio in *eat-2* mutants was completely restored to wild-type levels in *eat-2;atg-3* double mutants (Figure 5E, purple) suggesting that *eat-2* mutants have enhanced autophagic flux resulting from reduced pharyngeal pumping and caloric restriction. These studies suggest that autophagic flux is differentially regulated in different long-lived mutants.

### A high-content small molecule screen identifies autophagy inducing compounds

High-throughput, high-content screens aimed at identifying autophagy modulating drugs have been performed using mammalian cells [57, 58, 59, 60]. However, to our knowledge, screens specifically aimed at identifying autophagy modulating compounds in live animals have not been performed. Therefore, we determined whether the AFR could be used in a high-throughput, high-content screening platform to identify autophagy altering drugs active in *C. elegans.*

**Figure 6.**
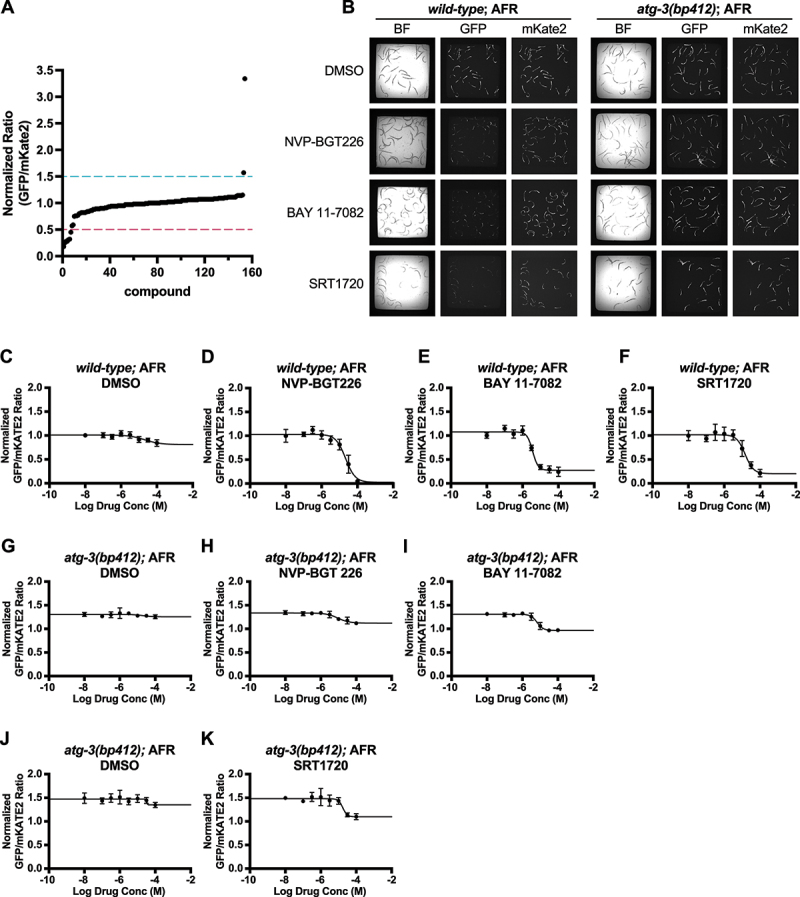
A high-content small molecule screen identifies autophagy modulating compounds. (A) GFP:mKate2 ratio of 154 compounds from the Autophagy compounds library (Selleckchem). Each compound was used at a final concentration of 25 µM. There were approximately 30 L4 stage animals per well. Treatment time was 24 hours. Ratios were normalized to the DMSO control. Dots above the blue dotted line represents compounds that increased the GFP:mKate2 ratio above 1.5. Dots below the red dotted line represents compounds that decreased the GFP:mKate2 ratio below 0.5. Representative well images are shown in (B). A complete list of compound names, descriptions and corresponding GFP:mKate2 ratios can be found in Table S2. Eight-point dose response curves of wild-type;AFR animals treated with (C) DMSO, (D) NVP-BGT226, (E) BAY 11-7082, and (F) SRT1720. Eight-point dose response curves of *atg-3(bp412)*;AFR animals treated with (G) DMSO, (H) NVP-BGT226, (I) BAY 11-7082, (J) DMSO, and (K) SRT1720. The SRT1720 drug dose-response experiments on *atg-3* mutant animals (J-K) were performed on different dates than the other experiments. As such, we included the DMSO control performed on the same dates for comparison. Graphs are representative of three independent experiments. Error bars denote mean ± SEM. All animals carried the AFR transgene.

As an initial screen, we tested the Autophagy Compounds Library (Selleck Chemicals LCC), a unique collection of 154 small molecules reported in the literature to have autophagy modulating activity mostly in mammalian cells. Approximately 20 L4-stage animals expressing the AFR were placed into each well of a 384-well plate containing a unique small molecule at a final concentration of 25 µM using a modification of the protocol previously reported by our laboratory [34,35]. Animals were incubated in the drug solution for 20-24 hours prior to measuring the GFP/mKate2 fluorescence using the CX7 high-content imager. A complete list of all the compounds in the library and their respective GFP:mKate2 ratios are shown in Table S2. Of the compounds tested, we identified two that appeared to decrease autophagic flux (i.e., increased the GFP:mKate2 ratio above 1.5) and seven that enhanced autophagic flux (i.e., reduced GFP:mKate2 ratio below 0.5) (Figure 6A). Both compounds that increased the GFP:mKate2 ratio (MC1568 and sirtinol) were false positives that displayed significant autofluorescence in the green channel. Of the seven that significantly reduced the GFP:mKate2 ratio, two (obatoclax mesylate and doxorubicin) were also eliminated because of compound autofluorescence. Two of the hits, tamoxifen and trifluoperazine (a member of the phenothiazine family), were identified previously in *C. elegans* screens for drugs that enhanced autophagy and decreased the accumulation of α1-antitrypsin Z (ATZ) [34,61]. Therefore, we focused our efforts on characterizing the three remaining small molecules, NVP-BGT226, BAY 11-7082 and SRT1720. NVP-BGT226 is a novel class I dual PI3K/mTOR inhibitor; BAY 11-7082 is an inhibitor of nuclear factor-κB, USP7, USP21 and gasdermin D; and SRT1720 is a selective activator of SIRT1. All three of these compounds robustly enhanced autophagic flux as shown by a decrease in the GFP:mKate2 ratio in a dose-dependent manner (Figure 6B–F). These effects were blocked in atg-3 mutants (Figure 6G–K) confirming that the hit compounds act in an autophagy-dependent fashion. NVP-BGT226 and SRT1720, and to a lesser extent BAY 11-7082 also reduced the accumulation of GFP::ATZ, a mutant protein that has previously been shown to be degraded by autophagy (Fig. S11 and [34,35]).

To confirm that the hit compounds were bona fide autophagy activators, we tested for compound effects on food quantity (bacteriostatic or bactericidal activity) and intake (pharyngeal pumping rates), both of which could reduce caloric consumption and potentially activate starvation-induced autophagy. All three hit compounds modestly reduced (~6-9%) the pumping rates compared to the dimethylsulfoxide (DMSO) treated animals (Fig. S12A). This modest reduction in the pumping rates is unlikely to be responsible for the marked increase in autophagic flux following drug treatment (Figure 6). We next tested for compound effects on the optical density (OD) of *E. coli* OP50 (laboratory food for *C. elegans*). DMSO, NVP-BGT226 and SRT1720 did not have a significant effect on bacterial OD at the concentrations tested (Fig. S12B), however, BAY 11-7082 at the highest three doses significantly decreased bacterial OD by ~50% (Fig. S12B, orange). Thus, we cannot preclude that BAY 11-7082ʹs autophagy enhancing activity was due to its effect on bacterial growth leading to starvation-induced autophagy.

Taken together these results suggest that NVP-BGT226 and SRT1720 are potentially useful tool compounds for investigating the autophagy pathway in *C. elegans*.

## Discussion

We validated a fluorescent reporter for rapid quantification of autophagic flux in live *C. elegans*. Autophagic flux was assessed by measuring the relative cellular GFP::LGG-1 and mKate2 fluorescence levels using a microscope fitted with a low magnification (2.5x) objective and a digital camera. This approach eliminates the need for high-resolution microscopy and GFP::LGG-1 puncta quantification which are time-consuming and labor-intensive. The ability to efficiently measure autophagic flux in one step also obviates the need for secondary studies to determine whether an increase in LGG-1 puncta is due to enhanced autophagy or impaired autophagic flux. Moreover, by combining the AFR with a high-content imager, high-throughput quantitative assessment of autophagic flux can be followed in response to genetic or pharmacological manipulation.

A key advantage of this AFR is that changes in cellular autophagic flux can be quantitatively assessed during all postembryonic stages, from L1 larvae to D3 adults, making it possible to perform longitudinal autophagy studies. We found that basal autophagic flux was relatively low during the four larval stages but increased significantly during the adult reproductive period. Although it was difficult to accurately assess autophagic flux in older wild type adults due to increased gut autofluorescence and reduced AFR expression, we were able to detect changes in GFP:mKate2 in *atg-3(bp412)* mutants. Because *bp412* is a hypomorphic allele, autophagic flux is not completely blocked in these animals. The GFP:mKate2 ratio in *atg-3(bp412)* mutants was the lowest in D3 adults similar to wild-type adults, however, the ratio increased steadily in D5 and D7 adults, consistent with there being a strong requirement for autophagy in early adulthood [62] and a general decline in autophagy as the animals aged [29]. We also observed distinct temporal regulation of autophagic flux in different genetic backgrounds (wild-type, *daf-2*, and *eat-2*). While autophagic flux was relatively low during wild type larval development, it was markedly enhanced in *daf-2(e1370)* animals at the L2, L3, and L4 larval stages and in D1 adults. In *eat-2* mutants, autophagic flux was modestly elevated throughout postembryonic development. However, unlike *daf-2(e1370)* mutants, temporal regulation of autophagy was similar to that of wild-type animals. These observations suggest that *eat-2* mutants have elevated autophagy secondary to caloric restriction [46,54] rather than a dramatic reprogramming of the autophagy pathway.

Our study also highlights the importance of studying autophagic flux in all developmental stages. For example, we observed minor differences in GFP:mKate2 ratio between wild-type and *atg-3(bp412)* mutants at the L3 and L4 larval stages but observed major differences in autophagic flux between D3 and D7 adults. Similarly, the differences in GFP:mKate2 ratios between wild-type and *daf-2* mutants were modest at the L1 larval and D1 adult stages but were most pronounced at the L2, L3, and dauer larval stages consistent with autophagy being required for remodeling and entry into the dauer diapause [26,46]. Likewise, for homozygous *let-363/mTOR* mutants, elevated autophagic flux was observed in the L3 larval stage but not in the L1 or L2 larval stages (Fig. S13). This is likely because there is a large maternal load of LET-363 protein that is depleted at the L3 larval stage. Our findings emphasize the dynamic nature of autophagy and underscores the need to cautiously interpret results obtained at a single developmental stage or time point. Instead, a comprehensive assessment across all developmental stages is crucial for a more accurate understanding of how autophagy is regulated. The simplicity of the AFR should facilitate these types of longitudinal autophagy studies.

*C. elegans* has proved useful in identifying novel autophagy genes that are absent in yeast, but are present in metazoans [24]. The ability to rapidly assess autophagic flux in live animals makes it possible to employ high-throughput procedures for conducting forward and reverse genetic screens. Our study suggests that the GFP:mKate2 ratio alone is a good measure of autophagic flux. In addition to the ratio, the presence or absence of GFP-positive puncta can provide additional information about where a gene of interest is acting in the autophagy pathway. For example, when the GFP:mKate2 ratio is relatively high and GFP puncta are absent, autophagic flux is likely blocked before the complete formation of autophagosomes or the conjugation of PE to LGG-1, as in the case of the *atg-3* mutant (Figure 2). Conversely, when the GFP:mKate2 ratio is high and large GFP puncta are present, autophagic flux is likely blocked after the complete formation of autophagosomes and before fusion with lysosomes as observed in the *epg-5* mutant (Figure 2). These phenotypes may be further exploited to identify new genes involved in regulating different stages of the autophagy pathway.

Interestingly, starvation-induced autophagy was not completely blocked in all the mutants tested. In some cases, e.g., *atg-3(bp412)*, this was due to the mutant being a hypomorph rather than a null. However, in other cases, we observed a significant reduction in GFP:mKate2 ratio in presumed null mutants *atg-9(bp564), atg-13(bp414), epg-5(tm3425), epg-8(bp251)* and *unc-51(e1189)* upon starvation for 6 hours. These results support the presence of alternative GFP::LGG-1 clearance mechanisms that act in parallel to the canonical autophagy pathway. Genetic screens for enhancers using AFR-expressing autophagy mutants may help identify genetic mechanisms underlying these alternative pathways.

The utility of the AFR was also demonstrated by developing a high-content screening assay for compounds that modulate autophagy in *C. elegans*. Two drugs that we previously identified in screens that decreased accumulation of aggregation-prone ATZ protein by enhancing autophagy [34,61] were confirmed in this screen. We further identified three compounds from a small chemical library that efficiently reduced the GFP:mKate2 ratio in a dose-dependent manner. Although all three compounds enhanced autophagy, BAY 11-7082 may induce autophagy indirectly by adversely affecting bacterial growth and inducing starvation. These findings demonstrate the importance of carefully characterizing potential autophagy-enhancing compounds. At the very minimum, we recommend that hit compounds be tested in secondary assays to confirm that they do not have significant adverse effects on pharyngeal pumping and bacterial growth.

Most of the 154 compounds annotated as autophagy modulators in mammalian cells were not active in *C. elegans* at the concentration (25 µM) tested. This discrepancy may be secondary to a decrease in bioavailability in the *C. elegans* intestine [63]. Also, *C. elegans* employ multiple xenobiotic detoxification and efflux systems to eliminate the potentially noxious compounds from the cells [64,65]. To achieve optimal drug effect in *C. elegans*, drugs like bafilomycin A are often injected directly into the cells or the pseudocoelomic space [29]. Higher hit rates could be achieved by using higher drug concentrations, or by using mutant strains that are reported to be hypersensitive to drugs such as *srf, bus* or *pgp* mutants [66–69]. Alternatively, the amino acid sequence of the drug targets may be sufficiently diverged in *C. elegans* such that the drugs fail to bind to the nematode proteins. This is not surprising since drug-target interactions are highly specific and a single amino acid substitution in the target protein can substantially impact drug binding and activity [70,71]. A third possibility is that these drugs may act on tissue-specific proteins or pathways that are not expressed in the *C. elegans* intestine.

The AFR described in this report was designed to measure autophagic flux in a single tissue: the *C. elegans* intestine. Because autophagy is regulated in a tissue-specific manner, a pan-cellular AFR may be difficult to interpret and suboptimal for a high-throughput, high-content evaluation of autophagy. For example, under anoxic conditions, autophagy was significantly upregulated in the intestine but not in the hypodermis, pharynx, muscle or neurons of *C. elegans* [17]. Similarly, in aging adults, the steady state autophagosome and autolysosome pool sizes generally increased with age in different tissues with different trajectories, however, the autolysosome pool size decreased in neurons [29]. Therefore, a more practical approach may be to generate additional tissue-specific AFRs by placing the transgene under the control of promoters specific for the muscle (e.g., *myo-3*), hypoderm (e.g., *col-19*) and neurons (e.g., *rab-3*). In addition to being useful tools for understanding the spatiotemporal regulation of autophagy, these tissue-specific AFRs may also shed light on how different cells/tissues communicate with each other to orchestrate organismal response to stimulatory or inhibitory autophagy cues.

In summary, we have developed and characterized an AFR for efficient quantification of cellular autophagic flux in live *C. elegans*. By coupling the AFR with a high-content imager, autophagic flux was assessed rapidly by measuring GFP/mKate2 fluorescence ratios. This approach overcomes the need for time-consuming, high-magnification, high-resolution microscopic imaging of individual cells followed by manual quantification of LGG-1 puncta. Moreover, the AFR allows assessment of basal, induced, and impaired canonical autophagic flux in all stages of post-embryonic development and adulthood and can be easily adapted for high-throughput applications such as drug screening, and forward/reverse genetic screens to identify genetic modulators of autophagy.

## Materials and methods

### Caenorhabditis elegans strains and culture conditions

Animals were routinely cultured at 20°C on nematode growth medium (NGM) plates seeded with *E. coli* strain, OP50 as previously described [72] unless otherwise specified in the text. A full list of strains used in this study is in Table S1. *daf-2(e1370)* animals are temperature sensitive and form constitutive dauers. As such, they were routinely maintained at 15°C. Homozygous *let-363* animals arrest late during larval stage L3 and so were maintained as heterozygotes. To propagate *let-363(ok3018)* animals, a heterozygous balanced line (VC2312), in which the rearrangement hT2 for chromosome I that suppresses crossovers is marked with a pharyngeal GFP marker was used. To generate sufficient homozygous L3 animals for experimentation, 15 heterozygous *let-363*/hT2 I; AFR L4 larval stage animals positive for pharyngeal GFP were passaged to three 100mm NGM plates. From the progeny of these heterozygous animals, larval stage L3 animals negative for pharyngeal GFP, and therefore homozygous for *let-363*, were picked for our studies.

### Autophagic flux reporter (AFR)

The *gfp::lgg-1::mKate2* transgene was synthesized as a gene block by Genscript and cloned into the Xho I and Sac I restriction enzyme sites of pPD49.23 under the control of the *nhx-2* promoter to generate *nhx-2p::gfp::lgg-1::mKate2* autophagic flux reporter (AFR). Site-directed mutagenesis was then used to generate the AFR(G116A) mutant control. Maps and full sequences of the AFR constructs are shown in Table S4. Transgenic animals were generated by injecting the plasmid into the gonad of young adult hermaphrodites at a final concentration 30 ng/µl along with 50 ng/µl pBluescript. Extrachromosomal arrays were then integrated using x-radiation as previously described [34]. Stable integrants were backcrossed to the N2 strain 6 times to minimize background mutations caused by x-radiation.

### Immunoblotting

For denaturing conditions, 100 L4 stage worms were picked onto 100 mm NGM plate. 24 hours later, 100 day 1 (D1) adults were picked into 25µl 1x phosphate-buffered saline (PBS). Upon adding 25µl 2x SDS loading buffer (10% SDS, 8M Urea, 1:20 β-mercaptoethanol), worms were flash frozen in liquid nitrogen then boiled at 100°C for 10 minutes. 20µl of lysate was loaded onto a 4-12% Criterion Tris-Glycine TGX mini-precast gel (Bio-Rad, Hercules, CA, USA) or 40µl onto a 12% Criterion Bis-Tris TGX midi-precast gel (Bio-Rad, Hercules, CA, USA) and resolved under reducing conditions. Protein bands were then transferred to a polyvinylidene difluoride (PVDF) membrane using the Trans-Blot Turbo Transfer or Criterion (Bio-Rad) western blotting systems. Membranes were blocked with blocking buffer (5% skim milk in tris-buffered saline, 0.1% Tween-20 (TBST)) at room temperature for 1 hour and then incubated with primary antibodies overnight at 4°C. For detection of GFP::LGG-1 bands we used a rabbit anti-GFP monoclonal antibody (Cell Signaling Technology, 2956S) at 1:1000, followed by an anti-rabbit HRP antibody (Cell Signaling Technology, 7074S) at 1:2000. For detection of mKate2 bands, we used a rabbit anti-tRFP polyclonal antibody (Evrogen, AB233) at 1:1000, followed by an anti-rabbit HRP antibody (Cell Signaling Technology, 7074S) at 1:2000. All antibodies were diluted using the blocking buffer. HRP-reactive bands were visualized using Clarity™ Western ECL substrate (Bio-Rad) on a Bio-Rad Chemidoc system.

### Detection of LGG-1-I and LGG-1-II bands

For resolving LGG-1-I and lipidated LGG-1-II bands, a mixed population of worms from two 100 mm NGM plates were washed off using 1x PBS with 0.01% pluronic acid and transferred into a 15ml conical tube. Worms were pelleted by centrifugation at 720 *g* using an Eppendorf swing bucket 5804R tabletop centrifuge. The pellet was washed three times with 1x PBS (+0.01% pluronic) then resuspended in 1ml of ice-cold RIPA buffer and transferred to a 1.7ml microfuge tube and centrifuged on a benchtop centrifuge at 6200*g* for 30s on a benchtop microfuge. The supernatant was discarded, and the pellet was resuspended with 100µl of RIPA buffer with HALT protease inhibitor (Thermofisher, 78444). The worm pellet was flash frozen in liquid nitrogen and thawed five times. The pellet was then lysed by using a Fisherbrand Model FB120 Sonic Dismembrator with CL-18 Ultrasonic Probe Converter for 2 mins (pulse 30s at 70% power followed by 30s rest). The sample was then incubated on ice for 30 mins and centrifuged at 13800 *g* at 4°C. 20µl of clarified lysate was then added to 20µl of 2X Laemmli sample buffer (Bio-Rad, 1610737) with β-mercaptoethanol (5% final concentration), and incubated for 8 mins at 95°C, cooled to room temperature, and resolved on an any-KD midi SDS-PAGE gel (Bio-Rad, 5678123) and transferred to a PVDF membrane (Bio-Rad, 1704157) using transblot-turbo transfer system (Bio-Rad). Membranes were incubated in blocking buffer for 1 h at room temperature. To detect LGG-1 protein bands, membranes were incubated with a rabbit anti-LGG-1 polyclonal antibody (Thermofisher, PA5-116410) at 1:1000 dilution for 16 h at 4 °C, followed by incubation with a goat anti-rabbit IRDye800 antibody (Licor, 926-32211) at 1:4000 for 1 h at room temperature. LGG-1 protein bands were visualized on a Chemidoc™ MP imaging system (Bio-Rad).

### Microscopic imaging

For microscopic image acquisition except Figure S2, worms were mounted on glass slides with 2.5% agarose pad and immobilized with 20 mM or 25 mM sodium azide in M9. For Figure S2, worms were transferred to 35 mm MatTek glass bottom culture dishes (MatTek) and immobilized with 100 mM sodium azide in PBS. Z-stack images (step size 0.35 µm) were captured using a Leica TCS SP8X tandem scanning confocal microscope (Leica Microsystems) with a 40x 1.3 NA oil Apochromat CS2 objective. Fluorescent transgenic animals were excited using a white light supercontinuum laser at either 488 or 594 nm wavelengths. Fluorescence was captured using HyD spectral detectors. Images were acquired using LASX AF (Leica Microsystems) software and processed and analyzed using Fiji [73] or Volocity (v6.3; QuorumTechnologies, CAN) software.

### Puncta Quantification

GPF::LGG-1 puncta were quantified by taking confocal images of the posterior intestine from vulva to the tail. Maximum intensity projection images were generated from 70-step (0.35 µm/step) Z-stack images using Fiji. GFP::LGG-1 positive puncta were counted from at least 14 1-day adult worms of each genotype, then normalized by the volume of the posterior intestine. The volume of the posterior intestine was calculated by calculating the mKate2 fluorescence in the intestine using Fiji. Gaussian blur filtering and “Li” auto thresholding were applied to specifically select the mKate2 fluorescence region in the red channel of the whole Z-stack images. Then the 3D Objects Counter plugin was used to identify and calculate volume sizes of 3D red fluorescence objects in the Z-stack [74]. The volume size of the biggest object spanning the entire intestine region in the stack was selected as the posterior intestine’s volume size.

### Quantification of autophagic flux

Starvation-induced autophagy (endpoint assay): For each strain being tested, ~50 L4 stage animals were picked on to 60mm NGM plates 1 day prior to the experiment. After 24 hours, 50 day 1 (D1) adults were picked into a 1.7ml microfuge tube containing 1ml of PBS with 0.01% pluronic acid. Pluronic acid was used to prevent worms from sticking to the tube and pipette tips. Worms were rinsed three times with PBS and 0.01% pluronic acid to remove traces of bacteria. Animals were incubated on a benchtop rotator for 6 hours. Worms were then pelleted and resuspended in 300µl of PBS with 0.01% pluronic acid and 100µl of worm solution was dispensed into three wells of a NUNC 384-well plate. After 5 minutes on the bench to allow the worms to settle to the bottom, 60µl of supernatant was removed and replaced with 60µl of Levamisole (2.5 mg/ml). After 5 mins at room temperature, the 384-well plate was imaged in the brightfield, GFP and RFP fluorescence channel on the Thermofisher CellInsight CX-7 HCS as previously described [34,35,61].

Starvation-induced autophagy (6-hour time course assay): For each strain tested, ~ 300 L4 stage animals were picked on to 100mm NGM plates 1 day prior to the experiment. On the day of the experiment, 45 day 1 (D1) adults were transferred to a 1.7ml microfuge tube containing 1ml of PBS (+0.01 pluronic acid) at -6, -5, -4, -3, -2, -1, and 0 hours. At t=0, animals were pelleted and resuspended in 300 µl of PBS with 0.01% pluronic acid and 100µl of worm solution was dispensed into three wells of a NUNC 384-well plate. After 3 minutes on the bench to allow the worms to settle to the bottom, 60 µl of supernatant was removed and replaced with 60µl of Levamisole (2.5 mg/ml). After 5 mins at room temperature, the 384-well plate was imaged as described for the end-point starvation assay (above).

Basal autophagic flux assay (L1 larvae – day 7 adults): To measure basal autophagic flux, ~50 L, ~50 L2, ~30 L3, ~20 L4, ~15 D1, D3, D5 and D7 stage animals were picked and transferred in triplicate into wells of a NUNC 384-well plate containing 100 µl of PBS with 0.01% pluronic acid. After 5 minutes on the bench to allow the worms to settle to the bottom, 60 µl of supernatant was removed and replaced with 60 µl of levamisole (2.5 mg/ml). After 5 mins at room temperature, the 384-well plate was imaged as described for the end-point starvation assay (above). To control for picking bias, results were combined from experiments performed by three independent researchers.

Temperature-shift experiments: To measure basal autophagic flux of *daf-2(e1370)* mutants, a mixed population of *daf-2(e1370)* was maintained at 15°C (permissive temperate). 24 hours prior to the experiment, the mixed population of worms were transferred to 25°C (restrictive temperature). We also picked L4 stage animals and transferred them to 25°C. On the day of the experiment, ~50 L1, ~50 L2, ~30 L3, ~20 L4, ~15 D1 stage animals (which had spent the prior 24 hours of life at 25°C) were picked and transferred into a NUNC 384-well plates in triplicate and imaged as described for the end-point starvation assay (above). To control for picking bias, results were combined from experiments performed by three independent researchers.

Autophagic flux of *daf-2(1370)* dauers: *daf-2(e1370)* dauers and wild-type L3 control animals were mounted on 2.5% agarose pads, treated with 20 mM sodium azide. Z-stack of images of the intestine were captured by a Leica TCS SP8 microscope using a 40x objective. Sum projection of the Z-stack was used to quantify the fluorescence intensity by Fiji. The ratio of GFP:mKate2 was calculated and normalized to wild-type L3 controls.

### Phenotypic analysis

Brood size was determined by measuring the total number of eggs laid by an individual adult. 10 L4 stage animals were placed onto individual 60mm NGM plates and incubated at 20°C. The parent worm was transferred to a new plate daily for 5 days or until it had stopped laying eggs. The number of progeny on each plate was recorded and totaled. Experiments were repeated a minimum of three times for a total n≥30 animals.

Growth rate was measured by placing 20 adult hermaphrodites (24 h post-L4 stage) onto an NGM plate and allowing them to lay eggs for 2 h at 20°C. After the 2 h period, adults were removed, and remaining progeny were allowed to develop for 48 h at 22°C. The number of animals in each developmental stage was recorded. Experiments were repeated at least three times.

Lifespan analysis was performed by placing 30 L4 stage hermaphrodites onto NGM plates. Each plate was scored once a day for live and dead worms. An animal was considered dead if there was no response upon gentle prodding with a platinum wire. During the reproductive period (first 5 days), worms were transferred to a new plate every 24–48 h to avoid confusion with progeny. Dead worms were removed from the plate and discarded after scoring. Animals that crawled off the plate were censored from analysis. Each lifespan assay was repeated at least three times. Data were plotted as Kaplan–Meier survival curves and statistical significance was determined using the log-rank (Mantel–Cox) test.

### Autophagy compound screening

OP50 concentrate preparation for compound screening: A single colony of OP50-1 was placed in 50 ml LB broth and incubated at 37°C with vigorous shaking overnight. Fifty ml of the overnight culture was then added to a 1 L flask containing 500 ml LB broth and was incubated at 37°C with vigorous shaking for 6 hours until the OD_600_ of 1:5 diluted sample was  ~0.5. The bacteria was washed twice with PBS + 0.01% pluronic and concentrated to make the “4x OP50 concentrate”.

Preparation of animals for compound screening: Twenty-five adult animals expressing the AFR were placed on 100 mm NGM plate seeded with *E. coli* strain OP50. Approximately 4 days later, gravid adult worms were washed off and bleached to obtain eggs. Eggs were incubated at 20°C for 48 hours to obtain mostly L4 stage animals. Worms were washed off with PBS with 0.01% pluronic and counted. The volume was adjusted to prepare a “Worm solution” containing 750 worms per ml.

Autophagy compound library screening: One hundred nanoliter of 10 mM compound stock in 100% DMSO was dispensed into 384-well and stored at -80°C until required. Compound plates were thawed at room temperature for 30 minutes then centrifuged at 720 *g* using an Eppendorf swing bucket 5804R tabletop centrifuge. Equal volumes of the Worm solution and the 2x OP50 concentrate were combined before dispensing 40 µl (~15 worms) into each well using the MultiFlo high-speed peristaltic pump (BioTek). The final compound concentration was 25 µM. Plates were incubated at 20°C for 24 hours. Sixty microliters of levamisole (2.5 mg/ml) was dispensed into each well to anesthetize the animals prior to imaging. After 5 mins at room temperature, the 384-well plate was imaged in the brightfield, GFP and RFP fluorescence channels on the CellInsight CX-7 high-content imager.

8-Point dose response studies: For the 8-point dose response experiments, compound solutions were prepared at the following concentrations: 200, 62, 20, 6.25, 2, 0.625, 0.2 and 0.02 µM. Twenty microliters of the compound solution were then transferred to a 384-well plate. A 2x Worm/OP50 solution was prepared by mixing equal volumes of a worm solution containing 1500 worms per ml and 4X OP50 concentrate. Then 20 µl of the 2x Worm/OP50 solution was added to each well using the Multiflo high-speed peristaltic pump (BioTek). The final compound concentrations in the wells were 100, 31, 10, 3.125, 1, 0.3125, 0.1 and 0.01 µM. Plates were incubated at 20°C for 24 hours. Sixty microliters of levamisole (2.5 mg/ml) was dispensed into each well to anesthetize the animals prior to imaging. After 5 mins at room temperature, the 384-well plate was imaged in the brightfield, GFP and RFP fluorescence channels on the CellInsight CX-7 high-content imager.

### Pharyngeal pumping

Animals were incubated with 31 µM compound for 24 hours in 384-well plates as described for the drug screening. Animals were then transferred to an OP50-seeded NGM plate containing the same concentration of the compound then allowed to equilibrate for 30 minutes before videos were captured using the digital camera on the ScreenChip (NemaMetrix Inc) for a minimum of 2 minutes. Videos were then replayed at 0.5x speed and pharyngeal pumping was quantified by counting the terminal bulb movements.

### Bacterial OD following drug treatment

Compound solutions were prepared at the following concentrations: 200, 62, 20, 6.25, 2, 0.625, 0.2 and 0.02 µM as for the 8-point dose response experiment. Twenty microliters of the compound solution were then transferred to a 384-well plate. A 2x OP50 solution was prepared and 20 µl was added to each well using the Multiflo high-speed peristaltic pump (BioTek). The final compound concentrations in the wells were 100, 31, 10, 3.125, 1, 0.3125, 0.1 and 0.01 µM. Plates were incubated at 20 °C for 24 hours. The plate was vortexed and briefly centrifuged before measuring the OD_600_ on the Varioskan Lux Spectrophotometer (Thermofisher).

### Statistical evaluation

Statistical evaluation of data was performed using *Prism*® (GraphPad Software). In general, we used ANOVA to first test for significance, with follow up testing using Holm-Sidak correction for multiple tests or with a *t*-test if only two experimental groups were analyzed at the same time. The statistical significance of data in Figures 2, 4, S3A, S12A were determined using an ANOVA to first test for significance, with follow up test using Holm-Sidak correction for multiple tests. For developmental and aging experiments with only two genotypes performed at the same time (Figure 5A–C), multiple unpaired t-tests correcting for multiple comparisons using Holm-Sidak method were used between each developmental stage. For developmental and aging experiments with more than two genotypes analyzed at the same time (Figure 5D and 5E), a two-way ANOVA was performed looking at the interaction between stage and genotype, then follow up tests using Holm-Sidak correction for multiple tests were performed comparing genotypes within each stage. Statistical significance of Fig. S5B, S9B, S10, and S12B were performed using a student *t*-test. Statistical significance of developmental growth (Fig. S3B) was determined using a Chi-squared test. Statistical significance of lifespan data (Fig. S3C) was determined using a Mantel–Cox log-rank test. *P*-value summary: *P < 0.05, **P < 0.01, ***P < 0.001, ****P < 0.0001.

## Supplementary Material

Supplemental Material

Fig. S11.pdf

Fig. S9.pdf

Fig. S12.pdf

Fig. S4.pdf

Fig. S8.pdf

Table S3.docx

Fig. S3.pdf

Fig. S2.pdf

Fig. S6.pdf

Fig. S1.pdf

Supplementary figure legends.docx

Fig. S13.pdf

Fig. S7.pdf

Table S4.docx

Supplementary information_Figs and Tables.pdf

Fig. S10.pdf

Fig. S5.pdf

Table S2.xlsx

## Abbreviations

AFR: autophagic flux reporter

AFR(G116A): autophagic flux reporter, glycine 116 to alanine, mutant

ATZ: α1-antitrypsin Z

DMSO: dimethylsulfoxide

GFP: green fluorescent protein

mRFP: monomeric red fluorescent protein

mTOR: mechanistic target of rapamycin

OD: optical density;

OP50 *E. coli* strain, OP50

PE: phosphatidylethanolamine

L1: first larval stage; L2

second larval stage; L3: third larval stage

L4 fourth larval stage
