# Supplementary figures and images for: A fluorescent reporter for rapid assessment of autophagic flux reveals unique autophagy signatures during C. elegans post-embryonic development and identifies compounds that modulate autophagy
Source: Autophagy Rep. 2024 Jul 11;3(1):2371736. doi: 10.1080/27694127.2024.2371736 (PMC11271720; doi:10.1080/27694127.2024.2371736)

**Figure S11**

**A**

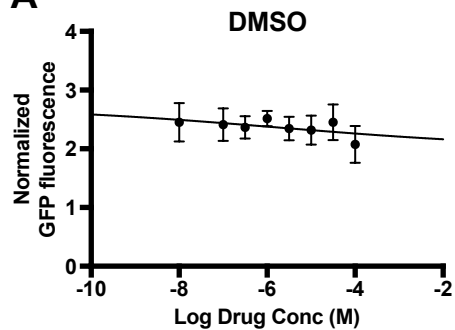

**B**

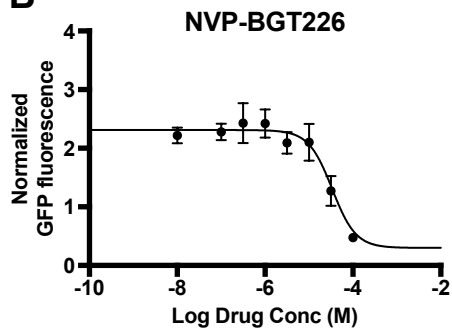

**C**

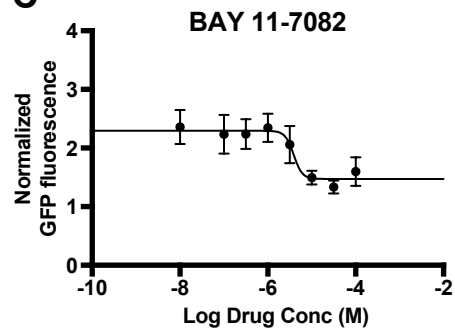

**D**

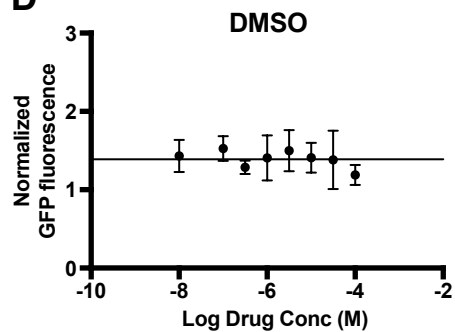

**E**

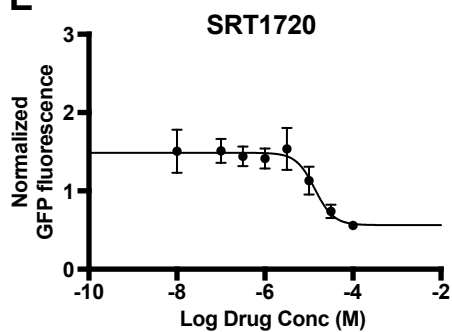

Supplement: Fig. S11.pdf [file KAUO_A_2371736_SM2337.pdf]

**Figure S9**

**A**

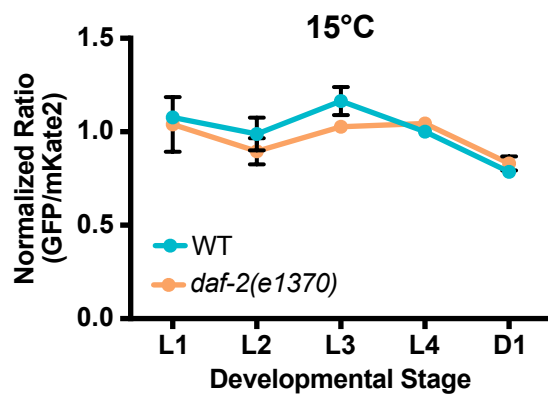

**B**

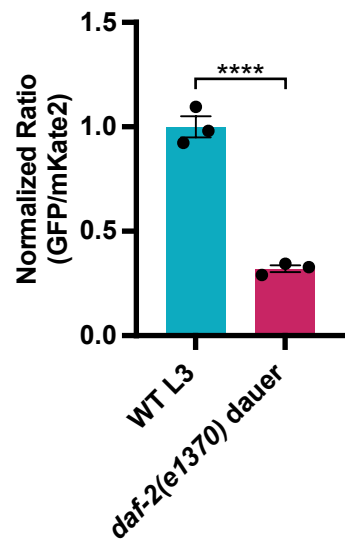

**C**

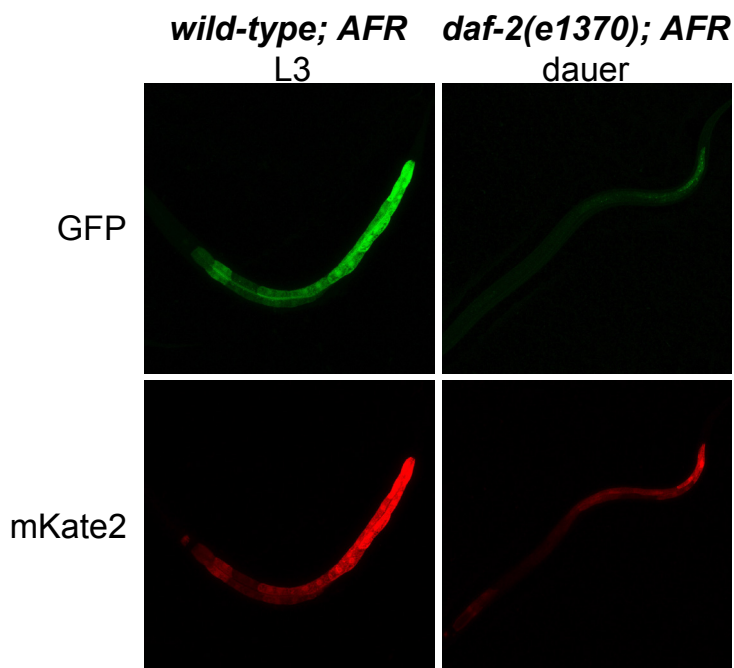

Supplement: Fig. S9.pdf [file KAUO_A_2371736_SM2336.pdf]

**Figure S12**

**A**

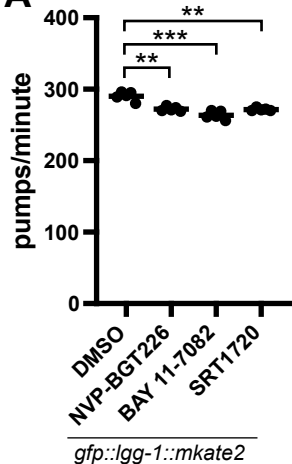

**B**

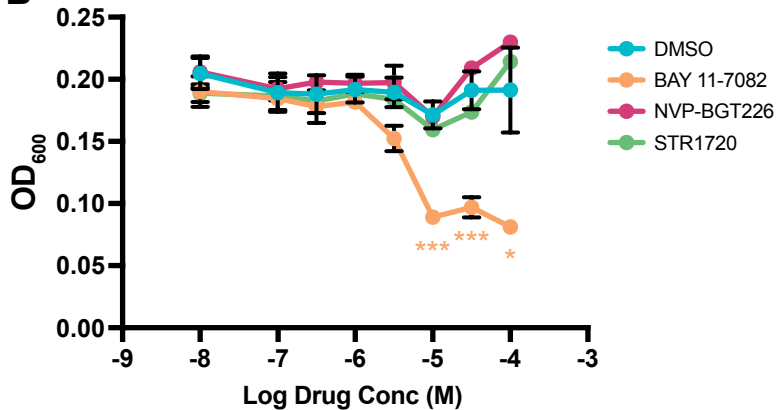

Supplement: Fig. S12.pdf [file KAUO_A_2371736_SM2335.pdf]

**Figure S4**

**A**

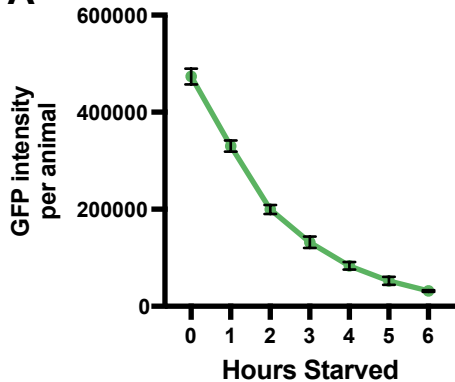

**B**

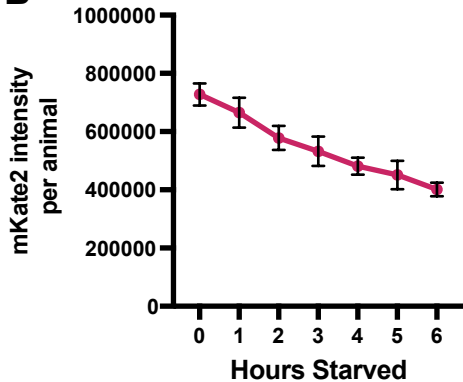

Supplement: Fig. S4.pdf [file KAUO_A_2371736_SM2334.pdf]

**Figure S8**

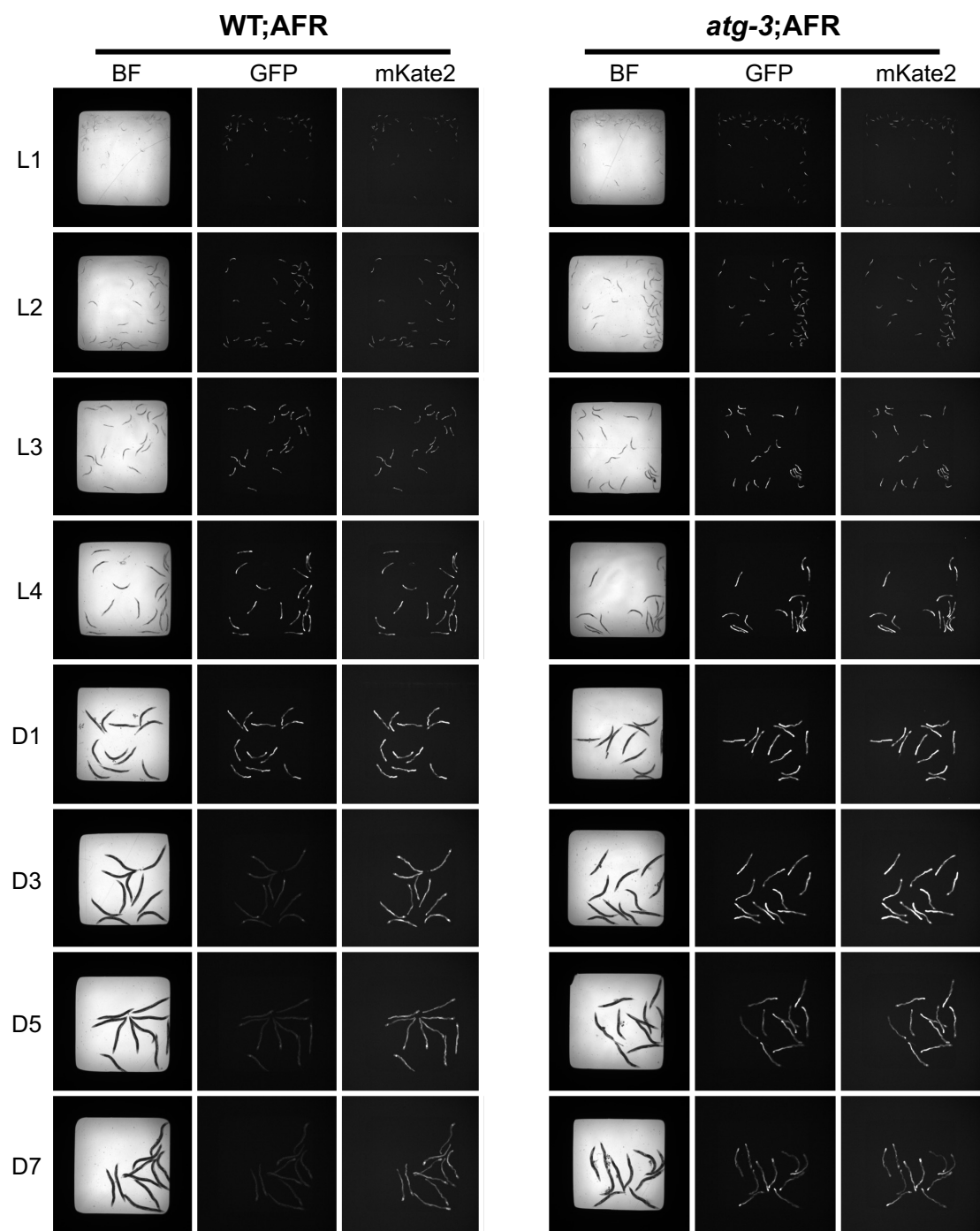

Supplement: Fig. S8.pdf [file KAUO_A_2371736_SM2333.pdf]

**Figure S3**

**A**

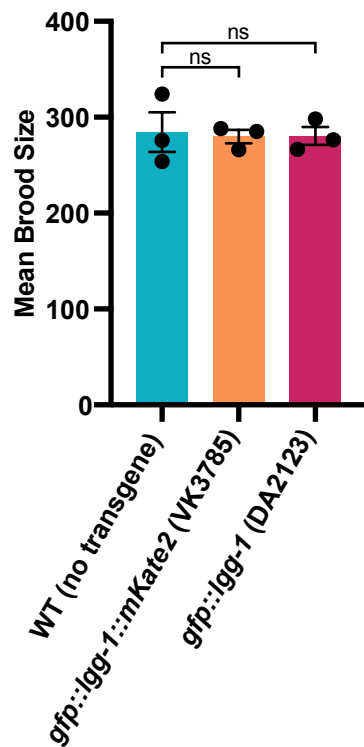

**B**

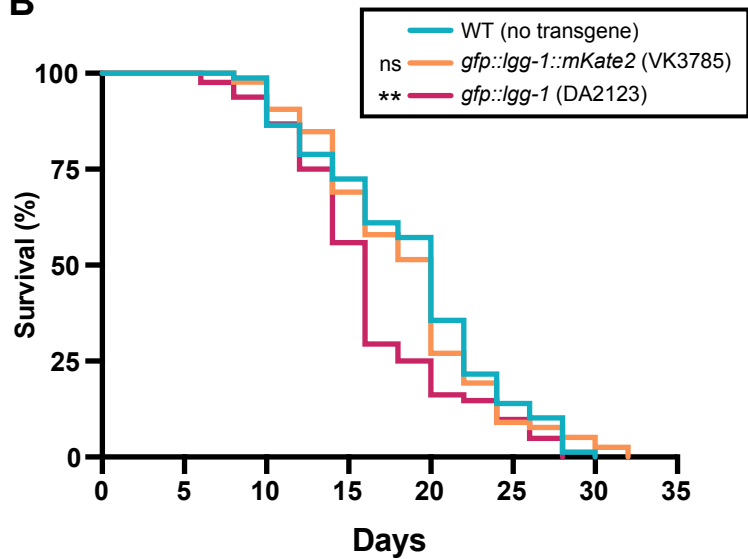

**C**

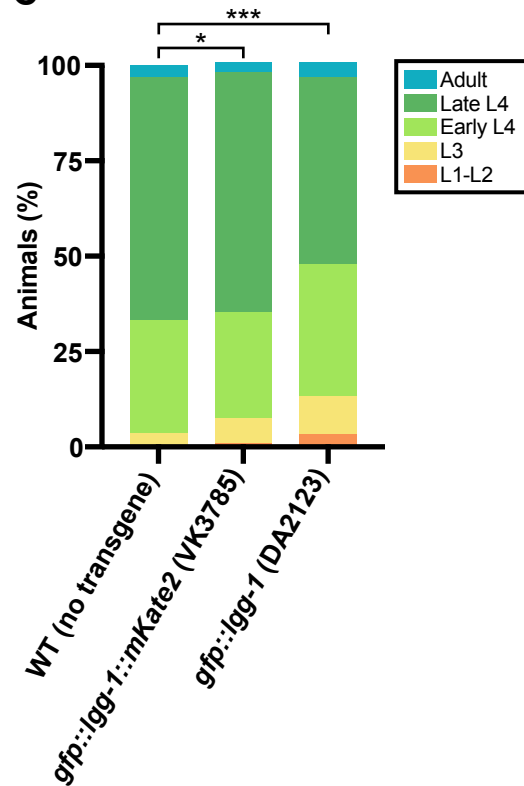

Supplement: Fig. S3.pdf [file KAUO_A_2371736_SM2331.pdf]

**Figure S2**

**A**

*nhx-2p::gfp*

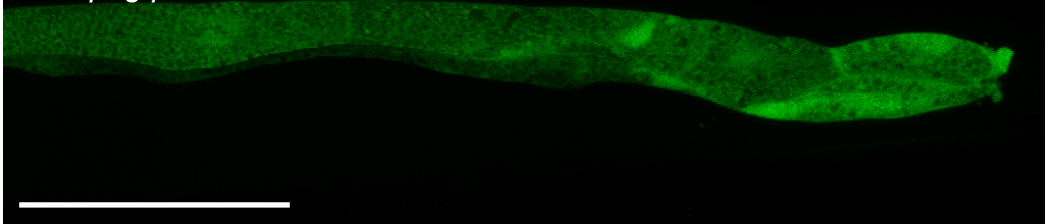

**B**

*nhx-2p::gfp; epg-5(tm3425)*

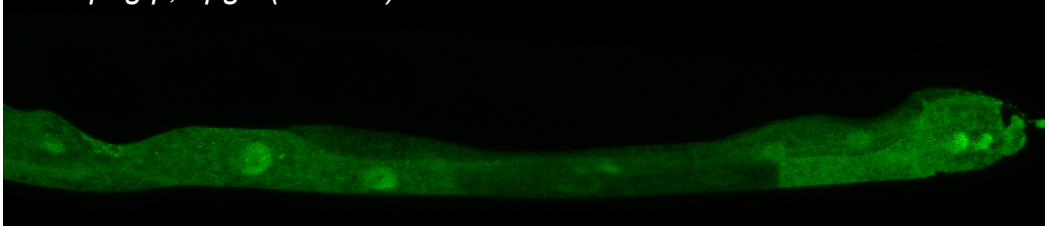

Supplement: Fig. S2.pdf [file KAUO_A_2371736_SM2329.pdf]

Figure S6

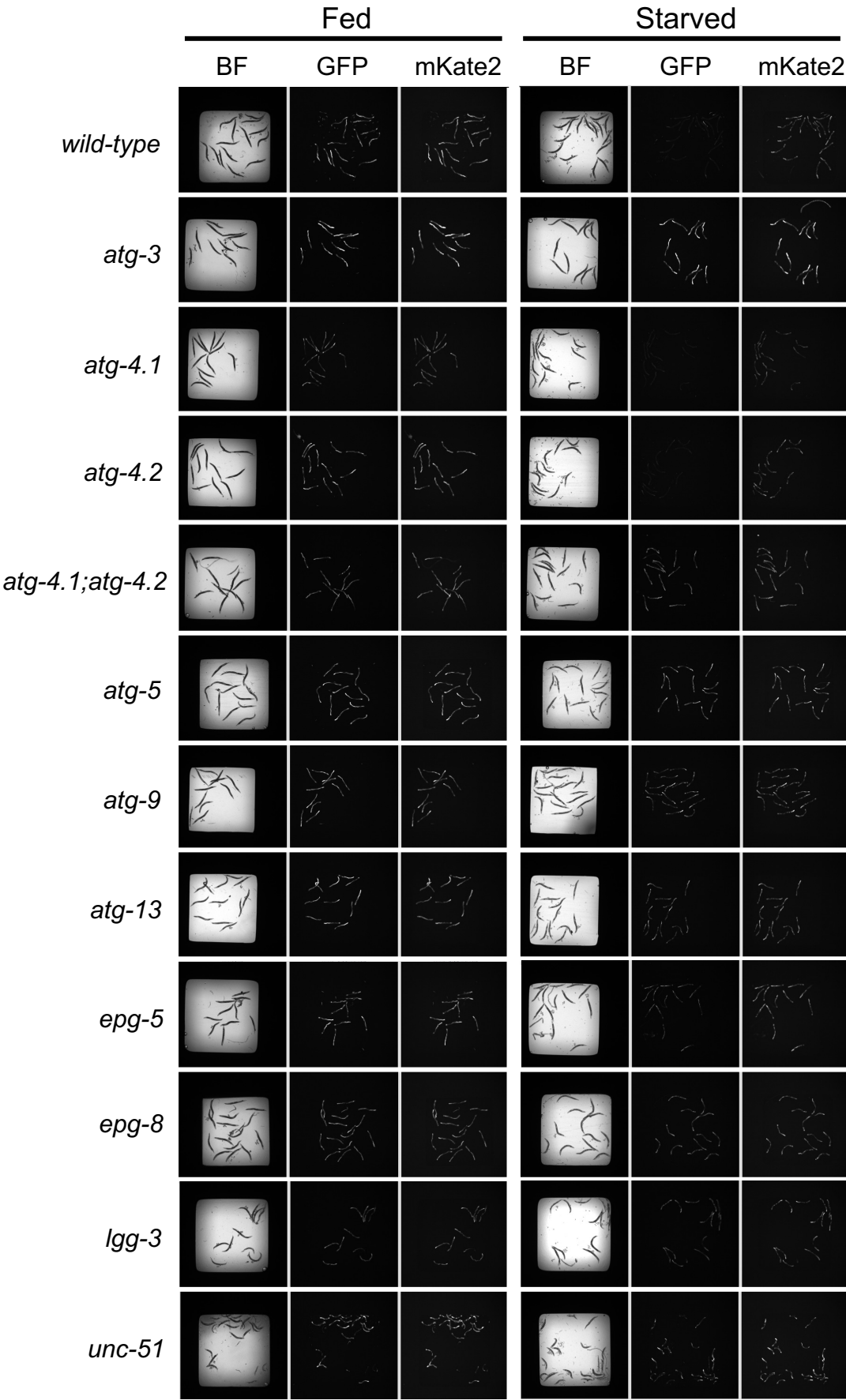

Supplement: Fig. S6.pdf [file KAUO_A_2371736_SM2328.pdf]

# Figure S1

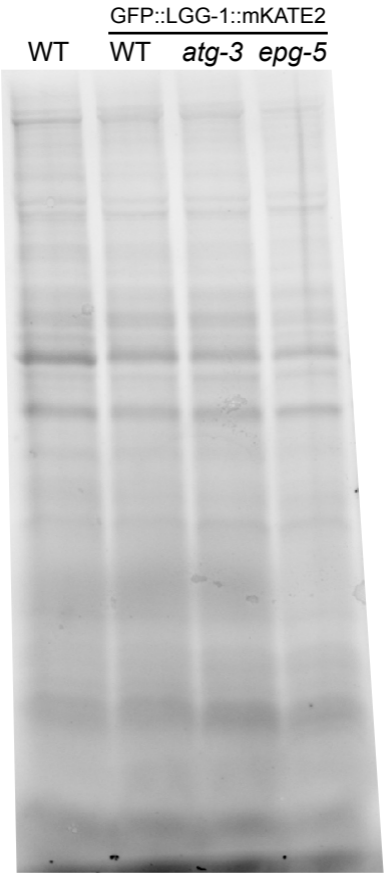

Stain-free gel

Supplement: Fig. S1.pdf [file KAUO_A_2371736_SM2327.pdf]

**Figure S13**

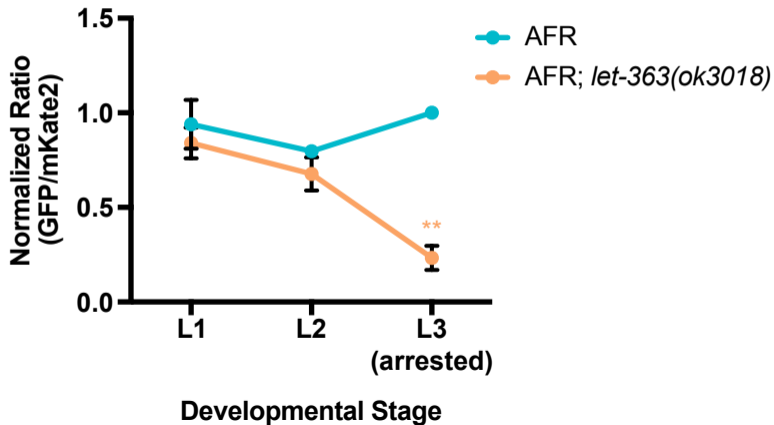

Supplement: Fig. S13.pdf [file KAUO_A_2371736_SM2325.pdf]

Figure S7

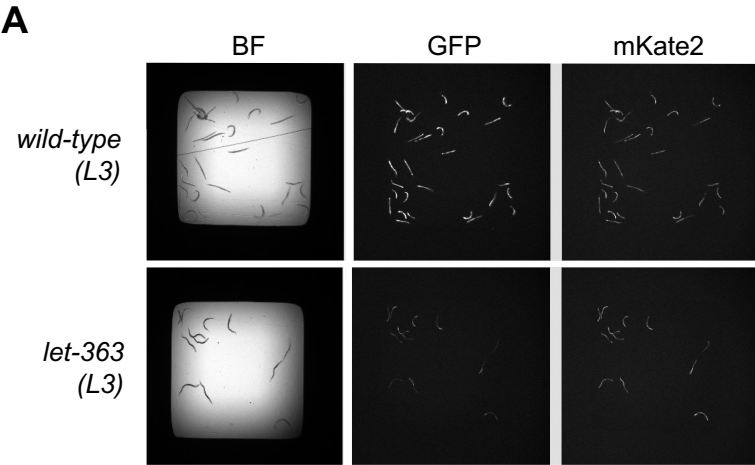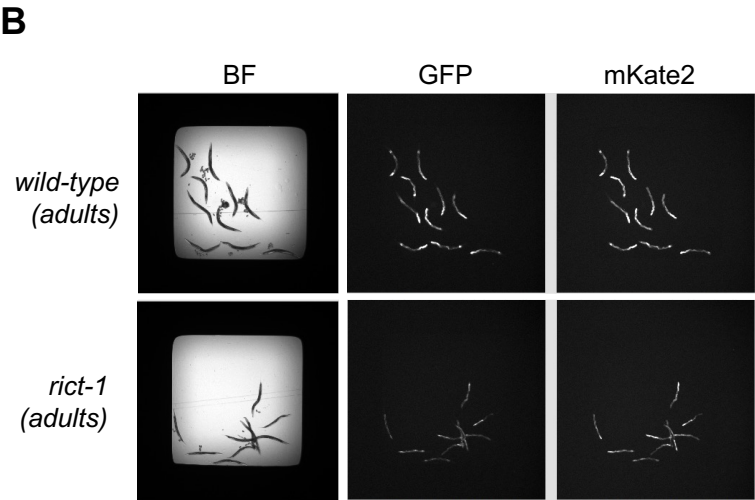

Supplement: Fig. S7.pdf [file KAUO_A_2371736_SM2324.pdf]

**Figure S10**

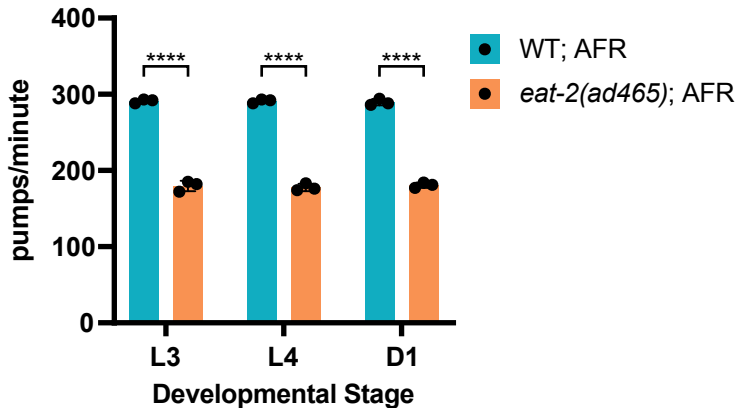

Supplement: Fig. S10.pdf [file KAUO_A_2371736_SM2321.pdf]

**Figure S5**

**A**

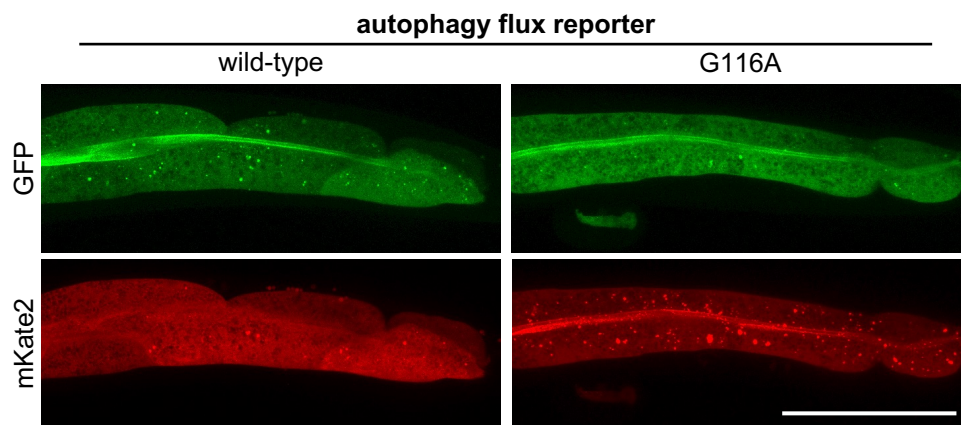

**B**

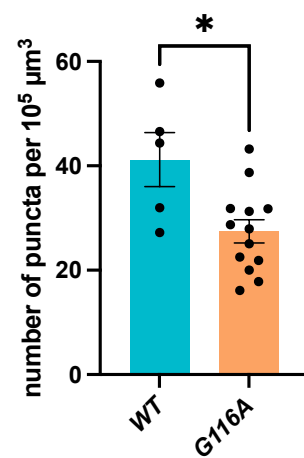

Supplement: Fig. S5.pdf [file KAUO_A_2371736_SM2320.pdf]
