# Supplementary material for: A fluorescent reporter for rapid assessment of autophagic flux reveals unique autophagy signatures during C. elegans post-embryonic development and identifies compounds that modulate autophagy
Source: Autophagy Rep. 2024 Jul 11;3(1):2371736. doi: 10.1080/27694127.2024.2371736 (PMC11271720; doi:10.1080/27694127.2024.2371736)
Supplement: Table S3.docx [file KAUO_A_2371736_SM2332.docx]

Table S3. Lifespan analyses

| Strain | Mean +/-SEM (days) | 75^th^ percentile (days) | *P* values | n | Fig. |
| --- | --- | --- | --- | --- | --- |
| Autophagic Flux Reporter (AFR) has comparable lifespan to wild-type VC2010 animals | | | | | |
| wild-type (VC2010) | 18.71 +/- 0.64 | 22 |  | 79/90 | Fig. S3B |
| gfp::lgg-1::mKate2 (VK3785) | 18.28 +/- 0.62 | 22 | 0.6117 | 80/90 |  |
| *gfp:*:lgg-1 (DA2123) | 15.91 +/- 0.63 | 18 | 0.0043 | 68/90 |  |
