# Supplementary material for: A fluorescent reporter for rapid assessment of autophagic flux reveals unique autophagy signatures during C. elegans post-embryonic development and identifies compounds that modulate autophagy
Source: Autophagy Rep. 2024 Jul 11;3(1):2371736. doi: 10.1080/27694127.2024.2371736 (PMC11271720; doi:10.1080/27694127.2024.2371736)
Supplement: Supplementary figure legends.docx [file KAUO_A_2371736_SM2326.docx]

###### A fluorescent reporter for rapid assessment of autophagic flux reveals unique autophagy signatures during C. elegans post-embryonic development and identifies compounds that modulate autophagy.

###### Zachary D. Dawson, Hemalatha Sundaramoorthi, Suk Regmi, Bo Zhang, Stephanie Morrison, Sara M. Fielder, Jessie R. Zhang, Hieu Hoang, David H. Perlmutter, Cliff J. Luke, Gary A. Silverman, and Stephen C. Pak

###### SUPPLEMENTARY FIGURE LEGENDS

**Figure S1.** Differences in LGG-1 band patterns are not due to unequal protein loading. Stain-free image of gel used for LGG-1 Western blot in Fig. 1D showing similar total protein loading.

**Figure S2.** GFP does not form puncta in *epg-5(tm3425)*. Maximum intensity confocal images of

(**A**) *nhx-2p::gfp* and (**B**) *nhx-2p::gfp; epg-5(tm3425)* animals showing diffuse cytoplasmic and nuclear GFP expression. GFP-positive puncta are absent in *nhx-2p::gfp; epg-5(tm3425)* animals. Compare with images in Fig. 2C. Day 1 adults are shown.

**Figure S3.** Expression of autophagic flux reporter (AFR) does not alter organismal fitness. (**A**) Brood size, (**B**) lifespan, and (**C**) grow rate through post-embryonic development of wild type (non-transgenic), *gfp::lgg-1::mKate2* (AFR) and *gfp::lgg-1* (DA2123) strains, respectively. Data are an average of three independent experiments. Lifespan data including mean survival times are provided in Table S3.

**Figure S4.** GFP::LGG-1 fluorescence decreases at a faster rate than mKate2 upon starvation. (**A**) GFP and (**B**) mKate2 fluorescence intensities per animal over 6 hours of starvation. The *t*1/2 of GFP was ~1.7 hours. The *t*1/2 of mKate2 was > 6 hours.

**Figure S5.** AFR(G116A) mutant control. (**A**) Maximum intensity confocal images of

*nhx-2p::gfp::lgg-1::mKate2* (left) and *nhx-2p::gfp::lgg-1(G116A)::mKate2 control* (right). Day 1 adults are shown. (**B**) GFP puncta quantification. Shown are mean ± SD.

**Figure S6.** Representative well images of wild-type and autophagy mutants expressing the *gfp::lgg-1::mKate2* (AFR). All animals are day 1 adults.

**Figure S7.** Representative well images of wild-type, *let-363(ok3018)* and *rict-1(ft7)* mutant animals. *let-363(ok3018)* animals are homozygous lethal at the L3/L4 stage. As such, images were taken of young L3 animals (**A**). *rict-1(ft7)* mutants are homozygous viable. As such, images are of day 1 adults (**B**).

**Figure S8.** Representative well images of wild-type (left) and *atg-3(bp412)* (right) carrying the *nhx-2p::gfp::lgg-1::mKate2* transgene. L1 to D7 adult stages are shown. ~50 L1, ~50 L2, ~30 L3, ~20 L4, ~15 D1, D3, D5 and D7 stage animals are in each well.

**Figure S9.** Autophagic flux of *daf-2(e1370)* mutants. (**A**) GFP:mKate2 ratios of wild-type and *daf-2(e1370)* animals through postembryonic development at 15 ºC. (**B**) GFP:mKate2 ratios of wild-type L3 and *daf-2(e1370)* dauer animals cultured at 25 ºC as determined by confocal microscopy. GFP/mKate2 fluorescence was calculated using ImageJ. (**C**) Representative maximum intensity confocal images of animals in (**B**).

**Figure S10.** Pharyngeal pumping rates are lower in *eat-2(ad465)* mutants. Pharyngeal pumping rates of wild-type (blue) and *eat-2(ad465)* mutants (orange) at L3, L4 and D1 adult stages. Error bars represent mean ± SEM.

**Figure S11.** Eight-point dose response curves of nhx-2p::sGFP::ATZ expressing animals treated with (**A**) DMSO, (**B**) NVP-BGT226, (**C**) BAY 11-7082, (**D**) DMSO, and (**E**) SRT1720. Graphs are representative of three independent experiments. Error bars denote mean ± SEM.

**Figure S12.** Assessment of pharyngeal pumping rates and bacterial OD following drug treatment. (**A**) Pharyngeal pumping rates of L4 stage animals treated with hit compounds for 24 hours. All animals carried the *nhx-2p::gfp::lgg-1::mKate2* transgene. (B) OD_600_ of OP50 in wells treated with DMSO or hit compounds for 24 hours.

**Figure S13.** Autophagic flux of *let-363(ok3018)* mutants. GFP:mKate2 ratios of wild-type and *let-363(ok3018)* animals at L1, L2 and L3 larval stages.
