# Supplementary material for: A fluorescent reporter for rapid assessment of autophagic flux reveals unique autophagy signatures during C. elegans post-embryonic development and identifies compounds that modulate autophagy
Source: Autophagy Rep. 2024 Jul 11;3(1):2371736. doi: 10.1080/27694127.2024.2371736 (PMC11271720; doi:10.1080/27694127.2024.2371736)
Supplement: Table S4.docx [file KAUO_A_2371736_SM2323.docx]

**Table S4. Maps and sequences of AFR and AFR(G116A) constructs**

| **Wild-type AFR construct**  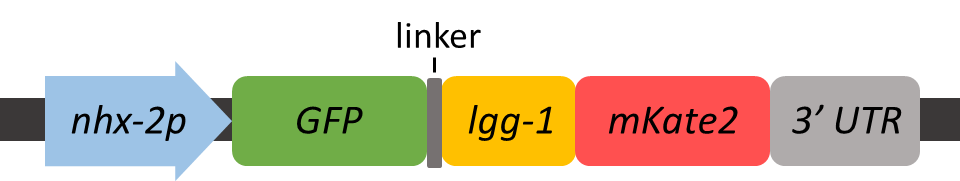 |
| --- |
| tccttccaagttattgacaagtattaactgcacttctcataaaaatttttcaaatagatctatttagtctggaataggaatacttttggcaattttcttgagaatcatagaagacgaatttctgaactgctctttgtgaagccacgcccatatattggcatttttgtatccaattttttgtagagtacctgaatcattgtcactacatctgctaaacaatatatggaagttctatgagaggtgaaaggtaaatcatataacaactgtaataacatagaaatgattgtagtatatttccaattaggctgagttgatcttattgaagagtttgaatcatagcaattttttcctcttttagtatttttcaaaacaaattttcacaatgttagatgtagtatttagcattaaggcatctgtaatctcccagaaatgttttgggaataaaaccctttgactctttggtccttttttgctccaaataatatctgattttaacccgttctgatctactttttacctttcgatcgtcacatccgcgtacacgtttctcttgctctcaattgcttatctttatggccttttcccccactttttgtcgactatcatgcggacgtggcttccttgctccctcccggtttttttgaaaacacttgtgaatttattttttgtggtcaaaattacgaaacatttgaattctcaatctatttatagaccgggtgtggtatgttatgccatgtttttgtcattgccaaatgaacatcgatattttaattttctgaacttaccgtaccccctgcgttactattttatttttaatttgtataatttcattgctataatttgttgtccttgaaacattgaagatatcgaagaaataatgacaaaaatcagatagaacaatttttcctactcatcaactcacatttttctattttctcttgtgcgaaaaaaatagtatattgggaatatttatagtattgcaacttttctcttagttcgcagagttgcaatactactagatccctaatagaggaaatatggtgaccagctttttgcaattggaaagttgggaaaaaagtatatatattaatagacttacattcttgtagttcgtagaatttttcaggaaatctgacacgcaagactttttcaaaaagtttttttttttaatttggcaatctgccaaaacgtttactgaacattttgaaaacatctaccagtacctttaacacaacgaaaaattgttctaagcaatattaactgtgataattgatgttaagacctgaaatttaatcacaaatttcacctggaaatgacagtaatcggcttctaaatactgtcgacacaagtgccatacctaagattccaatcctattaccatgtttcccaatttcattctctttcgtatctctatttctcttctttattatcaattactttttatcagttcttcgtttgctttaacatcaagagcacatagcgctctctcgtctctctctcatgctctttttgaacttttacaaaaaactatttccggttgttttgattcttggaatttgaaataattttcagtgattaaatctagaggatccccgggattggccaaaggacccaaag*gtatgtttcgaatgatactaacataacatagaacattttcag*gaggacccttggctagcctcgagATGAGTAAAGGAGAAGAACTTTTCACTGGAGTTGTCCCAATTCTTGTTGAATTAGATGGTGATGTTAATGGGCACAAATTTTCTGTCAGTGGAGAGGGTGAAGGTGATGCAACATACGGAAAACTTACCCTTAAATTTATTTGCACTACTGGAAAACTACCTGTTCCATGG*gtaagtttaaacatatatatactaactaaccctgattatttaaattttcag*CCAACACTTGTCACTACTTTCTGTTATGGTGTTCAATGCTTTTCAAGATACCCAGATCATATGAAACGGCATGACTTTTTCAAGAGTGCCATGCCCGAAGGTTATGTACAGGAAAGAACTATATTTTTCAAAGATGACGGGAACTACAAGACAC*gtaagtttaaacagttcggtactaactaaccatacatatttaaattttcag*GTGCTGAAGTCAAGTTTGAAGGTGATACCCTTGTTAATAGAATCGAGTTAAAAGGTATTGATTTTAAAGAAGATGGAAACATTCTTGGACACAAATTGGAATACAACTATAACTCACACAATGTATACATCATGGCAGACAAACAAAAGAATGGAATCAAAGTT*gtaagtttaaacatgattttactaactaactaatctgatttaaattttcag*AACTTCAAAATTAGACACAACATTGAAGATGGAAGCGTTCAACTAGCAGACCATTATCAACAAAATACTCCAATTGGCGATGGCCCTGTCCTTTTACCAGACAACCATTACCTGTCCACACAATCTGCCCTTTCGAAAGATCCCAACGAAAAGAGAGACCACATGGTCCTTCTTGAGTTTGTAACAGCTGCTGGGATTACACATGGCATGGATGAACTATACAAAGGTGGCGGTGGCTCGGGCGGTGGTGGGTCGGGTGGCGGCGGAATGAAGTGGGCTTACAAGGAGGAGAACAACTTTGAGAAGCGTCGTGCCGAAGGAGACAAGATCCGCAGAAAGTACCCAGACCGTATTCCAGTGATTGTTGAGAAAGCACCAAAGTCAAAGCTCCATGACTTGGATAAGAAGAAGTACTTGGTCCCATCCGATCTTACTGTTGGACAGTTCTACTTCCTCATCAGAAAACGCATCCAACTTCGTCCAGAAGATGCTCTGTTCTTCTTTGTCAACAATGTCATTCCACAAACCATGACCACAATGGGACAACTCTACCAG*gtaactaaccagtcgtttattttcatttaattaacccttttctttattacag*GACCATCACGAGGAAGACTTGTTCCTTTACATCGCCTACAGTGACGAAAGTGTGTATGGAGGAGAGGTCGAAAAGAAGGAAATGGTGAGCGAGCTGATTAAGGAGAACATGCACATGAAGCTGTACATGGAGGGCACCGTGAACAACCACCACTTCAAGTGCACATCCGAGGGCGAAGGCAAGCCCTACGAGGGCACCCAGACCATGAGAATCAAGGCGGTCGAGGGCGGCCCTCTCCCCTTCGCCTTCGACATCCTGGCTACCAGCTTCATGTACGGCAGCAAAACCTTCATCAACCACACCCAGGGCATCC*gtaagtttaaacatgatattactaactaacaaagctcatttaaattttcag*CCGACTTCTTTAAGCAGTCCTTCCCCGAGGGCTTCACATGGGAGAGAGTCACCACATACGAAGACGGGGGCGTGCTGACCGCTACCCAGGACACCAGCCTCCAGGACGGCTGCCTCATCTACAACGTCAAGATCAGAGGGGTGAACTTCCCATCCAACGGCCCTGTGATGCAGAAGAAAACACTCGGCTGGGAGGCCTCCACCGAGACC*gttagttttaaatggcaatgtaccaatttaaagttttcaaacatgtttcag*CTGTACCCCGCTGACGGCGGCCTGGAAGGCAGAGCCGACATGGCCCTGAAGCTCGTGGGCGGGGGCCACCTGATCTGCAACTTGAAGACCACATACAGATCCAAGAAACCCGCTAAGAACCTCAAGATGCCCGGCGTCTACTATGTGGACAGAAGACTGGAAAGAATCAAGGAGGCCGACAAAGAGACCTACGTCGAGCAGCACGAGGTGGCTGTGGCCAGATACTGCGACCTCCCTAGCAAACTGGGGCACAGATAAgagctccgcatcggccgctgtcatcagatcgccatctcgcgcccgtgcctctgacttctaagtccaattactcttcaacatccctacatgctctttctccctgtgctcccaccccctatttttgttattatcaaaaaacttctcttaatttctttgttttttagcttcttttaagtcacctctaacaatgaaattgtgtagattcaaaaatagaattaattcgtaataaaaagtcgaaaaaaattgtgctccctccccccattaataataattctatcccaaaatctacacaatgttctgtgtacacttcttatgttttttacttctgataaatttttttgaaacatcatagaaaaaaccgcacacaaaataccttatcatatgttacgtttcagtttatgaccgcaatttttatttcttcgcacgtctgggcctctcatgacgtcaaatcatgctcatcgtgaaaaagttttggagtatttttggaatttttcaatcaagtgaaagtttatgaaattaattttcctgcttttgctttttggggtttcccctattgtttgtcaagatttcgaggacggcgtttttcttgctaaaatcacaagtattgatgagcacgatgcaagaaagatcggaagaaggtttgggtttgaggctcagtggaaggtgagtagaagttgataatttgaaagtggagtagtgtctatggggtttttgccttaaatgacagaatacattcccaatataccaaacataactgtttctactagtcggccgtacgggccctttcgtctcgcgcgtttcggtgatgacggtgaaaacctctgacacatgcagctcccggagacggtcacagcttgtctgtaagcggatgccgggagcagacaagcccgtcagggcgcgtcagcgggtgttggcgggtgtcggggctggcttaactatgcggcatcagagcagattgtactgagagtgcaccatatgcggtgtgaaataccgcacagatgcgtaaggagaaaataccgcatcaggcggccttaagggcctcgtgatacgcctatttttataggttaatgtcatgataataatggtttcttagacgtcaggtggcacttttcggggaaatgtgcgcggaacccctatttgtttatttttctaaatacattcaaatatgtatccgctcatgagacaataaccctgataaatgcttcaataatattgaaaaaggaagagtatgagtattcaacatttccgtgtcgcccttattcccttttttgcggcattttgccttcctgtttttgctcacccagaaacgctggtgaaagtaaaagatgctgaagatcagttgggtgcacgagtgggttacatcgaactggatctcaacagcggtaagatccttgagagttttcgccccgaagaacgttttccaatgatgagcacttttaaagttctgctatgtggcgcggtattatcccgtattgacgccgggcaagagcaactcggtcgccgcatacactattctcagaatgacttggttgagtactcaccagtcacagaaaagcatcttacggatggcatgacagtaagagaattatgcagtgctgccataaccatgagtgataacactgcggccaacttacttctgacaacgatcggaggaccgaaggagctaaccgcttttttgcacaacatgggggatcatgtaactcgccttgatcgttgggaaccggagctgaatgaagccataccaaacgacgagcgtgacaccacgatgcctgtagcaatggcaacaacgttgcgcaaactattaactggcgaactacttactctagcttcccggcaacaattaatagactggatggaggcggataaagttgcaggaccacttctgcgctcggcccttccggctggctggtttattgctgataaatctggagccggtgagcgtgggtctcgcggtatcattgcagcactggggccagatggtaagccctcccgtatcgtagttatctacacgacggggagtcaggcaactatggatgaacgaaatagacagatcgctgagataggtgcctcactgattaagcattggtaactgtcagaccaagtttactcatatatactttagattgatttaaaacttcatttttaatttaaaaggatctaggtgaagatcctttttgataatctcatgaccaaaatcccttaacgtgagttttcgttccactgagcgtcagaccccgtagaaaagatcaaaggatcttcttgagatcctttttttctgcgcgtaatctgctgcttgcaaacaaaaaaaccaccgctaccagcggtggtttgtttgccggatcaagagctaccaactctttttccgaaggtaactggcttcagcagagcgcagataccaaatactgttcttctagtgtagccgtagttaggccaccacttcaagaactctgtagcaccgcctacatacctcgctctgctaatcctgttaccagtggctgctgccagtggcgataagtcgtgtcttaccgggttggactcaagacgatagttaccggataaggcgcagcggtcgggctgaacggggggttcgtgcacacagcccagcttggagcgaacgacctacaccgaactgagatacctacagcgtgagctatgagaaagcgccacgcttcccgaagggagaaaggcggacaggtatccggtaagcggcagggtcggaacaggagagcgcacgagggagcttccagggggaaacgcctggtatctttatagtcctgtcgggtttcgccacctctgacttgagcgtcgatttttgtgatgctcgtcaggggggcggagcctatggaaaaacgccagcaacgcggcctttttacggttcctggccttttgctggccttttgctcacatgttctttcctgcgttatcccctgattctgtggataaccgtattaccgcctttgagtgagctgataccgctcgccgcagccgaacgaccgagcgcagcgagtcagtgagcgaggaagcggaagagcgcccaatacgcaaaccgcctctccccgcgcgttggccgattcattaatgcagctggcacgacaggtttcccgactggaaagcgggcagtgagcgcaacgcaattaatgtgagttagctcactcattaggcaccccaggctttacactttatgcttccggctcgtatgttgtgtggaattgtgagcggataacaatttcacacaggaaacagctatgaccatgattacgccaagct  ----- *nhx-2* promoter  ----- GFP coding region  *Italics*: introns  ----- linker region  ----- LGG-1 coding region  ----- mKate2 coding region  underlined text: *unc-54* 3’ UTR |
|  |
| **AFR(G116A) construct**  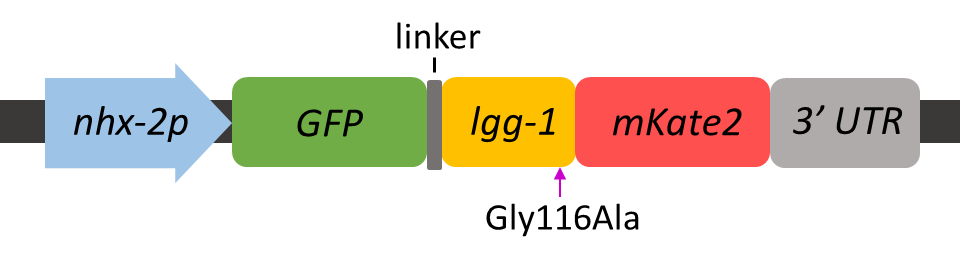 |
| tccttccaagttattgacaagtattaactgcacttctcataaaaatttttcaaatagatctatttagtctggaataggaatacttttggcaattttcttgagaatcatagaagacgaatttctgaactgctctttgtgaagccacgcccatatattggcatttttgtatccaattttttgtagagtacctgaatcattgtcactacatctgctaaacaatatatggaagttctatgagaggtgaaaggtaaatcatataacaactgtaataacatagaaatgattgtagtatatttccaattaggctgagttgatcttattgaagagtttgaatcatagcaattttttcctcttttagtatttttcaaaacaaattttcacaatgttagatgtagtatttagcattaaggcatctgtaatctcccagaaatgttttgggaataaaaccctttgactctttggtccttttttgctccaaataatatctgattttaacccgttctgatctactttttacctttcgatcgtcacatccgcgtacacgtttctcttgctctcaattgcttatctttatggccttttcccccactttttgtcgactatcatgcggacgtggcttccttgctccctcccggtttttttgaaaacacttgtgaatttattttttgtggtcaaaattacgaaacatttgaattctcaatctatttatagaccgggtgtggtatgttatgccatgtttttgtcattgccaaatgaacatcgatattttaattttctgaacttaccgtaccccctgcgttactattttatttttaatttgtataatttcattgctataatttgttgtccttgaaacattgaagatatcgaagaaataatgacaaaaatcagatagaacaatttttcctactcatcaactcacatttttctattttctcttgtgcgaaaaaaatagtatattgggaatatttatagtattgcaacttttctcttagttcgcagagttgcaatactactagatccctaatagaggaaatatggtgaccagctttttgcaattggaaagttgggaaaaaagtatatatattaatagacttacattcttgtagttcgtagaatttttcaggaaatctgacacgcaagactttttcaaaaagtttttttttttaatttggcaatctgccaaaacgtttactgaacattttgaaaacatctaccagtacctttaacacaacgaaaaattgttctaagcaatattaactgtgataattgatgttaagacctgaaatttaatcacaaatttcacctggaaatgacagtaatcggcttctaaatactgtcgacacaagtgccatacctaagattccaatcctattaccatgtttcccaatttcattctctttcgtatctctatttctcttctttattatcaattactttttatcagttcttcgtttgctttaacatcaagagcacatagcgctctctcgtctctctctcatgctctttttgaacttttacaaaaaactatttccggttgttttgattcttggaatttgaaataattttcagtgattaaatctagaggatccccgggattggccaaaggacccaaag*gtatgtttcgaatgatactaacataacatagaacattttcag*gaggacccttggctagcctcgagATGAGTAAAGGAGAAGAACTTTTCACTGGAGTTGTCCCAATTCTTGTTGAATTAGATGGTGATGTTAATGGGCACAAATTTTCTGTCAGTGGAGAGGGTGAAGGTGATGCAACATACGGAAAACTTACCCTTAAATTTATTTGCACTACTGGAAAACTACCTGTTCCATGG*gtaagtttaaacatatatatactaactaaccctgattatttaaattttcag*CCAACACTTGTCACTACTTTCTGTTATGGTGTTCAATGCTTTTCAAGATACCCAGATCATATGAAACGGCATGACTTTTTCAAGAGTGCCATGCCCGAAGGTTATGTACAGGAAAGAACTATATTTTTCAAAGATGACGGGAACTACAAGACAC*gtaagtttaaacagttcggtactaactaaccatacatatttaaattttcag*GTGCTGAAGTCAAGTTTGAAGGTGATACCCTTGTTAATAGAATCGAGTTAAAAGGTATTGATTTTAAAGAAGATGGAAACATTCTTGGACACAAATTGGAATACAACTATAACTCACACAATGTATACATCATGGCAGACAAACAAAAGAATGGAATCAAAGTT*gtaagtttaaacatgattttactaactaactaatctgatttaaattttcag*AACTTCAAAATTAGACACAACATTGAAGATGGAAGCGTTCAACTAGCAGACCATTATCAACAAAATACTCCAATTGGCGATGGCCCTGTCCTTTTACCAGACAACCATTACCTGTCCACACAATCTGCCCTTTCGAAAGATCCCAACGAAAAGAGAGACCACATGGTCCTTCTTGAGTTTGTAACAGCTGCTGGGATTACACATGGCATGGATGAACTATACAAAGGTGGCGGTGGCTCGGGCGGTGGTGGGTCGGGTGGCGGCGGAATGAAGTGGGCTTACAAGGAGGAGAACAACTTTGAGAAGCGTCGTGCCGAAGGAGACAAGATCCGCAGAAAGTACCCAGACCGTATTCCAGTGATTGTTGAGAAAGCACCAAAGTCAAAGCTCCATGACTTGGATAAGAAGAAGTACTTGGTCCCATCCGATCTTACTGTTGGACAGTTCTACTTCCTCATCAGAAAACGCATCCAACTTCGTCCAGAAGATGCTCTGTTCTTCTTTGTCAACAATGTCATTCCACAAACCATGACCACAATGGGACAACTCTACCAG*gtaactaaccagtcgtttattttcatttaattaacccttttctttattacag*GACCATCACGAGGAAGACTTGTTCCTTTACATCGCCTACAGTGACGAAAGTGTGTATGCAGGAGAGGTCGAAAAGAAGGAAATGGTGAGCGAGCTGATTAAGGAGAACATGCACATGAAGCTGTACATGGAGGGCACCGTGAACAACCACCACTTCAAGTGCACATCCGAGGGCGAAGGCAAGCCCTACGAGGGCACCCAGACCATGAGAATCAAGGCGGTCGAGGGCGGCCCTCTCCCCTTCGCCTTCGACATCCTGGCTACCAGCTTCATGTACGGCAGCAAAACCTTCATCAACCACACCCAGGGCATCC*gtaagtttaaacatgatattactaactaacaaagctcatttaaattttcag*CCGACTTCTTTAAGCAGTCCTTCCCCGAGGGCTTCACATGGGAGAGAGTCACCACATACGAAGACGGGGGCGTGCTGACCGCTACCCAGGACACCAGCCTCCAGGACGGCTGCCTCATCTACAACGTCAAGATCAGAGGGGTGAACTTCCCATCCAACGGCCCTGTGATGCAGAAGAAAACACTCGGCTGGGAGGCCTCCACCGAGACC*gttagttttaaatggcaatgtaccaatttaaagttttcaaacatgtttcag*CTGTACCCCGCTGACGGCGGCCTGGAAGGCAGAGCCGACATGGCCCTGAAGCTCGTGGGCGGGGGCCACCTGATCTGCAACTTGAAGACCACATACAGATCCAAGAAACCCGCTAAGAACCTCAAGATGCCCGGCGTCTACTATGTGGACAGAAGACTGGAAAGAATCAAGGAGGCCGACAAAGAGACCTACGTCGAGCAGCACGAGGTGGCTGTGGCCAGATACTGCGACCTCCCTAGCAAACTGGGGCACAGATAAgagctccgcatcggccgctgtcatcagatcgccatctcgcgcccgtgcctctgacttctaagtccaattactcttcaacatccctacatgctctttctccctgtgctcccaccccctatttttgttattatcaaaaaacttctcttaatttctttgttttttagcttcttttaagtcacctctaacaatgaaattgtgtagattcaaaaatagaattaattcgtaataaaaagtcgaaaaaaattgtgctccctccccccattaataataattctatcccaaaatctacacaatgttctgtgtacacttcttatgttttttacttctgataaatttttttgaaacatcatagaaaaaaccgcacacaaaataccttatcatatgttacgtttcagtttatgaccgcaatttttatttcttcgcacgtctgggcctctcatgacgtcaaatcatgctcatcgtgaaaaagttttggagtatttttggaatttttcaatcaagtgaaagtttatgaaattaattttcctgcttttgctttttggggtttcccctattgtttgtcaagatttcgaggacggcgtttttcttgctaaaatcacaagtattgatgagcacgatgcaagaaagatcggaagaaggtttgggtttgaggctcagtggaaggtgagtagaagttgataatttgaaagtggagtagtgtctatggggtttttgccttaaatgacagaatacattcccaatataccaaacataactgtttctactagtcggccgtacgggccctttcgtctcgcgcgtttcggtgatgacggtgaaaacctctgacacatgcagctcccggagacggtcacagcttgtctgtaagcggatgccgggagcagacaagcccgtcagggcgcgtcagcgggtgttggcgggtgtcggggctggcttaactatgcggcatcagagcagattgtactgagagtgcaccatatgcggtgtgaaataccgcacagatgcgtaaggagaaaataccgcatcaggcggccttaagggcctcgtgatacgcctatttttataggttaatgtcatgataataatggtttcttagacgtcaggtggcacttttcggggaaatgtgcgcggaacccctatttgtttatttttctaaatacattcaaatatgtatccgctcatgagacaataaccctgataaatgcttcaataatattgaaaaaggaagagtatgagtattcaacatttccgtgtcgcccttattcccttttttgcggcattttgccttcctgtttttgctcacccagaaacgctggtgaaagtaaaagatgctgaagatcagttgggtgcacgagtgggttacatcgaactggatctcaacagcggtaagatccttgagagttttcgccccgaagaacgttttccaatgatgagcacttttaaagttctgctatgtggcgcggtattatcccgtattgacgccgggcaagagcaactcggtcgccgcatacactattctcagaatgacttggttgagtactcaccagtcacagaaaagcatcttacggatggcatgacagtaagagaattatgcagtgctgccataaccatgagtgataacactgcggccaacttacttctgacaacgatcggaggaccgaaggagctaaccgcttttttgcacaacatgggggatcatgtaactcgccttgatcgttgggaaccggagctgaatgaagccataccaaacgacgagcgtgacaccacgatgcctgtagcaatggcaacaacgttgcgcaaactattaactggcgaactacttactctagcttcccggcaacaattaatagactggatggaggcggataaagttgcaggaccacttctgcgctcggcccttccggctggctggtttattgctgataaatctggagccggtgagcgtgggtctcgcggtatcattgcagcactggggccagatggtaagccctcccgtatcgtagttatctacacgacggggagtcaggcaactatggatgaacgaaatagacagatcgctgagataggtgcctcactgattaagcattggtaactgtcagaccaagtttactcatatatactttagattgatttaaaacttcatttttaatttaaaaggatctaggtgaagatcctttttgataatctcatgaccaaaatcccttaacgtgagttttcgttccactgagcgtcagaccccgtagaaaagatcaaaggatcttcttgagatcctttttttctgcgcgtaatctgctgcttgcaaacaaaaaaaccaccgctaccagcggtggtttgtttgccggatcaagagctaccaactctttttccgaaggtaactggcttcagcagagcgcagataccaaatactgttcttctagtgtagccgtagttaggccaccacttcaagaactctgtagcaccgcctacatacctcgctctgctaatcctgttaccagtggctgctgccagtggcgataagtcgtgtcttaccgggttggactcaagacgatagttaccggataaggcgcagcggtcgggctgaacggggggttcgtgcacacagcccagcttggagcgaacgacctacaccgaactgagatacctacagcgtgagctatgagaaagcgccacgcttcccgaagggagaaaggcggacaggtatccggtaagcggcagggtcggaacaggagagcgcacgagggagcttccagggggaaacgcctggtatctttatagtcctgtcgggtttcgccacctctgacttgagcgtcgatttttgtgatgctcgtcaggggggcggagcctatggaaaaacgccagcaacgcggcctttttacggttcctggccttttgctggccttttgctcacatgttctttcctgcgttatcccctgattctgtggataaccgtattaccgcctttgagtgagctgataccgctcgccgcagccgaacgaccgagcgcagcgagtcagtgagcgaggaagcggaagagcgcccaatacgcaaaccgcctctccccgcgcgttggccgattcattaatgcagctggcacgacaggtttcccgactggaaagcgggcagtgagcgcaacgcaattaatgtgagttagctcactcattaggcaccccaggctttacactttatgcttccggctcgtatgttgtgtggaattgtgagcggataacaatttcacacaggaaacagctatgaccatgattacgccaagct  ----- *nhx-2* promoter  ----- GFP coding region  *Italics*: introns  ----- linker region  ----- LGG-1 coding region  ----- mKate2 coding region  underlined text: *unc-54* 3’ UTR  GCA Gly116Ala |
