# Supplementary material for: A fluorescent reporter for rapid assessment of autophagic flux reveals unique autophagy signatures during C. elegans post-embryonic development and identifies compounds that modulate autophagy
Source: Autophagy Rep. 2024 Jul 11;3(1):2371736. doi: 10.1080/27694127.2024.2371736 (PMC11271720; doi:10.1080/27694127.2024.2371736)
Supplement: Supplementary information_Figs and Tables.pdf [file KAUO_A_2371736_SM2322.pdf]

A fluorescent reporter for rapid assessment of autophagic flux reveals unique autophagy signatures during C. elegans post-embryonic development and identifies compounds that modulate autophagy.

Zachary D. Dawson, Hemalatha Sundaramoorthi, Suk Regmi, Bo Zhang, Stephanie Morrison, Sara M. Fielder, Jessie R. Zhang, Hieu Hoang, David H. Perlmutter, Cliff J. Luke, Gary A. Silverman1, and Stephen C. Pak1*

SUPPLEMENTARY FIGURE LEGENDS

Figure S1. Differences in LGG-1 band patterns are not due to unequal protein loading. Stain-free image of gel used for LGG-1 Western blot in Fig. 1D showing similar total protein loading.

Figure S2. GFP does not form puncta in *epg-5(tm3425)*. Maximum intensity confocal images of (**A**) *nhx-2p::gfp* and (**B**) *nhx-2p::gfp; epg-5(tm3425)* animals showing diffuse cytoplasmic and nuclear GFP expression. GFP-positive puncta are absent in *nhx-2p::gfp; epg-5(tm3425)* animals. Compare with images in Fig. 2C. Day 1 adults are shown.

Figure S3. Expression of autophagic flux reporter (AFR) does not alter organismal fitness. (**A**) Brood size, (**B**) lifespan, and (**C**) grow rate through post-embryonic development of wild type (non-transgenic), *gfp::lgg-1::mKate2* (AFR) and *gfp::lgg-1* (DA2123) strains, respectively. Data are an average of three independent experiments. Lifespan data including mean survival times are provided in Table S3.

Figure S4. GFP::LGG-1 fluorescence decreases at a faster rate than mKate2 upon starvation. (**A**) GFP and (**B**) mKate2 fluorescence intensities per animal over 6 hours of starvation. The $t_{1/2}$ of GFP was ~1.7 hours. The $t_{1/2}$ of mKate2 was > 6 hours.

Figure S5. AFR(G116A) mutant control. (**A**) Maximum intensity confocal images of *nhx-2p::gfp::lgg-1::mKate2* (left) and *nhx-2p::gfp::lgg-1(G116A)::mKate2 control* (right). Day

1 adults are shown. (**B**) GFP puncta quantification. Shown are mean ± SD.

Figure S6. Representative well images of wild-type and autophagy mutants expressing the *gfp::lgg-1::mKate2* (AFR). All animals are day 1 adults.

Figure S7. Representative well images of wild-type, *let-363(ok3018)* and *rict-1(ft7)* mutant animals. *let-363(ok3018)* animals are homozygous lethal at the L3/L4 stage. As such, images were taken of young L3 animals (**A**). *rict-1(ft7)* mutants are homozygous viable. As such, images are of day 1 adults (**B**).

Figure S8. Representative well images of wild-type (left) and *atg-3(bp412)* (right) carrying the *nhx-2p::gfp::lgg-1::mKate2* transgene. L1 to D7 adult stages are shown. ~50 L1, ~50 L2, ~30 L3, ~20 L4, ~15 D1, D3, D5 and D7 stage animals are in each well.

Figure S9. Autophagic flux of *daf-2(e1370)* mutants. (**A**) GFP:mKate2 ratios of wild-type and *daf-2(e1370)* animals through postembryonic development at 15 ºC. (**B**) GFP:mKate2 ratios of wild-type L3 and *daf-2(e1370)* dauer animals cultured at 25 ºC as determined by confocal microscopy. GFP/mKate2 fluorescence was calculated using ImageJ. (**C**) Representative maximum intensity confocal images of animals in (**B**).

Figure S10. Pharyngeal pumping rates are lower in *eat-2(ad465)* mutants. Pharyngeal pumping rates of wild-type (blue) and *eat-2(ad465)* mutants (orange) at L3, L4 and D1 adult stages. Error bars represent mean ± SEM.

Figure S11. Eight-point dose response curves of nhx-2p::sGFP::ATZ expressing animals treated with (**A**) DMSO, (**B**) NVP-BGT226, (**C**) BAY 11-7082, (**D**) DMSO, and (**E**) SRT1720. Graphs are representative of three independent experiments. Error bars denote mean ± SEM.

Figure S12. Assessment of pharyngeal pumping rates and bacterial OD following drug treatment. (**A**) Pharyngeal pumping rates of L4 stage animals treated with hit compounds for 24 hours. All animals carried the *nhx-2p::gfp::lgg-1::mKate2* transgene. (B) OD_{600} of OP50 in wells

treated with DMSO or hit compounds for 24 hours.

Figure S13. Autophagic flux of *let-363(ok3018)* mutants. GFP:mKate2 ratios of wild-type and *let-363(ok3018)* animals at L1, L2 and L3 larval stages.

Table S1. Strains used in this study

Strain Name	Genotype
VK3785	*vkIs3785 [nhx-2p::gfp::lgg-1::mKate22]*
VK4081	*vkIs4046 [nhx-2p::gfp::lgg-1(G116A)::mKate2]*
VK3835	*vkIs3785 [nhx-2p::gfp::lgg-1::mKate2]; atg-3(bp412)*
VK3830	*vkIs3785 [nhx-2p::gfp::lgg-1::mKate2]; atg-4.1(bp501)*
VK3859	*vkIs3785 [nhx-2p::gfp::lgg-1::mKate2]; atg4.2(tm3948)*
VK3910	*vkIs3785 [nhx-2p::gfp::lgg-1::mKate2]; atg4.1(bp501)/tmC27; atg4.2(tm3948)*
VK4008	*vkIs3785 [nhx-2p::gfp::lgg-1::mKate2]; atg-5(bp484)*
VK3821	*vkIs3785 [nhx-2p::gfp::lgg-1::mKate2]; atg-9(bp564)*
VK4037	*vkIs3785 [nhx-2p::gfp::lgg-1::mKate2]; lgg-3 (tm1642)/hT2*
VK3824	*vkIs3785 [nhx-2p::gfp::lgg-1::mKate2]; atg-13(bp414)*
VK4023	*vkIs3785 [nhx-2p::gfp::lgg-1::mKate2]; daf-2(e1370)*
VK4049	*vkIs3785 [nhx-2p::gfp::lgg-1::mKate2]; daf-2(e1370); atg-3(bp412)]*
VK3818	*vkIs3785 [nhx-2p::gfp::lgg-1::mKate2]; epg-5(tm3425)*
VK3989	*vkIs3785 [nhx-2p::gfp::lgg-1::mKate2]; epg-8(bp251); him-5(e1490)*
VK4013	*vkIs3785 [nhx-2p::gfp::lgg-1::mKate2]; unc-51(e1189)*
DA2123	*adIs2122 [lgg-1::GFP + pRF4(rol-6)]*
VK4291	*vkIs3785 [nhx-2p::gfp::lgg-1::mKate2]; let-363(ok3018)*
VK4256	*vkIs3785 [nhx-2p::gfp::lgg-1::mKate2]; rict-1(ft7)*
VK4253	*vkIs3785 [nhx-2p::gfp::lgg-1::mKate2]; eat-2(ad465)*
VK2119	*vkIs2119 [nhx-2p::gfp]*
VK3569	*vkIs3569 [nhx-2p::gfp]; epg-5(tm3425)*

Table S2. List of all compounds in the autophagy library (Selleck Chemicals LLC) and their respective ratios

Normalized GFP/mKATE ratio	Compound Name	Catalog No.	CAS Number	Targets	Pathway
0.18	*BGT226 (NVP-BGT226)*	S2749	1245537-68-1	mTOR,PI3K	PI3K/Akt/mTOR
0.25	*Obatoclax Mesylate (GX15-070)*	S1057	803712-79-0	Autophagy,Bcl-2	Apoptosis
0.28	*Trifluoperazine 2HCl*	S3201	440-17-5	Autophagy	Ubiquitin
0.28	*Tamoxifen Citrate*	S1972	54965-24-1	Autophagy,Estrogen/progestogen Receptor	Endocrinology & Hormones
0.31	*Doxorubicin (Adriamycin)*	S1208	25316-40-9	Autophagy,Topoisomerase	DNA Damage
0.32	*BAY 11-7082*	S2913	19542-67-7	E2 conjugating,IκB/IKK	NF-κB
0.45	*SRT1720*	S1129	1001645-58-4	Sirtuin	Epigenetics
0.57	*CEP-18770 (Delanzomib)*	S1157	847499-27-8	Proteasome	Proteases
0.59	*DBeQ*	S7199	177355-84-9	p97	Ubiquitin
0.75	*Nilvadipine*	S2721	75530-68-6	Calcium Channel	Transmembrane Transporters
0.76	*NMS-873*	S7285	1418013-75-8	p97	Ubiquitin
0.76	*Trichostatin A (TSA)*	S1045	58880-19-6	HDAC	Epigenetics
0.77	*Isradipine*	S1662	75695-93-1	Calcium Channel	Transmembrane Transporters
0.80	*CCT137690*	S2744	1095382-05-0	Aurora Kinase	Cell Cycle
0.81	*Chrysophanic Acid*	S2406	481-74-3	EGFR,mTOR	Protein Tyrosine Kinase
0.82	*MLN2238*	S2180	1072833-77-2	Proteasome	Proteases
0.82	*GSK2126458 (GSK458)*	S2658	1086062-66-9	mTOR,PI3K	PI3K/Akt/mTOR
0.82	*Everolimus (RAD001)*	S1120	159351-69-6	mTOR	PI3K/Akt/mTOR
0.82	*CUDC-907*	S2759	1339928-25-4	PI3K,HDAC	Cytoskeletal Signaling
0.83	*Amiodarone HCl*	S1979	19774-82-4	Potassium Channel,Autophagy	Transmembrane Transporters
0.84	*Vincristine*	S1241	2068-78-2	Autophagy,Microtubule Associated	Cytoskeletal Signaling
0.84	*CI994 (Tacedinaline)*	S2818	112522-64-2	HDAC	Epigenetics
0.85	*Nimodipine*	S1747	66085-59-4	Autophagy,Calcium Channel	Transmembrane Transporters
0.86	*SGI-1776 free base*	S2198	1025065-69-3	Pim	JAK/STAT
0.86	*SNS-314 Mesylate*	S1154	1146618-41-8	Aurora Kinase	Cell Cycle
0.88	*Bortezomib (PS-341)*	S1013	179324-69-7	Proteasome	Proteases
0.88	*PHA-680632*	S1454	398493-79-3	Aurora Kinase	Cell Cycle
0.89	*Azithromycin*	S1835	83905-01-5	Autophagy	Ubiquitin
0.89	*Paclitaxel*	S1150	33069-62-4	Autophagy,Microtubule Associated	Cytoskeletal Signaling
0.89	*(-)-Parthenolide*	S2341	20554-84-1	E3 Ligase	Ubiquitin
0.89	*Nitrendipine*	S2491	39562-70-4	Calcium Channel,Autophagy	Transmembrane Transporters
0.90	*LAQ824 (Dacinostat)*	S1095	404951-53-7	HDAC	Epigenetics

0.90	*Aurora A Inhibitor I*	S1451	1158838-45-9	Aurora Kinase	Cell Cycle
0.90	*Manidipine 2HCl*	S2482	89226-75-5	Calcium Channel	Transmembrane Transporters
0.90	*WYE-125132 (WYE-132)*	S2661	1144068-46-1	mTOR	PI3K/Akt/mTOR
0.91	*Oprozomib (ONX 0912)*	S7049	935888-69-0	Proteasome	Proteases
0.91	*Amlodipine*	S1905	88150-42-9	Calcium Channel	Transmembrane Transporters
0.92	*Geldanamycin*	S2713	30562-34-6	HSP (e.g. HSP90),Autophagy	Cytoskeletal Signaling
0.93	*AZD8055*	S1555	1009298-09-2	mTOR	PI3K/Akt/mTOR
0.93	*PFI-1 (PF-6405761)*	S1216	1403764-72-6	Epigenetic Reader Domain	Epigenetics
0.94	*Nafamostat Mesylate*	S1386	82956-11-4	Serine Protease	Proteases
0.94	*VX-680 (Tozasertib, MK-0457)*	S1048	639089-54-6	Aurora Kinase	Cell Cycle
0.94	*Flunarizine 2HCl*	S2030	30484-77-6	Calcium Channel	Transmembrane Transporters
0.94	*Danusertib (PHA-739358)*	S1107	827318-97-8	FGFR,Aurora Kinase,c-RET,Bcr-Abl	Cell Cycle
0.94	*Azelnidipine*	S3053	123524-52-7	Calcium Channel	Transmembrane Transporters
0.94	*Torin 2*	S2817	1223001-51-1	mTOR,ATM/ATR	PI3K/Akt/mTOR
0.95	*ABT-737*	S1002	852808-04-9	Bcl-2,Autophagy	Apoptosis
0.95	*Pracinostat (SB939)*	S1515	929016-96-6	HDAC	Cytoskeletal Signaling
0.95	*Resveratrol*	S1396	501-36-0	Sirtuin,Autophagy	Epigenetics
0.95	*Thalidomide*	S1193	50-35-1	E3 Ligase ,TNF-alpha	Apoptosis
0.95	*PR-619*	S7130	2645-32-1	DUB	Ubiquitin
0.95	*WYE-354*	S1266	1062169-56-5	mTOR	PI3K/Akt/mTOR
0.97	*TCID*	S7140	30675-13-9	DUB	Ubiquitin
0.97	*Alisertib (MLN8237)*	S1133	1028486-01-2	Aurora Kinase	Cell Cycle
0.97	*Gemcitabine*	S1714	95058-81-4	DNA/RNA Synthesis,Autophagy	DNA Damage
0.97	*Loperamide HCl*	S2480	34552-83-5	Autophagy,Opioid Receptor	Neuronal Signaling
0.97	*Degrasyn (WP1130)*	S2243	856243-80-6	Bcr-Abl,DUB	Angiogenesis
0.97	*Cilnidipine*	S1293	132203-70-4	Calcium Channel	Transmembrane Transporters
0.97	*PCI-24781 (Abexinostat)*	S1090	783355-60-2	HDAC	Cytoskeletal Signaling
0.98	*3-Methyladenine*	S2767	5142-23-4	PI3K,Autophagy	PI3K/Akt/mTOR
0.98	*Forskolin*	S2449	66575-29-9	cAMP	GPCR & G Protein
0.98	*Brefeldin A*	S7046	20350-15-6	ATPase,Autophagy	Transmembrane Transporters
0.98	*GSK2578215A*	S7664	*1285515-21-0*	LRRK2	*Autophagy*
0.98	*IOX2*	S2919	931398-72-0	HIF	Angiogenesis
0.98	*Droxinostat*	S1422	99873-43-5	HDAC	Cytoskeletal Signaling
0.98	*Clevidipine Butyrate*	S2080	167221-71-8	Calcium Channel	Transmembrane Transporters
0.98	*Felodipine*	S1885	72509-76-3	Calcium Channel	Transmembrane Transporters
0.98	*Temsirolimus (CCI-779, NSC 68386[illegible]*	S1044	162635-04-3	mTOR	PI3K/Akt/mTOR

0.98	*Dexamethasone (DHAP)*	S1322	50-02-2	IL Receptor,Autophagy	Others
0.99	*GDC-0349*	S8040	1207360-89-1	mTOR	PI3K/Akt/mTOR
0.99	*Rapamycin (Sirolimus)*	S1039	53123-88-9	mTOR,Autophagy	PI3K/Akt/mTOR
0.99	GNE-7915	S7528	1351761-44-8	LRRK2	Autophagy
1.00	*Tetracaine HCl*	S2573	136-47-0	Calcium Channel	Transmembrane Transporters
1.00	*MK-5108 (VX-689)*	S2770	1010085-13-8	Aurora Kinase	Cell Cycle
1.00	*ONX-0914 (PR-957)*	S7172	960374-59-8	Proteasome	Proteases
1.00	*PYR-41*	S7129	418805-02-4	E1 Activating	Ubiquitin
1.00	*LY294002*	S1105	154447-36-6	Autophagy,PI3K	PI3K/Akt/mTOR
1.00	*RITA (NSC 652287)*	S2781	213261-59-7	E3 Ligase ,p53	Apoptosis
1.00	*Spautin-1*	S7888	*1262888-28-7*	Autophagy	*Autophagy*
1.00	*Wortmannin*	S2758	19545-26-7	PI3K,ATM/ATR,Autophagy	PI3K/Akt/mTOR
1.00	*P22077*	S7133	1247819-59-5	DUB	Ubiquitin
1.00	*Sulfacetamide Sodium*	S4081	127-56-0	Autophagy	Ubiquitin
1.01	*TAME*	S2225	901-47-3	APC,E3 Ligase	Cell Cycle
1.01	*Barasertib (AZD1152-HQPA)*	S1147	722544-51-6	Aurora Kinase	Cell Cycle
1.01	*JNJ-7706621*	S1249	443797-96-4	Aurora Kinase,CDK	Cell Cycle
1.01	*Manidipine*	S2481	89226-50-6	Calcium Channel	Transmembrane Transporters
1.01	*Rocilinostat (ACY-1215)*	S8001	1316214-52-4	HDAC	Epigenetics
1.01	*Givinostat (ITF2357)*	S2170	732302-99-7	HDAC	Cytoskeletal Signaling
1.02	*Valproic acid sodium salt (Sodium va*	S1168	1069-66-5	GABA Receptor,HDAC,Autophagy	Neuronal Signaling
1.02	*FG-4592*	S1007	808118-40-3	HIF	Angiogenesis
1.02	*MLN9708*	S2181	1201902-80-8	Proteasome	Proteases
1.02	*YM155 (Sepantronium Bromide)*	S1130	781661-94-7	Survivin	Apoptosis
1.02	*Hesperadin*	S1529	422513-13-1	Aurora Kinase	Cell Cycle
1.02	*KW-2449*	S2158	1000669-72-6	Bcr-Abl,Aurora Kinase,FLT3	Angiogenesis
1.03	*Carfilzomib (PR-171)*	S2853	868540-17-4	Proteasome	Proteases
1.03	*CYC116*	S1171	693228-63-6	VEGFR,Aurora Kinase	Cell Cycle
1.03	*JNJ-26854165 (Serdemetan)*	S1172	881202-45-5	E3 Ligase ,p53	Apoptosis
1.03	*Entinostat (MS-275)*	S1053	209783-80-2	HDAC	Epigenetics
1.03	*Clonidine HCl*	S2458	4205-91-8	Autophagy,Adrenergic Receptor	Neuronal Signaling
1.03	*ENMD-2076*	S1181	934353-76-1	Aurora Kinase,FLT3,VEGFR	Angiogenesis
1.05	*Vorinostat (SAHA, MK0683)*	S1047	149647-78-9	HDAC,Autophagy	Epigenetics
1.05	*Erlotinib HCl (OSI-744)*	S1023	183319-69-9	EGFR,Autophagy	Protein Tyrosine Kinase
1.05	*P5091 (P005091)*	S7132	882257-11-6	DUB	Ubiquitin
1.05	*Belinostat (PXD101)*	S1085	414864-00-9	HDAC	Epigenetics

1.05	*Mocetinostat (MGCD0103)*	S1122	726169-73-9	HDAC	Epigenetics
1.06	*Piperlongumine*	S7551	*20069-09-4*	Others	*Others*
1.06	*MG-132*	S2619	133407-82-6	Proteasome	Proteases
1.06	*Divalproex Sodium*	S1703	76584-70-8	Autophagy	Ubiquitin
1.06	*PP121*	S2622	1092788-83-4	PDGFR,mTOR,DNA-PK	Protein Tyrosine Kinase
1.06	*SBI-0206965*	S7885	*no CAS*	Autophagy	*Autophagy*
1.06	*Tubastatin A HCl*	S2627	1310693-92-5	HDAC	Epigenetics
1.06	*Aspirin*	S3017	50-78-2	COX	Proteases
1.06	GNE-0877	S7367	1374828-69-9	LRRK2	Autophagy
1.06	*Tenovin-1*	S8000	380315-80-0	E3 Ligase ,p53	Apoptosis
1.07	*TAK-901*	S2718	934541-31-8	Aurora Kinase	Cell Cycle
1.07	*Sodium Phenylbutyrate*	S4125	1716-12-7	HDAC	DNA Damage
1.07	*2-Methoxyestradiol (2-MeOE2)*	S1233	362-07-2	HIF	Angiogenesis
1.07	*CCT129202*	S1519	942947-93-5	Aurora Kinase	Cell Cycle
1.07	*Nocodazole*	S2775	31430-18-9	Autophagy,Microtubule Associated	Cytoskeletal Signaling
1.07	*PCI-34051*	S2012	950762-95-5	HDAC	Epigenetics
1.07	*PP242*	S2218	1092351-67-1	mTOR,Autophagy	PI3K/Akt/mTOR
1.07	*OSI-027*	S2624	936890-98-1	mTOR	PI3K/Akt/mTOR
1.07	*AT9283*	S1134	896466-04-9	Aurora Kinase,JAK,Bcr-Abl	JAK/STAT
1.07	*PF-04691502*	S2743	1013101-36-4	mTOR,Akt,PI3K	PI3K/Akt/mTOR
1.07	*Nutlin-3*	S1061	890090-75-2	E3 Ligase ,Mdm2	Apoptosis
1.07	GNE-9605	S7368	1536200-31-3	LRRK2	Autophagy
1.07	*IU1*	S7134	314245-33-5	DUB	Proteases
1.08	*Lacidipine*	S1994	103890-78-4	Calcium Channel	Transmembrane Transporters
1.08	*MLN8054*	S1100	869363-13-3	Aurora Kinase	Cell Cycle
1.08	*MHY1485*	S7811	*326914-06-1*	mTOR,Autophagy	*PI3K/Akt/mTOR*
1.08	*Pifithrin-α (PFTα)*	S2929	63208-82-2	Autophagy,p53	Apoptosis
1.09	*Carbamazepine*	S1693	298-46-4	Sodium Channel,Autophagy	Transmembrane Transporters
1.09	*AMG-900*	S2719	945595-80-2	Aurora Kinase	Cell Cycle
1.09	*Temozolomide*	S1237	85622-93-1	Autophagy	Ubiquitin
1.09	*M344*	S2779	251456-60-7	HDAC	Cytoskeletal Signaling
1.09	*EX 527 (Selisistat)*	S1541	49843-98-3	Sirtuin	Epigenetics
1.10	*Scriptaid*	S8043	287383-59-9	HDAC	DNA Damage
1.10	*Y-27632 2HCl*	S1049	129830-38-2	ROCK,Autophagy	Cell Cycle
1.10	*Fasudil (HA-1077) HCl*	S1573	105628-07-7	ROCK,Autophagy	Cell Cycle
1.11	*Rotundine*	S2437	483-14-7	Dopamine Receptor	Neuronal Signaling

1.11	*LDN-57444*	S7135	668467-91-2	DUB	Proteases
1.11	*Bupivacaine HCl*	S2454	18010-40-7	Sodium Channel	GPCR & G Protein
1.11	*SAR245409 (XL765)*	S1523	1349796-36-6	PI3K,mTOR	PI3K/Akt/mTOR
1.11	*ZM 447439*	S1103	331771-20-1	Aurora Kinase	Cell Cycle
1.11	*PI-103*	S1038	371935-74-9	PI3K,DNA-PK,Autophagy,mTOR	PI3K/Akt/mTOR
1.11	*CUDC-101*	S1194	1012054-59-9	EGFR,HER2,HDAC	Epigenetics
1.13	*KU-0063794*	S1226	938440-64-3	mTOR	PI3K/Akt/mTOR
1.14	*Quisinostat (JNJ-26481585)*	S1096	875320-29-9	HDAC	Epigenetics
1.14	*Omeprazole*	S1389	73590-58-6	Autophagy,Proton Pump	Transmembrane Transporters
1.14	*Gabexate Mesylate*	S2101	56974-61-9	Serine Protease	Proteases
1.14	*SMI-4a*	S8005	438190-29-5	Pim	JAK/STAT
1.15	*Ranolazine 2HCl*	S1425	95635-56-6	Calcium Channel	Transmembrane Transporters
1.57	*MC1568*	S1484	852475-26-4	HDAC	Cytoskeletal Signaling
3.34	*Sirtinol*	S2804	410536-97-9	Sirtuin	Epigenetics

Table S3. Lifespan analyses

Strain	Mean +/-SEM (days)	75th percentile (days)	*P* values	n	Fig.
**Autophagic Flux Reporter (AFR) has comparable lifespan to wild-type VC2010 animals**					
wild-type (VC2010)	18.71 +/- 0.64	22		79/90	Fig. S3B
gfp::lgg-1::mKate2 (VK3785)	18.28 +/- 0.62	22	0.6117	80/90	
gfp::lgg-1 (DA2123)	15.91 +/- 0.63	18	0.0043	68/90	

Table S4. Maps and sequences of AFR and AFR(G116A) constructs

Wild-type AFR construct

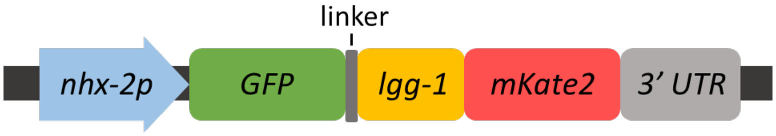

tccttccaagttattgacaagtattaactgcacttctcataaaaatttttcaaatagatctatttagtctggaataggaatacttttggcaattttcttgag
aatcatagaagacgaatttctgaactgctctttgtgaagccacgcccatatattggcattttgtatccaatttttgtagagtacctgaatcattgtcact
acatctgctaaacaatatatggaagttctatgagaggtgaaaggtaaatcatataacaactgtaataacatagaaatgattgtagtatatttccaatt
aggctgagttgatcttattgaagagtttgaatcatagcaatttttcctcttttagtatttttcaaaacaaattttcacaatgttagatgtagtatttagcatt
aaggcatctgtaatctcccagaaatgttttgggaataaaccctttgactctttggtcctttttgctccaaataatatctgattttaacccgttctgatcta
ctttttacctttcgatcgtcacatccgcgtacacgtttctcttgctctcaattgcttatctttatggccttttcccccactttttgtcgactatcatgcggacgt
ggcttccttgctccctcccggtttttttgaaaacacttgtgaatttatttttgtggtcaaaattacgaaacatttgaattctcaatctatttatagaccgggt
gtggtatgttatgccatgtttttgtcattgccaaatgaacatcgatattttaattttctgaacttaccgtacccccctgcgttactattttatttttaatttgtat
aatttcattgctataatttgttgtccttgaaacattgaagatatcgaagaaataatgacaaaaatcagatagaacaatttttcctactcatcaactcaca
tttttctatttctcttgtgcgaaaaaaatagtatattgggaatatttatagtattgcaacttttctcttagttcgcagagttgcaatactactagatcccta
atagaggaaatatggtgaccagctttttgcaattggaaagttgggaaaaaagtatatatattaatagacttacattcttgtagttcgtagaatttttcag
gaaatctgacacgcaagacttttcaaaaagtttttttttttaatttggcaatctgccaaaacgtttactgaacattttgaaaacatctaccagtaccttta
acacaacgaaaaattgttctaagcaatattaactgtgataattgatgttaagacctgaaatttaatcacaaatttcacctggaaatgacagtaatcgg
cttctaaatactgtcgacacaagtgccatacctaagattccaatcctattaccatgtttcccaatttcattctctttcgtatctctatttctcttctttattatc
aattactttttatcagttcttcgtttgctttaacatcaagagcacatagcgctctctcgtctctctctcatgctcttttgaacttttacaaaaaactatttcc
ggttgttttgattcttggaatttgaaataattttcagtgattaaatctagaggatccccgggattggccaaaggacccaaag*gtatgtttcgaatgata*
*ctaacataacatagaacattttcag*gaggacccttggctagcctcgagATGAGTAAAGGAGAAGAACTTTTCACTGGAGTTGTCC
CAATTCTTGTTGAATTAGATGGTGATGTTAATGGGCACAAATTTTCTGTCAGTGGAGAGGGTGAAGGTGATGCAA
CATACGGAAAACTTACCCTTAAATTTATTTGCACTACTGGAAAACTACCTGTTCCATGG*gtaagtttaaacatatatatact*
*aactaaccctgattatttaaattttcag*CCAACACTTGTCACTACTTTCTGTTATGGTGTTCAATGCTTTTCAAGATACCCAGA
TCATATGAAACGGCATGACTTTTTCAAGAGTGCCATGCCCGAAGGTTATGTACAGGAAAGAACTATATTTTTCAAA
GATGACGGGAACTACAAGACAC*gtaagtttaaacagttcggtactaactaaccatacatatttaaattttcag*GTGCTGAAGTCAA
GTTTGAAGGTGATACCCTTGTTAATAGAATCGAGTTAAAAGGTATTGATTTTAAAGAAGATGGAAACATTCTTGGA
CACAAATTGGAATACAACTATAACTCACACAATGTATACATCATGGCAGACAAACAAAAGAATGGAATCAAAGTT*gt*
*aagtttaaacatgattttactaactaactaatctgatttaaattttcag*AACTTCAAAATTAGACACAACATTGAAGATGGAAGCG
TTCAACTAGCAGACCATTATCAACAAAATACTCCAATTGGCGATGGCCCTGTCCTTTTACCAGACAACCATTACCTG
TCCACACAATCTGCCCTTTCGAAAGATCCCAACGAAAAGAGAGACCACATGGTCCTTCTTGAGTTTGTAACAGCTG
CTGGGATTACACATGGCATGGATGAACTATACAAAGGTGGCGGTGGCTCGGGCGGTGGTGGGTCGGGTGGCGG
CGGAATGAAGTGGGCTTACAAGGAGGAGAACAACTTTGAGAAGCGTCGTGCCGAAGGAGACAAGATCCGCAG
AAAGTACCCAGACCGTATTCCAGTGATTGTTGAGAAAGCACCAAAGTCAAAGCTCCATGACTTGGATAAGAAGAA
GTACTTGGTCCCATCCGATCTTACTGTTGGACAGTTCTACTTCCTCATCAGAAAACGCATCCAACTTCGTCCAGAAG
ATGCTCTGTTCTTCTTTGTCAACAATGTCATTCCACAAACCATGACCACAATGGGACAACTCTACCAG*gtaactaacca*
*gtcgtttattttcatttaattaacccttttctttattacag*GACCATCACGAGGAAGACTTGTTCCTTTACATCGCCTACAGTGACG
AAAGTGTGTATGGAGGAGAGGTCGAAAAGAAGGAAATGGTGAGCGAGCTGATTAAGGAGAACATGCACATGAA
GCTGTACATGGAGGGCACCGTGAACAACCACCACTTCAAGTGCACATCCGAGGGCGAAGGCAAGCCCTACGAG
GGCACCCAGACCATGAGAATCAAGGCGGTCGAGGGCGGCCCTCTCCCCTTCGCCTTCGACATCCTGGCTACCAGC
TTCATGTACGGCAGCAAAACCTTCATCAACCACACCCAGGGCATCC*gtaagtttaaacatgatattactaactaacaaagctc*
*atttaaattttcag*CCGACTTCTTTAAGCAGTCCTTCCCCGAGGGCTTCACATGGGAGAGAGTCACCACATACGAAGAC
GGGGGCGTGCTGACCGCTACCCAGGACACCAGCCTCCAGGACGGCTGCCTCATCTACAACGTCAAGATCAGAGG

GGTGAACTTCCCATCCAACGGCCCTGTGATGCAGAAGAAAACACTCGGCTGGGAGGCCTCCACCGAGACC*gttag*
*ttttaaatggcaatgtaccaatttaaagttttcaaacatgtttcag*CTGTACCCCGCTGACGGCGGCCTGGAAGGCAGAGCCGAC
ATGGCCCTGAAGCTCGTGGGCGGGGGCCACCTGATCTGCAACTTGAAGACCACATACAGATCCAAGAAACCCGC
TAAGAACCTCAAGATGCCCGGCGTCTACTATGTGGACAGAAGACTGGAAAGAATCAAGGAGGCCGACAAAGAG
ACCTACGTCGAGCAGCACGAGGTGGCTGTGGCCAGATACTGCGACCTCCCTAGCAAACTGGGGCACAGATAAgag
ctccgcatcggccgctgtcatcagatcgccatctcgcgcccgtgcctctgacttctaagtccaattactcttcaacatccctacatgctctttctccctgtg
ctcccacccccctatttttgttattatcaaaaaacttctcttaatttctttgtttttttagcttcttttaagtcacctctaacaatgaaattgtgtagattcaaaaa
tagaattaattcgtaataaaaagtcgaaaaaaattgtgctccctccccccattaataataattctatcccaaaatctacacaatgttctgtgtacacttc
ttatgtttttttacttctgataaatttttttgaaacatcatagaaaaaaccgcacacaaaataccttatcatatgttacgtttcagtttatgaccgcaattttt
atttcttcgcacgtctgggcctctcatgacgtcaaatcatgctcatcgtgaaaaagttttggagtatttttggaatttttcaatcaagtgaaagtttatgaa
attaattttcctgcttttgctttttgggggtttcccctattgtttgtcaagatttcgaggacggcgtttttcttgctaaaatcacaagtattgatgagcacgatg
caagaaagatcggaagaaggtttgggtttgaggctcagtggaaggtgagtagaagttgataatttgaaagtggagtagtgtctatggggtttttgcct
taaatgacagaatacattcccaatataccaaacataactgtttctactagtcggccgtacgggccctttcgtctcgcgcgtttcggtgatgacggtgaa
aacctctgacacatgcagctcccggagacggtcacagcttgtctgtaagcggatgccgggagcagacaagcccgtcagggcgcgtcagcgggtgtt
ggcgggtgtcggggctggcttaactatgcggcatcagagcagattgtactgagagtgcaccatatgcggtgtgaaataccgcacagatgcgtaagg
agaaaataccgcatcaggcggccttaagggcctcgtgatacgcctatttttataggttaatgtcatgataataatggtttcttagacgtcaggtggcac
ttttcggggaaatgtgcgcggaacccctatttgtttatttttctaaatacattcaaatatgtatccgctcatgagacaataaccctgataaatgcttcaat
aatattgaaaaaggaagagtatgagtattcaacatttccgtgtcgcccttattcccttttttgcggcattttgccttcctgtttttgctcacccagaaacgc
tggtgaaagtaaaagatgctgaagatcagttgggtgcacgagtgggttacatcgaactggatctcaacagcggtaagatccttgagagttttcgccc
cgaagaacgttttccaatgatgagcacttttaaagttctgctatgtggcgcggtattatcccgtattgacgccgggcaagagcaactcggtcgccgcat
acactattctcagaatgacttggttgagtactcaccagtcacagaaaagcatcttacggatggcatgacagtaagagaattatgcagtgctgccataa
ccatgagtgataacactgcggccaacttacttctgacaacgatcggaggaccgaaggagctaaccgcttttttgcacaacatgggggatcatgtaac
tcgccttgatcgttgggaaccggagctgaatgaagccataccaaacgacgagcgtgacaccacgatgcctgtagcaatggcaacaacgttgcgcaa
actattaactggcgaactacttactctagcttcccggcaacaattaatagactggatggaggcggataaagttgcaggaccacttctgcgctcggccc
ttccggctggctggtttattgctgataaatctggagccggtgagcgtgggtctcgcggtatcattgcagcactggggccagatggtaagccctcccgt
atcgtagttatctacacgacggggagtcaggcaactatggatgaacgaaatagacagatcgctgagataggtgcctcactgattaagcattggtaac
tgtcagaccaagtttactcatatatactttagattgatttaaaacttcatttttaatttaaaaggatctaggtgaagatcctttttgataatctcatgacca
aaatcccttaacgtgagttttcgttccactgagcgtcagaccccgtagaaaagatcaaaggatcttcttgagatcctttttttctgcgcgtaatctgctgc
ttgcaaacaaaaaaaccaccgctaccagcggtggtttgtttgccggatcaagagctaccaactctttttccgaaggtaactggcttcagcagagcgc
agataccaaatactgttcttctagtgtagccgtagttaggccaccacttcaagaactctgtagcaccgcctacatacctcgctctgctaatcctgttacc
agtggctgctgccagtggcgataagtcgtgtcttaccgggttggactcaagacgatagttaccggataaggcgcagcggtcgggctgaacgggggg
ttcgtgcacacagcccagcttggagcgaacgacctacaccgaactgagatacctacagcgtgagctatgagaaagcgccacgcttcccgaaggga
gaaaggcggacaggtatccggtaagcggcagggtcggaacaggagagcgcacgagggagcttccagggggaaacgcctggtatctttatagtcc
tgtcgggtttcgccacctctgacttgagcgtcgatttttgtgatgctcgtcaggggggcggagcctatggaaaaacgccagcaacgcggcctttttacg
gttcctggccttttgctggccttttgctcacatgttctttcctgcgttatcccctgattctgtggataaccgtattaccgcctttgagtgagctgataccgctc
gccgcagccgaacgaccgagcgcagcgagtcagtgagcgaggaagcggaagagcgcccaatacgcaaaccgcctctccccgcgcgttggccgat
tcattaatgcagctggcacgacaggtttcccgactggaaagcgggcagtgagcgcaacgcaattaatgtgagttagctcactcattaggcaccccag
gctttacactttatgcttccggctcgtatgttgtgtggaattgtgagcggataacaatttcacacaggaaacagctatgaccatgattacgccaagct

----- *nhx-2* promoter
----- GFP coding region
Italics: introns
----- linker region
----- LGG-1 coding region
----- mKate2 coding region
underlined text: *unc-54* 3’ UTR

AFR(G116A) construct

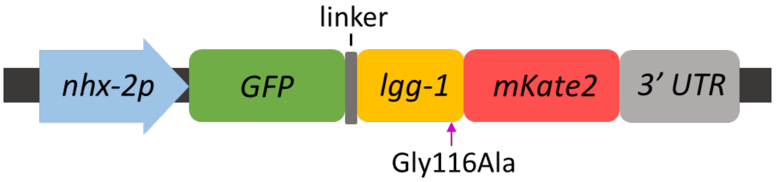

tccttccaagttattgacaagtattaactgcacttctcataaaaatttttcaaatagatctatttagtctggaataggaatactttggcaattttcttgag
aatcatagaagacgaatttctgaactgctctttgtgaagccacgcccatatattggcatttttgtatccaatttttgtagagtacctgaatcattgtcact
acatctgctaaacaatatatggaagttctatgagaggtgaaaggtaaatcatataacaactgtaataacatagaaatgattgtagtatatttccaatt
aggctgagttgatcttattgaagagtttgaatcatagcaatttttcctcttttagtatttttcaaaacaaattttcacaatgttagatgtagtatttagcatt
aaggcatctgtaatctcccagaaatgttttgggaataaaaccctttgactctttggtcctttttttgctccaaataatatctgattttaacccgttctgatcta
ctttttacctttcgatcgtcacatccgcgtacacgtttctcttgctctcaattgcttatctttatggccttttcccccactttttgtcgactatcatgcggacgt
ggcttccttgctccctcccggttttttgaaaacacttgtgaatttatttttgtggtcaaaattacgaaacatttgaattctcaatctatttatagaccgggt
gtggtatgttatgccatgtttttgtcattgccaaatgaacatcgatattttaattttctgaacttaccgtaccccctgcgttactatttatttttaatttgtat
aatttcattgctataatttgttgtccttgaaacattgaagatatcgaagaaataatgacaaaaatcagatagaacaatttttcctactcatcaactcaca
tttttctattttctcttgtgcgaaaaaaaatagtatattgggaatatttatagtattgcaacttttctcttagttcgcagagttgcaatactactagatcccta
atagaggaaatatggtgaccagctttttgcaattggaaagttgggaaaaaagtatatatattaatagacttacattcttgtagttcgtagaatttttcag
gaaatctgacacgcaagacttttttcaaaaagtttttttttttaatttggcaatctgccaaaacgtttactgaacattttgaaaacatctaccagtaccttta
acacaacgaaaaattgttctaagcaatattaactgtgataattgatgttaagacctgaaatttaatcacaaatttcacctggaaatgacagtaatcgg
cttctaaatactgtcgacacaagtgccatacctaagattccaatcctattaccatgtttcccaatttcattctctttcgtatctctatttctcttctttattatc
aattactttttatcagttcttcgtttgctttaacatcaagagcacatagcgctctctcgtctctctctcatgctcttttttgaacttttacaaaaaaactatttcc
ggttgttttgattcttggaatttgaaataattttcagtgattaaatctagaggatccccgggattggccaaaggacccaaag*gtatgtttcgaatgata*
*ctaacataacatagaacattttcag*gaggacccttggctagcctcgagATGAGTAAAGGAGAAGAACTTTTCACTGGAGTTGTCC
CAATTCTTGTTGAATTAGATGGTGATGTTAATGGGCACAAATTTTCTGTCAGTGGAGAGGGTGAAGGTGATGCAA
CATACGGAAAACTTACCCTTAAATTTATTTGCACTACTGGAAAACTACCTGTTCCATGG*gtaagtttaaacatatatatact*
*aactaaccctgattatttaaattttcag*CCAACACTTGTCACTACTTTCTGTTATGGTGTTCAATGCTTTTCAAGATACCCAGA
TCATATGAAACGGCATGACTTTTTCAAGAGTGCCATGCCCGAAGGTTATGTACAGGAAAGAACTATATTTTTCAAA
GATGACGGGAACTACAAGACAC*gtaagtttaaacagttcggtactaactaaccatacatatttaaattttcag*GTGCTGAAGTCAA
GTTTGAAGGTGATACCCTTGTTAATAGAATCGAGTTAAAAGGTATTGATTTTAAAGAAGATGGAAACATTCTTGGA
CACAAATTGGAATACAACTATAACTCACACAATGTATACATCATGGCAGACAAACAAAAGAATGGAATCAAAGTT*gt*
*aagtttaaacatgattttactaactaactaatctgatttaaattttcag*AACTTCAAAATTAGACACAACATTGAAGATGGAAGCG
TTCAACTAGCAGACCATTATCAACAAAATACTCCAATTGGCGATGGCCCTGTCCTTTTACCAGACAACCATTACCTG
TCCACACAATCTGCCCTTTCGAAAGATCCCAACGAAAAGAGAGACCACATGGTCCTTCTTGAGTTTGTAACAGCTG
CTGGGATTACACATGGCATGGATGAACTATACAAAGGTGGCGGTGGCTCGGGCGGTGGTGGGTCGGGTGGCGG
CGGAATGAAGTGGGCTTACAAGGAGGAGAACAACTTTGAGAAGCGTCGTGCCGAAGGAGACAAGATCCGCAG
AAAGTACCCAGACCGTATTCCAGTGATTGTTGAGAAAGCACCAAAGTCAAAGCTCCATGACTTGGATAAGAAGAA
GTACTTGGTCCCATCCGATCTTACTGTTGGACAGTTCTACTTCCTCATCAGAAAACGCATCCAACTTCGTCCAGAAG
ATGCTCTGTTCTTCTTTGTCAACAATGTCATTCCACAAACCATGACCACAATGGGACAACTCTACCAG*gtaactaacca*
*gtcgtttattttcatttaattaacccttttctttattacag*GACCATCACGAGGAAGACTTGTTCCTTTACATCGCCTACAGTGACG
AAAGTGTGTATGCAGGAGAGGTCGAAAAGAAGGAAATGGTGAGCGAGCTGATTAAGGAGAACATGCACATGAA
GCTGTACATGGAGGGCACCGTGAACAACCACCACTTCAAGTGCACATCCGAGGGCGAAGGCAAGCCCTACGAG
GGCACCCAGACCATGAGAATCAAGGCGGTCGAGGGCGGCCCTCTCCCCTTCGCCTTCGACATCCTGGCTACCAGC
TTCATGTACGGCAGCAAAACCTTCATCAACCACACCCAGGGCATCC*gtaagtttaaacatgatattactaactaacaaagctc*
*atttaaattttcag*CCGACTTCTTTAAGCAGTCCTTCCCCGAGGGCTTCACATGGGAGAGAGTCACCACATACGAAGAC

GGGGGCGTGCTGACCGCTACCCAGGACACCAGCCTCCAGGACGGCTGCCTCATCTACAACGTCAAGATCAGAGG
GGTGAACTTCCCATCCAACGGCCCTGTGATGCAGAAGAAAACACTCGGCTGGGAGGCCTCCACCGAGACC*gttag*
*ttttaaatggcaatgtaccaatttaaagttttcaaacatgtttcag*CTGTACCCCGCTGACGGCGGCCTGGAAGGCAGAGCCGAC
ATGGCCCTGAAGCTCGTGGGCGGGGGCCACCTGATCTGCAACTTGAAGACCACATACAGATCCAAGAAACCCGC
TAAGAACCTCAAGATGCCCGGCGTCTACTATGTGGACAGAAGACTGGAAAGAATCAAGGAGGCCGACAAAGAG
ACCTACGTCGAGCAGCACGAGGTGGCTGTGGCCAGATACTGCGACCTCCCTAGCAAACTGGGGCACAGATAAgag
ctccgcatcggccgctgtcatcagatcgccatctcgcgcccgtgcctctgacttctaagtccaattactcttcaacatccctacatgctctttctccctgtg
ctcccacccctattttgttattatcaaaaaacttctcttaatttctttgtttttagcttcttttaagtcacctctaacaatgaaattgtgtagattcaaaaa
tagaattaattcgtaataaaaagtcgaaaaaaattgtgctccctccccccattaataataattctatcccaaaatctacacaatgttctgtgtacacttc
ttatgtttttacttctgataaattttttgaaacatcatagaaaaaaccgcacacaaaataccttatcatatgttacgtttcagtttatgaccgcaattttt
atttcttcgcacgtctgggcctctcatgacgtcaaatcatgctcatcgtgaaaaagttttggagtatttttggaatttttcaatcaagtgaaagtttatgaa
attaattttcctgcttttgctttttggggtttcccctattgtttgtcaagatttcgaggacggcgtttttcttgctaaaatcacaagtattgatgagcacgatg
caagaaagatcggaagaaggtttgggtttgaggctcagtggaaggtgagtagaagttgataatttgaaagtggagtagtgtctatggggtttttgcct
taaatgacagaatacattcccaatataccaaacataactgtttctactagtcggccgtacgggccctttcgtctcgcgcgtttcggtgatgacggtgaa
aacctctgacacatgcagctcccggagacggtcacagcttgtctgtaagcggatgccgggagcagacaagcccgtcagggcgcgtcagcgggtgtt
ggcgggtgtcggggctggcttaactatgcggcatcagagcagattgtactgagagtgcaccatatgcggtgtgaaataccgcacagatgcgtaagg
agaaaataccgcatcaggcggccttaagggcctcgtgatacgcctatttttataggttaatgtcatgataataatggtttcttagacgtcaggtggcac
ttttcggggaaatgtgcgcggaacccctatttgtttatttttctaaatacattcaaatatgtatccgctcatgagacaataaccctgataaatgcttcaat
aatattgaaaaaggaagagtatgagtattcaacatttccgtgtcgcccttattcccttttttgcggcattttgccttcctgtttttgctcacccagaaacgc
tggtgaaagtaaaagatgctgaagatcagttgggtgcacgagtgggttacatcgaactggatctcaacagcggtaagatccttgagagttttcgccc
cgaagaacgttttccaatgatgagcacttttaaagttctgctatgtggcgcggtattatcccgtattgacgccgggcaagagcaactcggtcgccgcat
acactattctcagaatgacttggttgagtactcaccagtcacagaaaagcatcttacggatggcatgacagtaagagaattatgcagtgctgccataa
ccatgagtgataacactgcggccaacttacttctgacaacgatcggaggaccgaaggagctaaccgcttttttgcacaacatgggggatcatgtaac
tcgccttgatcgttgggaaccggagctgaatgaagccataccaaacgacgagcgtgacaccacgatgcctgtagcaatggcaacaacgttgcgcaa
actattaactggcgaactacttactctagcttcccggcaacaattaatagactggatggaggcggataaagttgcaggaccacttctgcgctcggccc
ttccggctggctggtttattgctgataaatctggagccggtgagcgtgggtctcgcggtatcattgcagcactggggccagatggtaagccctcccgt
atcgtagttatctacacgacggggagtcaggcaactatggatgaacgaaatagacagatcgctgagataggtgcctcactgattaagcattggtaac
tgtcagaccaagtttactcatatatactttagattgatttaaaacttcatttttaatttaaaaggatctaggtgaagatcctttttgataatctcatgacca
aaatcccttaacgtgagttttcgttccactgagcgtcagaccccgtagaaaagatcaaaggatcttcttgagatcctttttttctgcgcgtaatctgctgc
ttgcaaacaaaaaaaccaccgctaccagcggtggtttgtttgccggatcaagagctaccaactctttttccgaaggtaactggcttcagcagagcgc
agataccaaatactgttcttctagtgtagccgtagttaggccaccacttcaagaactctgtagcaccgcctacatacctcgctctgctaatcctgttacc
agtggctgctgccagtggcgataagtcgtgtcttaccgggttggactcaagacgatagttaccggataaggcgcagcggtcgggctgaacgggggg
ttcgtgcacacagcccagcttggagcgaacgacctacaccgaactgagatacctacagcgtgagctatgagaaagcgccacgcttcccgaaggga
gaaaggcggacaggtatccggtaagcggcagggtcggaacaggagagcgcacgagggagcttccagggggaaacgcctggtatctttatagtcc
tgtcgggtttcgccacctctgacttgagcgtcgatttttgtgatgctcgtcaggggggcggagcctatggaaaaacgccagcaacgcggcctttttacg
gttcctggccttttgctggccttttgctcacatgttctttcctgcgttatcccctgattctgtggataaccgtattaccgcctttgagtgagctgataccgctc
gccgcagccgaacgaccgagcgcagcgagtcagtgagcgaggaagcggaagagcgcccaatacgcaaaccgcctctccccgcgcgttggccgat
tcattaatgcagctggcacgacaggtttcccgactggaaagcgggcagtgagcgcaacgcaattaatgtgagttagctcactcattaggcaccccag
gctttacactttatgcttccggctcgtatgttgtgtggaattgtgagcggataacaatttcacacaggaaacagctatgaccatgattacgccaagct

----- *nhx-2* promoter
----- GFP coding region
Italics: introns
----- linker region
----- LGG-1 coding region
----- mKate2 coding region
underlined text: *unc-54* 3’ UTR

GCA Gly116Ala

Figure S1

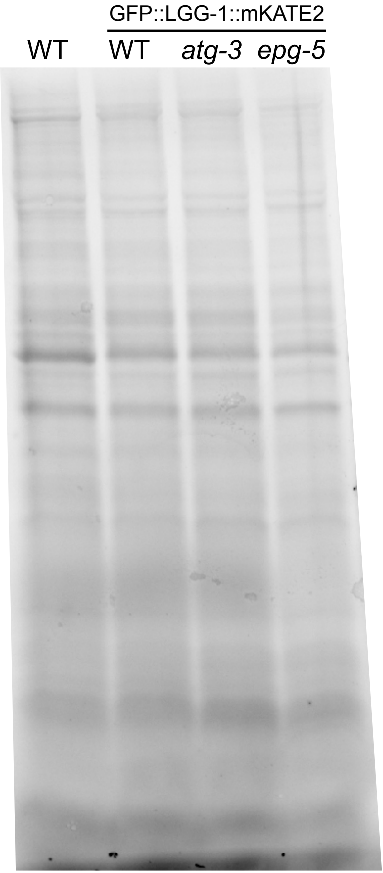

Stain-free gel

Figure S2

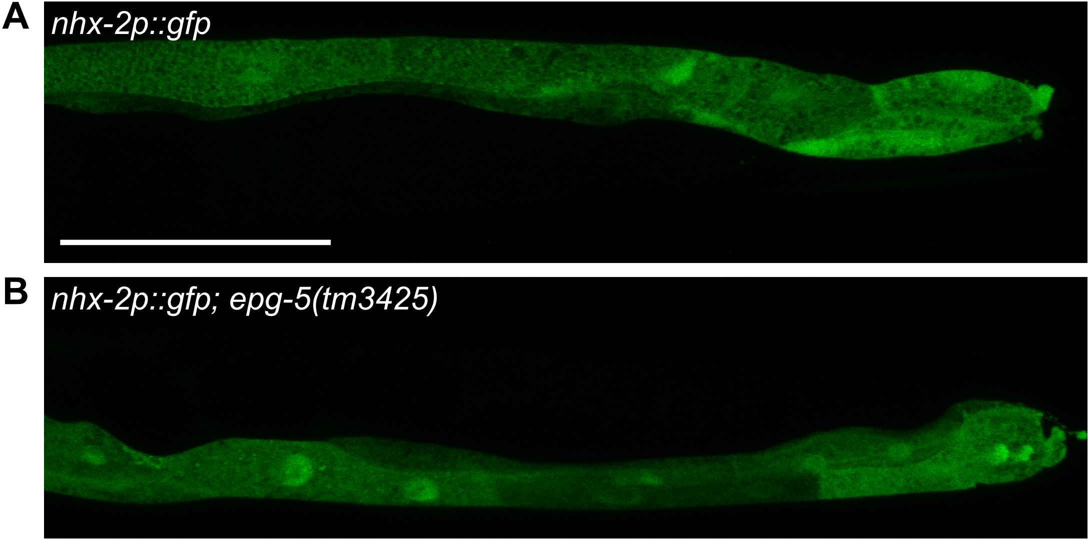

Figure S3

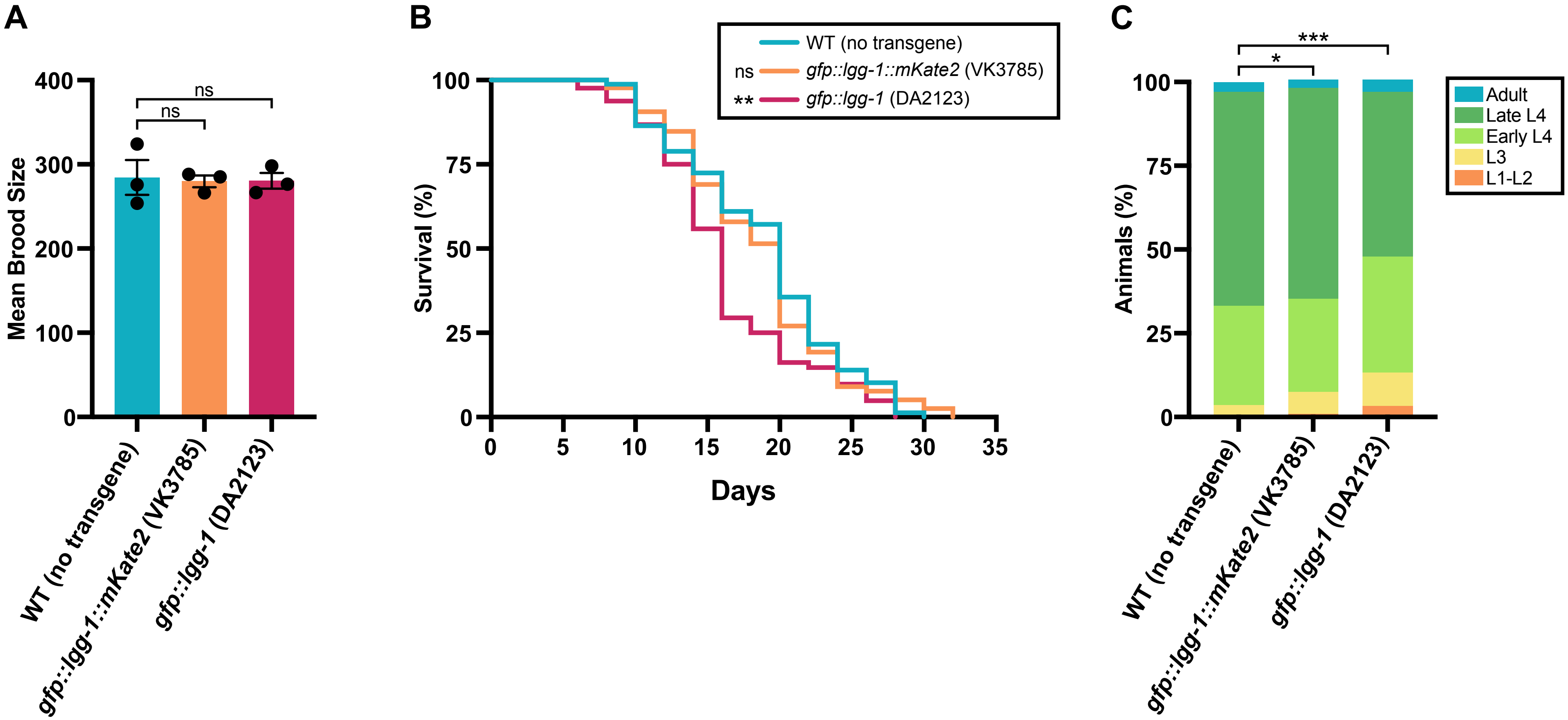
A
Mean Brood Size
400
300
200
100
0
ns
ns
WT (no transgene)
gfp::lgg-1::mKate2 (VK3785)
gfp::lgg-1 (DA2123)
B
WT (no transgene)
ns gfp::lgg-1::mKate2 (VK3785)
** gfp::lgg-1 (DA2123)
Survival (%)
100
75
50
25
0
0
5
10
15
20
25
30
35
Days
C
***
*
Animals (%)
100
75
50
25
0
Adult
Late L4
Early L4
L3
L1-L2
WT (no transgene)
gfp::lgg-1::mKate2 (VK3785)
gfp::lgg-1 (DA2123)

Figure S4

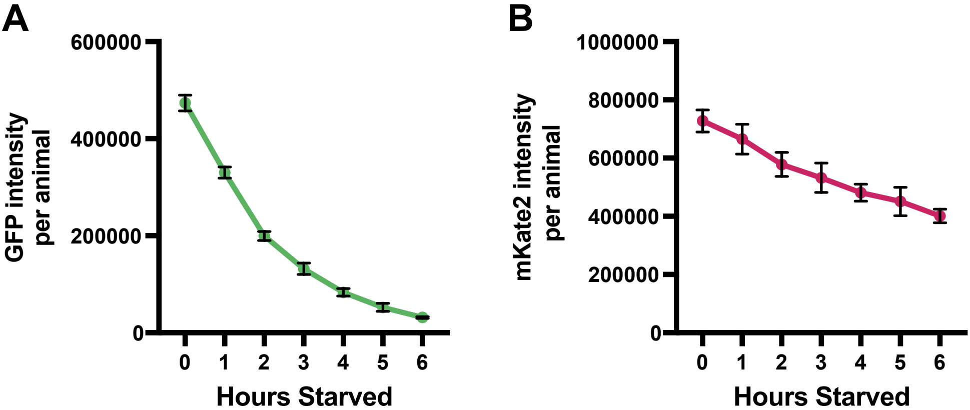
A
600000
400000
200000
0
GFP intensity per animal
0
1
2
3
4
5
6
Hours Starved
B
1000000
800000
600000
400000
200000
0
mKate2 intensity per animal
0
1
2
3
4
5
6
Hours Starved

Figure S5

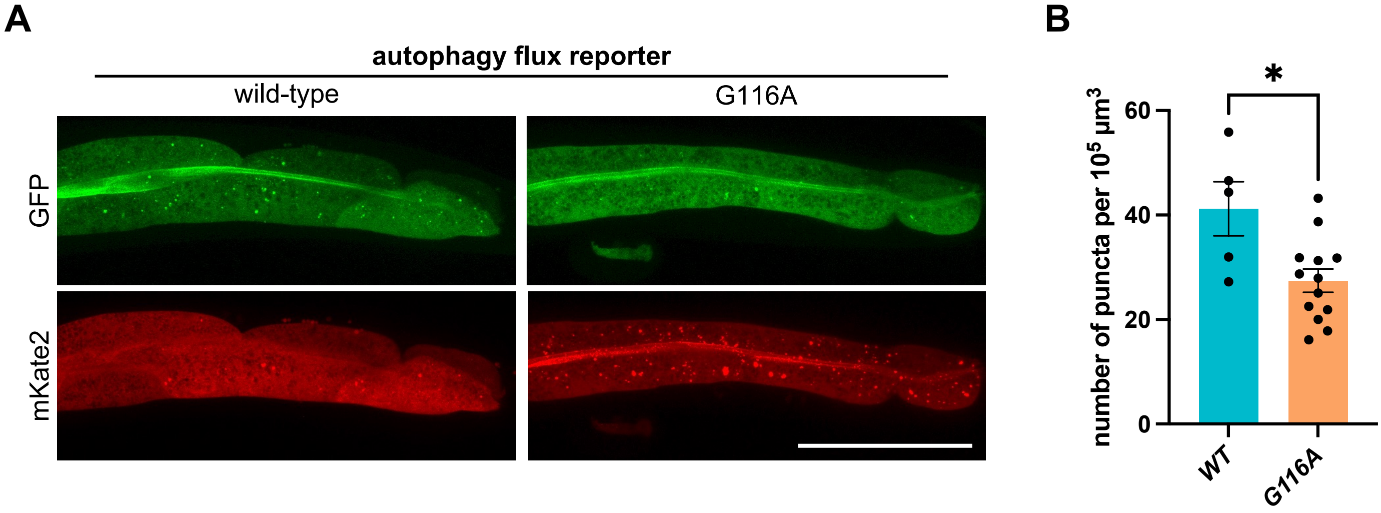

A
autophagy flux reporter
wild-type
G116A
GFP
mKate2
B
*
number of puncta per 10^5 µm^3
60
40
20
0
WT
G116A

Figure S6

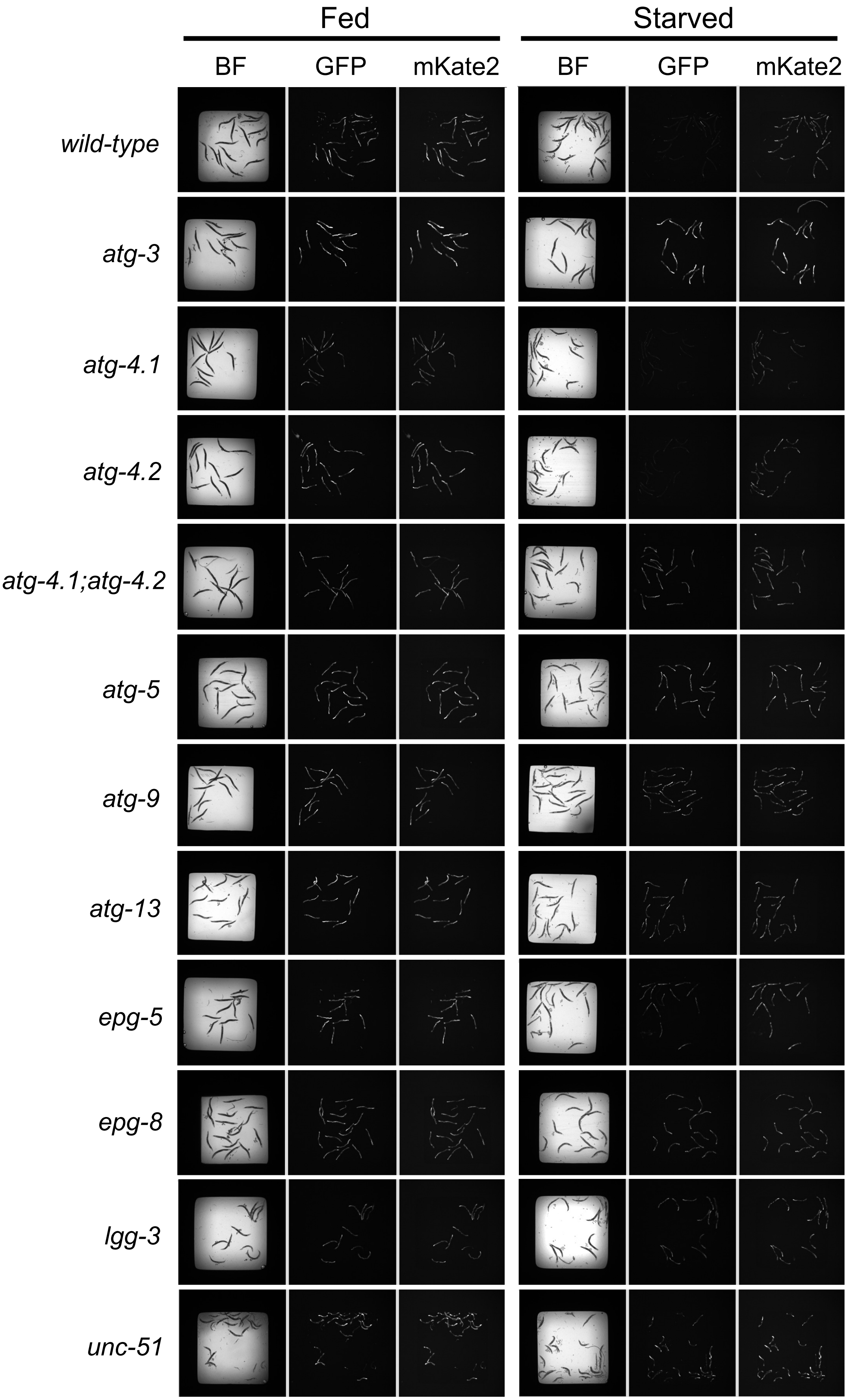

Fed
Starved
BF
GFP
mKate2
BF
GFP
mKate2
wild-type
atg-3
atg-4.1
atg-4.2
atg-4.1;atg-4.2
atg-5
atg-9
atg-13
epg-5
epg-8
lgg-3
unc-51

Figure S7

A

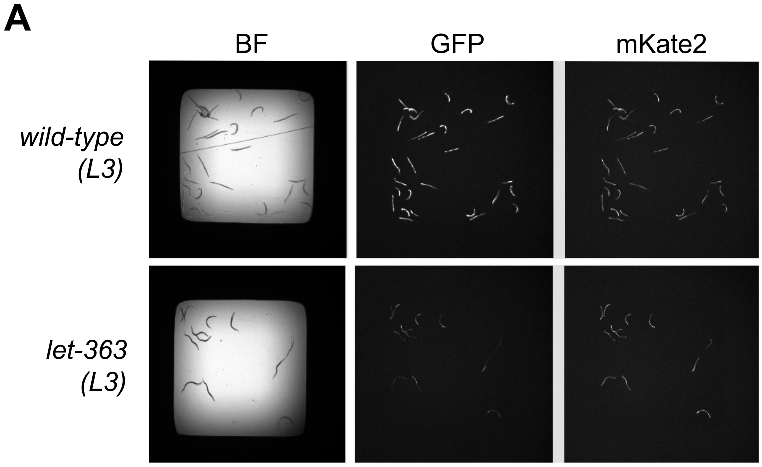
BF
GFP
mKate2
wild-type
(L3)
let-363
(L3)

B

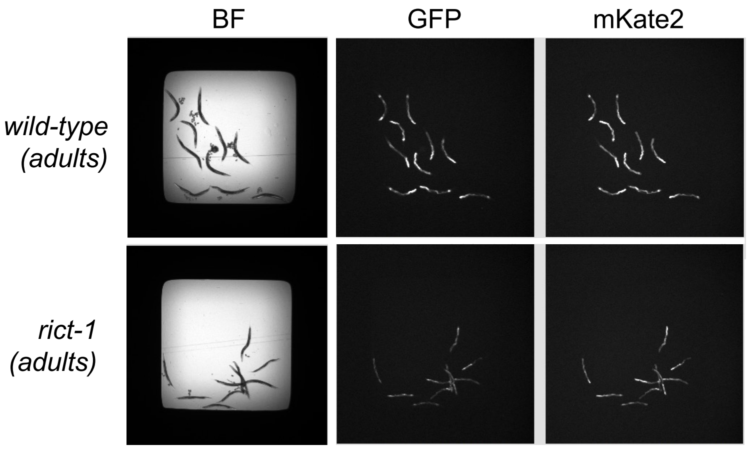
BF
GFP
mKate2
wild-type
(adults)
rict-1
(adults)

Figure S8

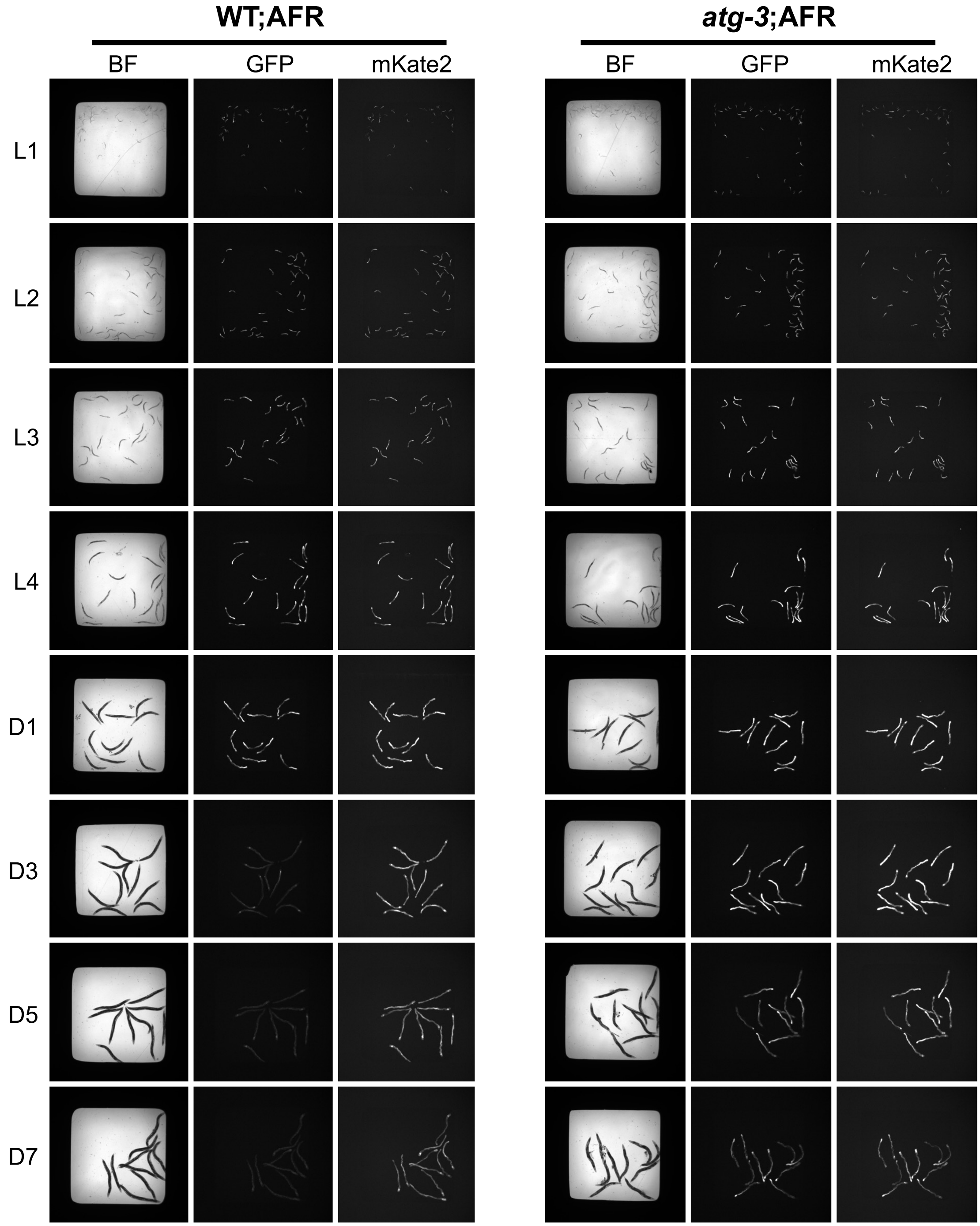
WT;AFR
atg-3;AFR
BF
GFP
mKate2
BF
GFP
mKate2
L1
L2
L3
L4
D1
D3
D5
D7

Figure S9

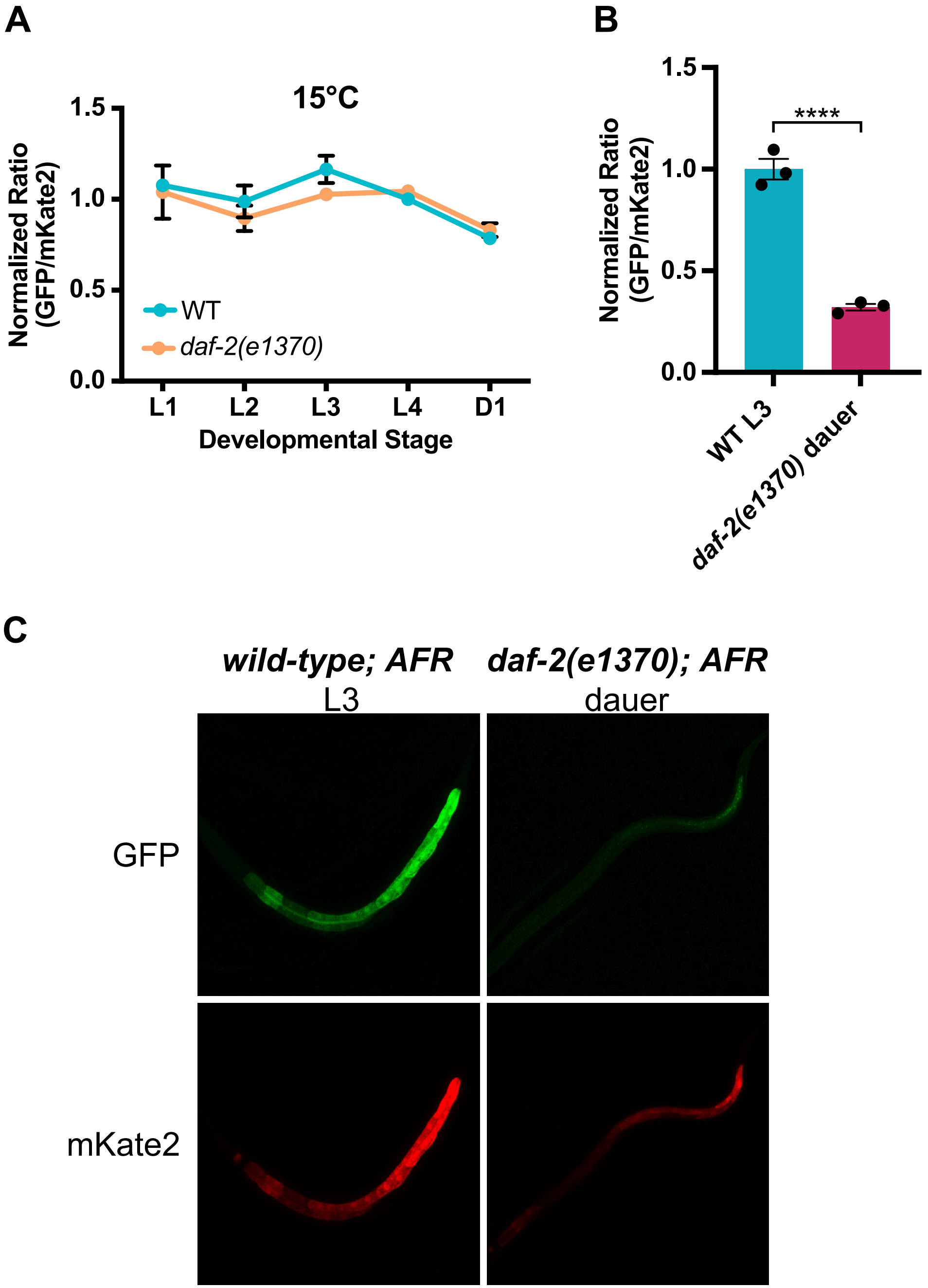
A
15°C
1.5
1.0
0.5
0.0
Normalized Ratio (GFP/mKate2)
WT
daf-2(e1370)
L1
L2
L3
L4
D1
Developmental Stage
B
1.5
1.0
0.5
0.0
Normalized Ratio (GFP/mKate2)
****
WT L3
daf-2(e1370) dauer
C
wild-type; AFR
L3
daf-2(e1370); AFR
dauer
GFP
mKate2

Figure S10

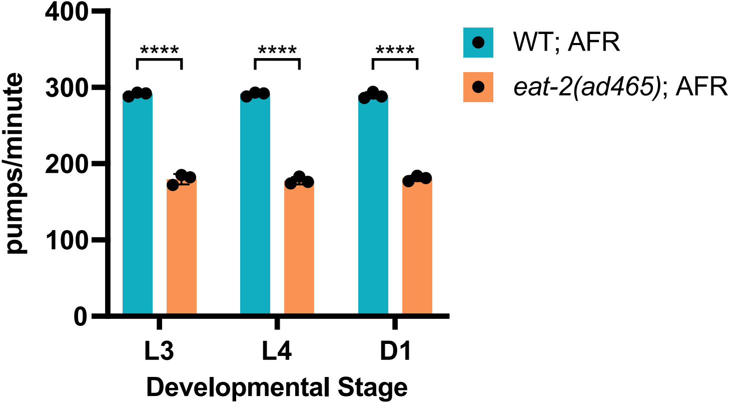

400
300
200
100
0
pumps/minute
****
****
****
L3
L4
D1
Developmental Stage
WT; AFR
eat-2(ad465); AFR

Figure S11

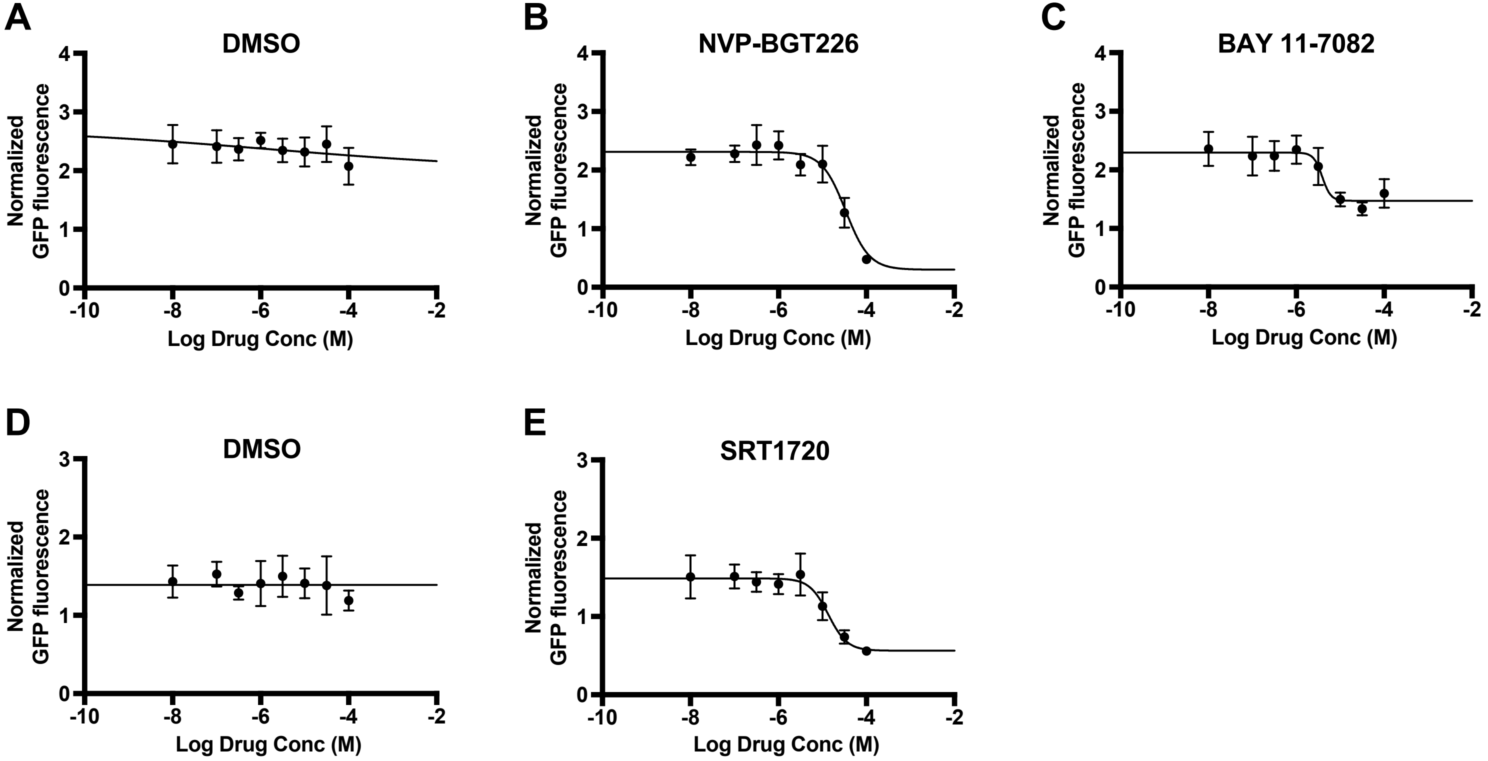
A
DMSO
B
NVP-BGT226
C
BAY 11-7082
D
DMSO
E
SRT1720
Normalized
GFP fluorescence
Log Drug Conc (M)
0
1
2
3
4
-10
-8
-6
-4
-2

Figure S12

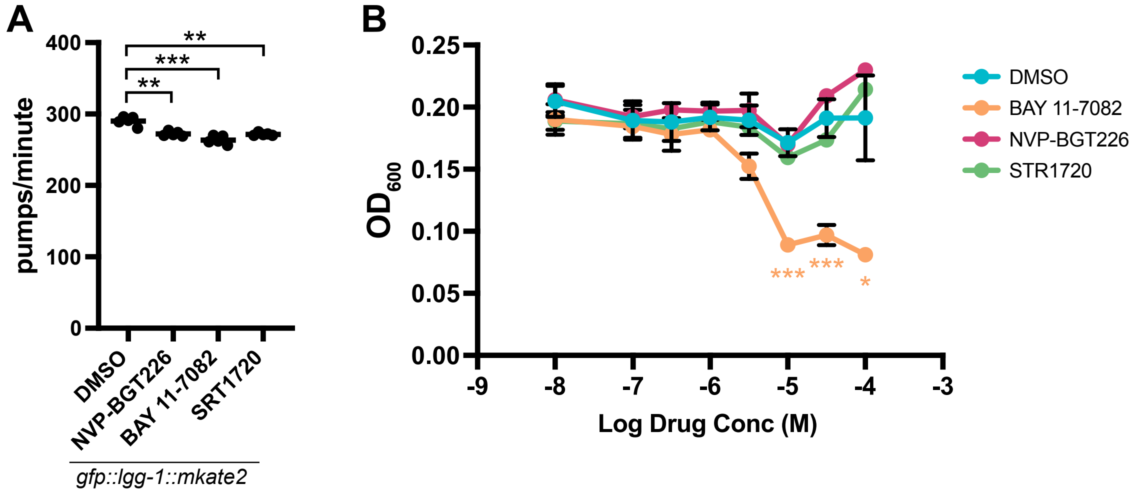
A
400
300
200
100
0
pumps/minute
**
***
**
DMSO
NVP-BGT226
BAY 11-7082
SRT1720
gfp::lgg-1::mkate2
B
0.25
0.20
0.15
0.10
0.05
0.00
OD600
-9
-8
-7
-6
-5
-4
-3
Log Drug Conc (M)
***
***
*
DMSO
BAY 11-7082
NVP-BGT226
STR1720

Figure S13

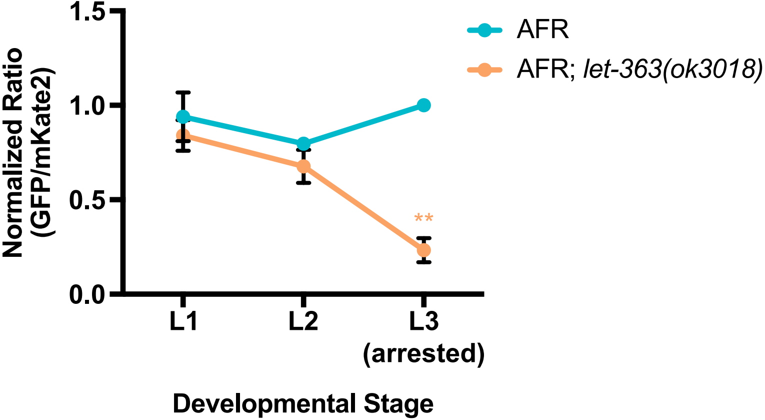

1.5
1.0
0.5
0.0
Normalized Ratio (GFP/mKate2)
L1
L2
L3 (arrested)
Developmental Stage
**
AFR
AFR; let-363(ok3018)
